# Prospective study of circulating metabolomic profiles and breast cancer incidence among predominantly premenopausal women

**DOI:** 10.1038/s41416-025-03159-2

**Published:** 2025-09-04

**Authors:** Tengteng Wang, Oana A. Zeleznik, Emma E. McGee, Kristen D. Brantley, Raji Balasubramanian, Bernard A. Rosner, Walter C. Willett, Julian Avila-Pacheco, Clary B. Clish, A. Heather Eliassen

**Affiliations:** 1 Department of Medicine, Division of Medical Oncology, Section of Cancer Epidemiology and Health Outcomes, Rutgers Robert Wood Johnson Medical School, Rutgers Cancer Institute, New Brunswick, NJ USA; 2 Cancer Prevention and Control Program, Rutgers Cancer Institute, New Brunswick, NJ USA; 3 Department of Biostatistics and Epidemiology, Rutgers School of Public Health, Piscataway, NJ USA; 4 Channing Division of Network Medicine, Department of Medicine, Brigham & Women’s Hospital, and Harvard Medical School, Boston, MA USA; 5 Department of Epidemiology, Harvard T. H. Chan School of Public Health, Boston, MA USA; 6 Eric and Wendy Schmidt Center, Broad Institute of MIT and Harvard, Cambridge, MA USA; 7 Department of Medical Oncology, Dana-Farber Cancer Institute, Boston, MA USA; 8 Department of Biostatistics, School of Public Health & Health Sciences, University of Massachusetts – Amherst, Amherst, MA USA; 9 Department of Biostatistics, Harvard T. H. Chan School of Public Health, Boston, MA USA; 10 Department of Nutrition, Harvard T. H. Chan School of Public Health, Boston, MA USA; 11 Metabolomics Platform, Broad Institute of MIT and Harvard, Cambridge, MA USA

**Keywords:** Cancer epidemiology, Breast cancer

## Abstract

**Background:**

Associations between premenopausal plasma metabolites and breast cancer incidence are largely unknown.

**Methods:**

We conducted a prospective, matched case-control study in which we measured pre-diagnostic metabolomic profiles among predominantly premenopausal women in the Nurses’ Health Study II (*n* = 2010). Lipids, carbohydrates, and organic acid-related metabolites (*n* = 218) were profiled via liquid chromatography-tandem mass spectrometry. Conditional logistic regression was used to estimate odds ratios (OR) for associations between individual metabolites and breast cancer incidence. Associations with metabolite groups were assessed using metabolite set enrichment analysis (MSEA).

**Results:**

Six individual lipid-related metabolites were nominally associated with breast cancer incidence (taurodeoxycholate [OR for per 1 standard deviation increase in metabolite level = 1.15, 95% CI = 1.04–1.28]; C16:1 cholesteryl ester [OR = 0.88, 95% CI = 0.79–0.97]; three phosphocholine (PC)-related metabolites, C34:1 PC [OR = 0.87, 95% CI = 0.78–0.98], C34:3 PC [OR = 0.88, 95% CI = 0.79–0.98], C32:1 PC [OR = 0.88, 95% CI = 0.79–0.98]; indoxyl sulfate [OR = 0.90, 95% CI = 0.82–1.00]). In MSEA analyses, triglycerides (TAGs) with <3 double bonds (normalized enrichment score (NES) = −2.54) and PCs (NES = −2.12) were inversely associated with breast cancer incidence overall and across subgroups. Phosphatidylethanolamine (PE) plasmalogens (NES = 1.83) and PC plasmalogens (NES = 2.23) were positively associated with breast cancer incidence.

**Conclusions:**

Premenopausal plasma TAGs, PCs, and plasmalogen metabolites were associated with breast cancer incidence. Further validation in independent cohorts is warranted.

## Introduction

Breast cancer disease burden is considerable. It is the most common cancer diagnosed among women in 140/180 countries, including U.S. [1]. The etiology of breast cancer is complex, and strong evidence indicates that metabolic processes play a key role [2, 3]. Importantly, associations with established metabolic-related risk factors appear to vary based on menopausal status [4, 5]. For example, obesity is consistently positively associated with postmenopausal breast cancer and inversely associated with premenopausal breast cancer incidence [6, 7]. However, metabolic-related factors and biomarkers are still understudied, especially among premenopausal women. Metabolomics is the comprehensive analysis of small molecules in biological specimens [8]. It can systematically provide a functional readout of upstream changes (genetic, transcriptomic, proteomic) and can reflect potential interaction signals with environmental factors. Therefore, this powerful approach has the potential to offer new insights into the metabolic pathways involved in breast cancer development [5, 8].

Several epidemiological studies have evaluated associations between circulating metabolites and breast cancer incidence [5, 9–23]. However, most studies included only postmenopausal women or did not present results stratified by menopausal status. We previously published a study among predominantly premenopausal women in the Nurses’ Health Study (NHS) II that focused on the circulating amino acid and amino acid-related metabolites only. We observed that 2-aminohippuric acid, DMGV, kynurenic acid, phenylacetylglutamine, and piperine were inversely associated with breast cancer incidence, while creatine and C40:7 PE plasmalogen were positively associated with breast cancer risk [5]. Yet, associations with other metabolite classes, such as circulating lipids, carbohydrates, and organic acids, remain unexplored in NHSII. In this study, we aimed to investigate the associations between pre-diagnostic levels of these metabolites and subsequent incidence of breast cancer among women in the NHSII who were predominately premenopausal at blood collection.

## Methods

### Study population

The NHSII [24] was established in 1989 with 116,429 female registered nurses aged 25–42 years enrolled. Participants were followed by mailed questionnaires every 2 years to collect medical, reproductive, lifestyle, and dietary information. In 1996–1999, 29,611 NHSII participants aged 32–54 years contributed blood samples,18,521 of whom donated timed samples within the menstrual cycle, as previously described [25]. All samples were collected and shipped overnight to the Channing laboratory for further processing and archiving of blood cells and plasma aliquots in liquid nitrogen (<130 °C) freezers [25].

We conducted a matched case-control study nested within the NHSII blood sub-cohort. Eligible women participated in the NHSII blood sub-cohort and were free of cancer (except for nonmelanoma skin cancer) at the time of blood collection. Incident breast cancer cases were identified after blood collection and before 2012. The follow-up in the blood sub-cohort is high (96% in 2011) [25, 26]. The median interval from blood collection to diagnosis was 9 years. Breast cancer cases were reported by the participant and then confirmed by medical record reviews [5, 10]. Controls were selected via risk-set sampling (i.e., incidence density sampling with matching on time). Specifically, one control who was still at risk (not diagnosed with breast cancer) and under follow-up at the time of the case’s diagnosis was individually matched to each breast cancer case on the following factors ascertained at blood collection: age, month, time of day, race/ethnicity, fasting status, luteal day (for samples timed in the menstrual cycle), hours since last meal, and combined menopausal status and postmenopausal hormone use. Additional details on the selection of cases and controls are provided in the Supplemental Methods.

#### Ethics approval and consent to participate

The study protocol was approved by the institutional review boards (IRB No.1999P003389) of the Brigham and Women’s Hospital and Harvard T.H. Chan School of Public Health, and those of participating registries, as required. The informed consent was implied by participants’ return of the questionnaires and blood samples. All study methods were performed in accordance with the relevant recognized ethical guidelines (Declaration of Helsinki).

### Metabolites profiling

Plasma metabolites were profiled at the Broad Institute of MIT and Harvard (Cambridge, MA) using a liquid chromatography-tandem mass spectrometry (LC-MS) method [27–29]. Briefly, for the C8-positive platform, plasma lipids were profiled using a Nexera X2 U-HPLC (Shimadzu Corp.; Marlborough, MA) coupled to a Q Exactive Plus mass spectrometer (Thermo Fisher Scientific; Waltham, MA). Lipids were extracted from plasma (10 µL) using 190 µL of isopropanol containing 1,2-dodecanol-sn-glycerol-3-phosphocholine (Avanti Polar Lipids; Alabaster, AL). After centrifugation, supernatants (2 µL) were injected directly onto a 100 ×2.1 mm, 1.7 µm ACQUITY BEH C8 column (Waters; Milford, MA). MS analyses were carried out using electrospray ionization in the positive ion mode using full scan analysis over 200–1100 m/z. Lipid identities were denoted by the total acyl carbon number and the total number of double bond numbers. For the HILIC-negative platform, HILIC analyses of carbohydrate and organic acids metabolites in the negative ionization mode were conducted using an LC-MS system comprised of a Nexera X2 U-HPLC (Shimadzu Corp.; Marlborough, MA) coupled to a 5500 QTRAP mass spectrometer (Thermo Fisher Scientific; Waltham, MA). Other procedures were similar to the C8-positive platform. Our previously analyzed amino acids and derivatives, which were included in the presented grouped metabolites analysis, were measured through the HILIC-positive ionization mode as previously described [5].

Pooled reference samples were included once for every 20 samples, and results were standardized using the ratio of the value of the sample to the value of the nearest pooled reference, multiplied by the median of all reference values for the metabolite. All samples were run together, with matched case-control pairs (as sets) distributed randomly within the same batch, and the order of the case and controls within each pair randomly assigned. Therefore, the case and its control were always directly adjacent to each other in the analytic run, thereby limiting variability in platform performance across matched case-control pairs [5]. Given this, no additional batch corrections were performed for this present breast cancer case-control project. In addition, 238 quality control (QC) samples, randomly distributed among the samples, were profiled [5]. After excluding broken case-control pairs (*n* = 5), 1055 cases and 1055 matched controls measured on the C8-positive and HILIC-negative platforms were included in our analytical set.

### Covariate information

In order to estimate associations independent of established breast cancer risk factors, we incorporated information on several covariates. Information regarding participant demographic characteristics, reproductive history, medical history, smoking history, weight, height, and physical activity was self-reported and updated on the biennial follow-up questionnaires and at blood collection [25, 26]. Body mass index (BMI, kg/m^2^) was calculated using height (m) reported at baseline and weight (kg) reported at blood collection. Tumor estrogen receptor (ER) expression status was evaluated by immunohistochemistry (IHC) on validated tumor microarrays when possible or extracted from medical records if IHC data were not available [30].

### Statistical analysis

#### Selection of metabolites

We first excluded metabolites that were not stable with the delayed processing inherent in the blood collection methods (*n* = 43) [28]. Moreover, 16 metabolites had ≥10% missing values and were excluded from the main analysis, because these metabolites may not have been sufficiently well-measured. We also excluded non-lipid metabolites included in our previously published paper in the same case-control set (*n* = 9); we did not exclude lipid metabolites included in our previously published paper (*n* = 40), given the focus on lipid metabolites in this analysis [5]. In total, 218 metabolites (C8-positive = 177; HILIC-negative = 41) were included in the primary analysis. All these metabolites exhibited good reproducibility (intraclass correlation coefficient [ICC] range 0.59–1.00) within-person over 1–2 years [28]. Most of the metabolites (*n* = 210) had a coefficient of variation (CV) < 25% and an ICC > 0.4 among QC samples. Of these included metabolites, 152 had no missing values, and 66 metabolites had <10% missing values. Missing values were imputed by one-half of the lowest observed value per metabolite.

#### Analysis of associations

We conducted two types of association analyses: 1) an analysis of individual metabolites and 2) an analysis of groups of metabolites.

In individual metabolite analyses, conditional logistic regression models were used to estimate odds ratios (ORs) and 95% confidence intervals (CIs) for associations between per 1 SD increase in each standardized metabolite level and breast cancer incidence. We fit three different models to investigate the extent to which associations were independent of breast cancer risk factors. In the first model, we did not include additional covariates. In the second model, we adjusted for BMI at age 18 and weight change from age 18 to the time of blood draw. In the third model, the following additional breast cancer risk factors were included: age at menarche, parity, age at first birth, breastfeeding history, family history of breast cancer, personal history of benign breast disease, physical activity, alcohol consumption, and oral contraceptive use.

In analyses of grouped metabolites, we first performed a metabolite set enrichment analysis (MSEA) based on the 218 metabolites that we analyzed individually. The MSEA combined the results from individual metabolites’ logistic regressions by pre-defined groups (annotated at the Broad Institute) to generate a normalized enrichment score (NES) adjusted for metabolites group size [31]. The NES represents the level to which the metabolite set is over-enriched compared to other groups; a positive NES represents a positive association with breast cancer, whereas a negative score indicates a group that is negatively associated with breast cancer [9, 31]. We also evaluated grouped metabolites using a weighted gene co-expression network analysis (WGCNA) approach as the secondary analysis. Here, we also included amino acid-related metabolites that were included in our prior publication[5]. In total, there were 381 metabolites measured across three platforms (C8-positive, HILIC-positive, and HILIC-negative) included in the WGCNA analysis [32]. We followed the methods described in detail previously [9]. Briefly, a metabolite co-expression network is created using data in the controls. The metabolites in the network were clustered using a measure of network proximity. A soft-thresholding power of 3 was selected based on scale-free topology criteria. Modules were then identified using hierarchical clustering and dynamic tree cutting, with a minimum module size of 10 and a merge threshold of 0.25 for closely related modules. Each module was assigned a module score based on the loading on the first principal component of each constituent metabolite, also derived among the controls [9, 32]. Here, the module score is a weighted linear combination of the metabolites included in the module. Module scores were then included in multivariable-adjusted conditional logistic regression models, which were used to estimate associations between each module and incident breast cancer [9, 32].

We also estimated associations within subgroups defined at baseline, including 1) restricting to premenopausal women at blood collection and 2) stratifying by baseline BMI (<25 vs. ≥25 kg/m^2^) and by ER status. In addition, we performed three sensitivity analyses to assess the potential influence of medical and dietary factors, as well as fasting and recency status of blood samples: 1) further adjusting for comorbidities (hypertension and high cholesterol), dietary fat intake, carbohydrate intake, and glycemic index; 2) stratifying by fasting status; and 3) stratifying by median time from blood collection to breast cancer diagnosis (≤6.5 vs. ≥6.5 years) to evaluate whether the associations between identified biomarkers and breast cancer risk varied by proximity to diagnosis.

For each association described above, we reported a continuous *P*-value. We interpreted these values as one piece of evidence within the totality of evidence, including point estimates and confidence intervals. In addition, because investigators are sometimes concerned with potential chance findings when many statistical comparisons are conducted, we estimated adjusted *P*-values that accounted for multiple comparisons using two approaches. For the individual metabolite analyses, we estimated the number of effective tests (NET) as 137 using the standard method [33], which accounts for the correlation structure among metabolites to estimate the number of independent tests, based on the eigenvalues of the correlation matrix of metabolite features. The adjusted *P*-values were further calculated as: *P*_adj_ = *P*_unadjusted_/NET. For the grouped metabolites analyses, we estimated False Discovery Rate (FDR)-adjusted *P*-values based on the q-value procedure [34]. Metabolites with NET-adjusted *p*-value < 0.0004 (0.05/137 = 0.0004) and metabolite sets that met FDR-adjusted *p*-value < 0.05 were considered statistically significant. All statistical tests were two-sided.

## Results

As shown in Table 1, 1055 cases and 1055 matched controls were included in this analysis. The mean age was 45 at blood collection (SD: 4.5), and 77% of women were premenopausal. Cases and controls were generally comparable for most of the characteristics, though cases drank more alcohol, and were more likely to have a personal history of benign breast disease and a family history of breast cancer.

**Table 1:** Baseline characteristics (at the time of blood collection) of eligible breast cancer cases and matched controls, Nurses’ Health Study II (1996–2011)^a^.

	Cases	Controls
	*n* = 1055	*n* = 1055
Age at blood collection, years^b^	44.7 ± 4.5	44.8 ± 4.4
Race/ethnicity^b^		
Non-Hispanic White	1021 (96.8%)	1039 (98.5%)
African American	11 (1.0%)	5 (0.5%)
Asian- American	17 (1.6%)	10 (0.2%)
Other	6 (0.6%)	4 (0.7%)
Fasting at blood collection^b^	725 (68.7%)	789 (74.8%)
Body mass index (BMI), kg/m^2^	25.0 ± 5.2	25.8 ± 6.0
BMI at age 18, kg/m^2^	20.8 ± 2.9	21.1 ± 3.1
Weight change since age 18, kg	11.6 ± 12.0	12.6 ± 13.2
Smoking status		
Never	690 (65.4%)	721 (68.3%)
Past	271 (25.7%)	260 (24.6%)
Current	94 (8.9%)	74 (7.0%)
Physical activity, MET-hours/week	18.1 ± 23.8	18.4 ± 22.7
AHEI diet score	45.6 ± 10.0	45.7 ± 10.4
Alcohol intake, g/day	4.1 ± 7.5	3.5 ± 6.3
Menopausal status and menopausal hormone therapy (MHT) use^b^		
Premenopausal	816 (77.3%)	810 (76.8%)
Postmenopausal, not on MHT	15 (1.4%)	23 (2.2%)
Postmenopausal, on MHT	119 (11.3%)	115 (10.9%)
Unknown	105 (10.0%)	107 (10.1%)
Age at menarche, years	12.4 ± 1.3	12.5 ± 1.4
Parous	832 (78.9%)	887 (84.1%)
Age at first birth^c^		
<25 years	378 (35.8%)	363 (34.4%)
25–30 years	531 (50.4%)	543 (51.5%)
>30 years	146 (13.8%)	149 (14.1%)
Ever breastfed^c^	666 (63.1%)	685 (64.9%)
History of benign breast disease	234 (22.2%)	165 (15.6%)
Family history of breast cancer	183 (17.3%)	114 (10.8%)

*MET* metabolic equivalent of task, *AHEI* Alternative Healthy Eating Index.

^a^Values are means ± SD or numbers (percentages) and are based on those with non-missing values.

^b^Cases and controls were 1:1 matched based on age, month and year of blood collection, time of day of blood draw, fasting status, race/ethnicity, and menopausal status and hormone therapy use at blood draw.

^c^Among parous women.

Six individual metabolites were nominally associated with breast cancer incidence (Table 2 and Supplementary Table 1) across all three models. From the fully adjusted model (model 3), the bile acid taurodeoxycholate, was positively associated with breast cancer risk (OR = 1.15; 95% CI = 1.04–1.28). Remaining metabolites were all inversely associated with overall breast cancer risk, including C16:1 cholesteryl ester (CE) (OR = 0.88; 95% CI = 0.79–0.97), three phosphocholine (PC)-related metabolites (C34:1 PC OR = 0.87; 95% CI = 0.78–0.98; C34:3 PC OR = 0.88; 95% CI = 0.79–0.98; C32:1 PC OR = 0.88; 95% CI = 0.79–0.98), and indoxyl sulfate (OR = 0.90; 95% CI = 0.82–1.00). While the direction and magnitude of associations were generally consistent with the main analysis after restricting the study population to premenopausal women at the time of blood collection (Supplementary Table 2), the associations did not remain statistically significant for C16:1 CE and C32:1 PC (Supplementary Table 3). For subgroup analyses by BMI and ER status, associations for the nominally significant metabolites appeared to vary across subgroups (Supplementary Table 2). For example, among women with BMI < 25 kg/m^2^, associations were strongest for were PC- and CE-related metabolites. Among women with BMI ≥ 25 kg/m^2^, associations were strongest for triglycerides (TAGs) with ≥3 double bonds (DBs) (inverse associations). For ER+ tumors, TAGs with <3 DBs were the top nominally significant ones (inverse associations), while for ER- tumors, three organic acid-relevant metabolites (alpha-keto isovalerate, 2-hydroxyglutarate, indole acetate) were the top ones (positive associations). Individual metabolite results were similar after restricting to women with fasting blood samples and additionally adjusting for comorbidities, dietary fat intake, carbohydrate intake, and dietary glycemic index. The results were also largely consistent across strata defined by time from blood collection to breast cancer diagnosis (≤6.5 years vs. >6.5 years), suggesting that the associations are robust to timing of blood collection and proximity to diagnosis (Supplementary Table 4).

**Table 2:** Odds ratios and 95% confidence intervals for associations between individual metabolites (per 1SD increase in metabolite level) and breast cancer incidence, with metabolites reaching nominal significance, in the Nurses’ Health Study II (1996–2011).

Metabolite	HMDB ID	Class	Model 1 OR (95% CI)	Model 1 *P*-value	Model 2 OR (95% CI)	Model 2 *P*-value	Model 3 OR (95% CI)	Model 3 *P*-value
Taurodeoxycholate	HMDB0000896	Bile acids, alcohols and derivatives	1.15 (1.05–1.26)	0.004	1.15 (1.05–1.27)	0.004	1.15 (1.04–1.28)	0.009
C16:1 CE	HMDB0000658	Steroid esters	0.91 (0.83–0.99)	0.030	0.92 (0.84–1.01)	0.082	0.88 (0.79–0.97)	0.011
C34:1 PC	HMDB0007972	Glycerophosphocholines	0.91 (0.83–1.00)	0.040	0.93 (0.85–1.02)	0.138	0.87 (0.78–0.98)	0.016
C34:3 PC	HMDB0008006	Glycerophosphocholines	0.91 (0.83–1.00)	0.039	0.92 (0.84–1.01)	0.099	0.88 (0.79–0.98)	0.022
C32:1 PC	HMDB0007873	Glycerophosphocholines	0.91 (0.83–0.99)	0.039	0.94 (0.85–1.03)	0.199	0.88 (0.79–0.98)	0.023
Indoxyl sulfate	HMDB0000682	Arylsulfates	0.88 (0.81–0.96)	0.005	0.87 (0.80–0.96)	0.003	0.90 (0.82–1.00)	0.042

*CE* cholesteryl ester, *PC* phosphatidylcholine, *PS* phosphatidylserine, *TAG* triacylglycerides, *OR* odds ratio, *CI* confidence interval.

Model 1: Unadjusted conditional logistic regression model.

Model 2: Model 1+ BMI at age 18, weight change (from age 18 to time of first blood draw).

Model 3: Model 2+ age at menarche, parity and age at first birth, breastfeeding history, family history of breast cancer in a first degree relative, personal history of benign breast disease, physical activity, alcohol intake (by quintile), and oral contraceptive use at blood collection.

**Fig. 1:**
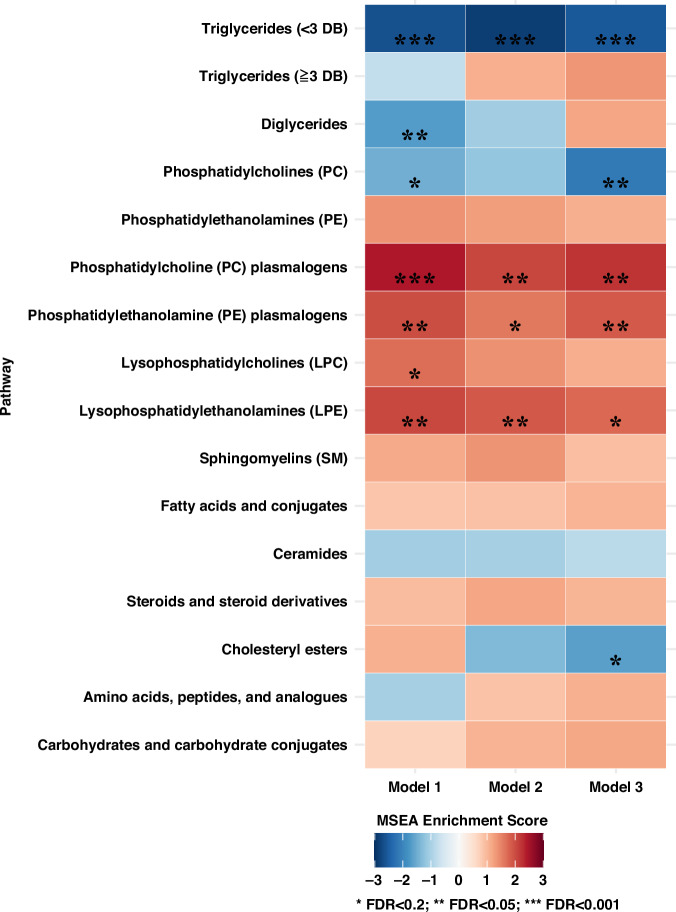
Metabolite set enrichment analysis (MSEA) by class of metabolites (*n* = 218) for overall breast cancer incidence, Nurses’ Health Study II (1996–2011). Stars denote *P* values adjusted by FDR: *P_adj_ < 0.2; **P_adj_ < 0.05; ***P_adj_ < 0.001. Darker blue represents a more negative enrichment score (inverse association with breast cancer); darker red represents a more positive enrichment score (positive association with breast cancer). Model 1: Unadjusted conditional logistic regression model; Model 2: Model 1+ BMI at age 18, weight change (from age 18 to time of first blood draw); Model 3: Model 2+ age at menarche, parity and age at first birth, breastfeeding history, family history of breast cancer in a first degree relative, personal history of benign breast disease, physical activity, alcohol intake (by quintile), and oral contraceptive use at blood collection. DB double bound, FDR false discovery rate.

**Fig. 2:**
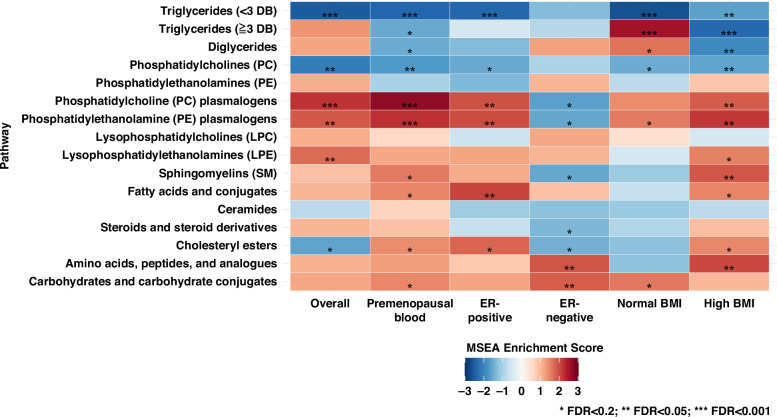
Metabolite set enrichment analysis (MSEA) by class of metabolites (*n* = 218) for breast cancer incidence stratified by subgroups, Nurses’ Health Study II (1996–2011). Stars denote *P* values adjusted by FDR: *P_adj_ < 0.2; **P_adj_ < 0.05; ***P_adj_ <0.001. Darker blue represents a more negative enrichment score (inverse association with breast cancer); darker red represents a more positive enrichment score (positive association with breast cancer). The model for the subgroups is based on the unconditional logistic regression model accounting for matching factors and with further adjustment for BMI at age 18, weight change (from age 18 to time of first blood draw), age at menarche, parity and age at first birth, breastfeeding history, family history of breast cancer in a first degree relative, personal history of benign breast disease, physical activity, alcohol intake (by quintile), and oral contraceptive use at blood collection. DB double bound, FDR false discovery rate.

MSEA identified several classes of metabolites significantly associated with breast cancer incidence after FDR correction (Fig. 1 and Supplementary Table 5). In the fully adjusted model (Fig. 1), TAGs with <3 DBs (normalized enrichment score (NES) = −2.54; *P*_adj_ < 0.00001) and PCs (NES = −2.12; *P*_adj_ = 0.002) were significantly inversely associated with the incidence of overall breast cancer. PC plasmalogens (NES = 2.23; *P*_adj_ < 0.001) and PE plasmalogens (NES = 1.83; *P*_adj_ = 0.02) were positively associated with breast cancer. After stratifying by ER status and BMI and after restricting to premenopausal women at blood draw (Fig. 2), inverse associations with TAGs with <3 DBs were still evident across all subgroups. Interestingly, some classes exhibited associations in opposite directions across different subgroup. For example, TAGs with ≥3 DBs were significantly positively associated with breast cancer among women with BMI < 25 kg/m^2^ (NES = 2.58; *P*_adj_ < 0.001) but inversely associated among those with BMI ≥ 25 kg/m^2^ (NES = −2.35; *P*_adj_ < 0.001) (Supplementary Table 5). Enrichment patterns of other classes, for example, PC plasmalogen, PE plasmalogen, and CE, differed by ER status. Similar findings were also observed in the stratified analyses by fasting blood status and by median time since blood collection.

Nine metabolite modules were identified from the WGCNA analysis (Table 3, Supplementary Tables 6 and 7, and Supplementary Fig. 1) based on the complete set of 381 metabolites across three profiling platforms. Although these modules were not purely defined by a particular class, most modules had one or a few leading classes. For example, module 2 (M2) was characterized by TAGs <3 DBs, which explained 42% of the variation in metabolite levels (Table 3). Modules were modestly associated with overall breast cancer; however, associations were stronger among certain subgroups (Supplementary Table 7). For example, with ER+ breast cancer, module 2 (characterized by TAG < 3 DBs, OR = 0.83; 95% CI = 0.71–0.97), module 5 (characterized by TAG ≥ 3 DBs and amino acids, OR = 0.80; 95% CI = 0.68–0.94), and module 9 (characterized by PC and sphingomyelins, OR = 0.82; 95% CI = 0.70–0.96) were inversely associated with incidence.

**Table 3:** Odds ratios and 95% confidence intervals for associations of WGCNA metabolites (*n* = 381) module scores with breast cancer incidence overall (corresponding to 1 SD increase in score levels), Nurses’ Health Study II (1996–2012).

Modules	Leading class	# of metabolites	Variation explained %	OR (95% CI)^a^	*P*-value	FDR
M1	NA	17	20.2	1.02 (0.91–1.13)	0.76	0.85
M2	TAG < 3 DBs	83	42.0	0.97 (0.87–1.08)	0.54	0.85
M3	Organic acids, PE plasmalogens, PC plasmalogens	73	14.4	0.93 (0.84–1.04)	0.19	0.85
M4	Carnitines, NA	69	11.2	0.98 (0.87–1.10)	0.72	0.85
M5	TAG ≥ 3 DBs, amino acids, NA	37	24.7	0.95 (0.85–1.06)	0.32	0.85
M6	TAG ≥ 3 DBs, PC	30	27.1	1.00 (0.90–1.12)	0.94	0.94
M7	Organic acids and derivatives	27	10.3	1.03 (0.92–1.15)	0.63	0.85
M8	LPC, LPE	24	30.2	1.04 (0.94–1.15)	0.45	0.85
M9	PC, SM	21	37.9	0.97 (0.86–1.08)	0.54	0.85

*TAG* triglyceride, *PE* Phosphatidylethanolamine, *PC* Phosphatidylcholine, *LPC* Phosphatidylcholine, *LPE* Phosphatidylethanolamine, *SM* Sphingomyelins, *DBs* double bounds, *NA* not applicable.

^a^Model is based on conditional logistic regression model accounting for matching factors and with further adjustment for BMI at age 18, weight change (from age 18 to time of first blood draw), age at menarche, parity and age at first birth, breastfeeding history, family history of breast cancer in a first degree relative, personal history of benign breast disease, physical activity, alcohol intake (by quintile), and oral contraceptive use at blood collection.

## Discussion

In this prospective, nested case-control study of predominantly premenopausal (73%) women, we observed that several lipid metabolites from various subclasses were associated with breast cancer incidence. Notably, TAGs with <3 DBs and PCs were inversely associated with breast cancer incidence, both overall and across different subgroups. In contrast, PC plasmalogens and PE plasmalogens were positively associated only with overall breast cancer incidence. The associations of TAGs with ≥3 DBs with breast cancer significantly varied by BMI, and differential associations with PE plasmalogen, PC plasmalogen, and CE were observed by tumor ER status.

TAGs are composed of a glycerol backbone and three fatty acid chains [35]. The fatty acids can have varying degrees of saturation, which refers to the absence (saturated) or presence (unsaturated) of DBs between carbon atoms in the fatty acid chain [35]. We did not observe a strong association between total TAGs and breast cancer. However, classifying by DB number, we observed that TAGs with <3 DBs (*n* = 30, 21% DB = 0, carbon atom range 43–56) were strongly inversely associated with breast cancer incidence. Contrary to this finding, we previously reported that TAGs with <3 DBs were positively associated with breast cancer incidence in an NHS nested case-control study, in which all women were postmenopausal at blood collection [9], which suggests potential heterogeneity of associations by menopausal status. Other relevant studies did not have specific TAG subclass measurements available, or results were not published [11, 12, 16–19, 23], which precludes direct comparisons. While some studies have explored the potential association between blood lipid levels (including TAGs) and breast cancer incidence, findings are conflicting, as summarized in a recent meta-analysis [36].

Our findings on TAG with <3 DBs are also in contrast with the observations from type 2 diabetes studies. Rhee et al. found that TAGs of lower carbon atom number (range 44–50) and DB (range 0-3) content were associated with an increased risk of diabetes in the Framingham Heart Study [37]. This association appeared to be independent of metabolic factors and was confirmed in a recent meta-analysis, with a meaningful trend of higher T2D risk with lower DBs in TAGs [38]. Saturated TAGs (DB = 0) were also reported in our previous work to be associated with lower diet quality and higher red meat and trans-fat intake [39, 40], whereas long-chain TAGs were linked to healthier dietary components (e.g., nuts, whole grains) [39]. In contrast, in the PREDIMED Trial, an opposite finding was observed, where TAGs with <3 DBs and odd-chain were inversely associated with T2D [41]. It has been shown that TAGs with fewer DBs are associated with insulin resistance, and insulin resistance often accompanies metabolic dysregulation (hyperinsulinemia, sex hormone imbalance, chronic inflammation, obesity, etc.) [42]. It is possible that the mixture effects of these pathways are influencing breast carcinogenesis differently among premenopausal women (vs. postmenopausal women), and this is consistent with BMI being inversely associated with breast cancer risk in premenopausal women and the opposite for postmenopausal women. In addition, there is some suggestion from our prior work that metabolic dysregulation-related metabolites (e.g., DMGV and branched-chain amino acid) were inversely associated with breast cancer in premenopausal women [5, 10]. More work is necessary to extend and validate our findings.

We also identified several important associations between PCs (*n* = 21, carbon atom range 30–40, DB range 0–10) and plasmalogens (*n* = 24, carbon atom range 30–40, DB range 0–7) and breast cancer incidence. These metabolites are subclasses of glycerophospholipids that play a crucial role in cell membrane structure and cell signaling [35]. For PCs, our finding of an inverse association with breast cancer was consistent with several cohort studies, including the European Prospective Investigation into Cancer and Nutrition (EPIC) cohort [16, 43], SU.VI.MAX cohort [19], and Cancer Prevention Study (CPS) III [22]. However, levels of PC ae C30:0 were associated with increased breast cancer in EPIC-Heidelberg [23]. The role of PCs in carcinogenesis is not fully understood – our observed inverse association could be related to their anti-inflammatory properties, protection from oxidative stress, and reduction of cell proliferation [44]. For example, His et al. found that better adherence to a healthy lifestyle was associated with higher levels of several PCs [13]. In our prior publication, higher levels of metabolites in PC class were also associated with adiposity [45], as well as higher levels of physical activity [46], carotenoid intake [47], coffee consumption [48], and overall diet quality [39], while being inversely associated with red meat and soda consumption [40, 49, 50].

Similarly, plasmalogens have also been shown to have anti-inflammatory and/or antioxidant functions [41]. While we identified two plasmalogens (head groups are either PC or ethanolamine (PE)) that were positively associated with the incidence of breast cancer, other cohorts of predominantly postmenopausal women, including EPIC, CPSII, and CPSIII identified inverse associations between these metabolites and breast cancer [11, 16, 22]. These findings are challenging to interpret. One hypothesis is that, given that PC and PE plasmalogens comprise ~20% of total human membrane phospholipid [51, 52], higher levels of plasmalogen may indicate changes in the formation and maintenance of cellular membranes [5]. Perturbed membrane metabolism may lead to a corrupted surrounding tissue microenvironment and promote cell proliferation and dissemination [53]. In previous NHS publications, plasmalogens with fewer double bonds were associated with lower overall diet quality and higher intake of red meat and trans fats [39, 40, 49, 54].

Interestingly, we found that the association of TAGs with ≥3 DBs and breast cancer incidence differed by BMI, with a higher incidence among women with normal BMI, but a substantially lower incidence among overweight and obese women. In this same cohort, we previously reported significant inverse associations between erythrocyte membrane n-3 polyunsaturated fatty acids (PUFA) and breast cancer that was only observed among overweight/obese women in the NHSII [55]. Our measured TAGs with ≥3 DBs are composed of long-chain (carbon atoms 52-60) PUFAs, which may indicate potential anti-inflammatory properties. Although we could not determine the number and position of the DBs, it is possible that the anti-inflammatory effects of PUFAs may be more evident in a state of systematic inflammation (e.g., resulting from obesity) than among those with normal BMI [55]. Other differential analyses were also observed for plasmalogen and CE by tumor ER status. Although heterogeneity was not observed in our previous NHS publication [9] or not reported in other studies [11, 12, 16–19, 22, 23], our results may indicate a complex relationship underpinning lipid metabolism, estrogen, and breast cancer development. Because the underlying biological mechanisms are unclear and because we conducted many analyses within each subgroup, it is also important to note that these subgroup findings may be due to chance. Further replication of these results is needed.

Regarding individual metabolites, taurodeoxycholate was the only one to show a suggestive statistically significant association with breast cancer risk after multiple comparison correction in crude and adiposity-adjusted models. This secondary finding may warrant further consideration given prior evidence linking bile acid metabolism to cancer development and progression [56–58]. Taurodeoxycholate, a taurine-conjugated secondary bile acid, has been associated with metabolic and inflammatory pathways that may influence breast carcinogenesis [57, 58]. While our findings should be interpreted with caution due to the lack of robustness after full adjustment, the observed direction of association may suggest a potential biological role that merits investigation in future studies.

Some inconsistencies between our study and other publications may be attributed to multiple factors. First, our study population was predominantly premenopausal at the time of blood collection, while others were mainly composed of postmenopausal women. It is possible that the different findings between lipid metabolites (especially TAG, PC, and plasmalogen) and breast cancer incidence in premenopausal vs. postmenopausal women potentially indicate that these metabolites play distinct metabolic roles at different stages in a woman’s life [45]. Second, it is not straightforward to compare metabolomic profiling platforms across studies, and the associations may depend on platform capacity and, for some metabolites, lipid chains. For example, our measured TAG, PC, and plasmalogen are all long chains with regard to carbon atoms (range 30–60), while others reported significant findings on some short- or medium-chain metabolites (carbon atom <30) [11, 19, 22]. Moreover, the specific timing of blood collection, fasting blood status, and the time from blood draw to diagnosis also differed across studies, though key results remained similar in our sensitivity analyses that investigated variations in associations by these factors.

This study represents a large, prospective investigation of associations between metabolomic profiles and breast cancer incidence among a mixed population that includes 73% premenopausal women. We had relatively broad coverage of multiple metabolite classes, and we were able to assess the extent to which associations were independent of multiple key breast cancer risk factors and metabolic factors. There are also limitations of this study. First, while the platform includes some coverage of amino acids, nucleotides, and other small molecules, it is primarily enriched for lipid-related metabolites; we used a semi-targeted approach, which is fewer than others who have used an untargeted approach (e.g., CPSIII metabolites *n* > 800); therefore, our platform may not provide comprehensive coverage of metabolite classes, e.g., the plasma lipidome. Second, these metabolites were measured at only one point in time. However, overall, the identified metabolites in NHS were relatively stable over time (ICCs or correlation over 1–2 years ≥0.59) [28]. Third, we could not identify specific fatty acid compositions and methyl end locations to subclass PUFA types. Fourth, we could not directly assess the extent to which associations were independent of circulating levels of total cholesterol, HDL cholesterol, LDL cholesterol, and fasting glucose. However, results were similar after adjusting for hyperlipidemia status. Fifth, we aimed to estimate associations between metabolites and breast cancer incidence (rather than the causal effects of well-defined exposures), and our results should be interpreted accordingly [59]. Lastly, an independent validation dataset is lacking here. Further population studies focusing on premenopausal women are needed to validate our findings.

In sum, in this prospective study of metabolomic profiles and breast cancer risk, we found that several lipid metabolites from various subclasses (TAGs with <3 DBs, PCs, and plasmalogens) were associated with breast cancer incidence. Variations of associations with other metabolite classes were observed by adiposity and tumor ER status. Further validation of our findings in independent cohorts of premenopausal women is warranted.

## Supplementary information

Suppl Table 1

Suppl Table 2

Suppl Table 3

Suppl Table 4

Suppl Table 5

Suppl Table 6

Suppl Table 7

Suppl Figure 1

Suppl Method

## Data Availability

Due to participant privacy and data use agreements, our study data are not publicly available. Investigators interested in accessing NHSII data may submit a research proposal to the data access committee via the Nurses’ Health Study website (https://www.nurseshealthstudy.org/researchers). All proposals are subject to review and approval by the NHS steering committee to ensure consistency with participant consent and study policies.

## Code availability

The final analytic programs used to generate derived data and study results are systematically documented and archived within the NHS secure intranet environment to ensure reproducibility. These programs are accessible to both internal and external researchers via the cohort intranet, contingent upon approved user login and data use agreements.
