# Supplementary material for: Prospective study of circulating metabolomic profiles and breast cancer incidence among predominantly premenopausal women
Source: Br J Cancer. Author manuscript; Available in PMC 2025 Dec 6. (PMC12572396; doi:10.1038/s41416-025-03159-2)
Supplement: Suppl Table 1 [file NIHMS2109610-supplement-Suppl_Table_1.pdf]

Supplemental Table 1: Odds ratios and 95% confidence intervals for associations between individual metabolites (per 1 SD increase in metabolite level) and breast cancer incidence, Nurses' Health Study II (1996-2011)

METABOLITE	HMDB_ID	Class (Broad annotation)	OR and 95%CI (model 1)	P-value (model 1)	OR and 95%CI (model 2)	P-value (model 2)	OR and 95%CI (model 3)	P-value (model 3)
**taurodeoxycholate/taurochenodeoxycholate**	HMDB0000896*	alcohols and derivatives	1.15 (1.05-1.26)	0.0039	1.15 (1.05-1.27)	0.0035	1.15 (1.04-1.28)	0.0094
**C16:1 CE**	HMDB0000658*	Steroid esters	0.91 (0.83-0.99)	0.0303	0.92 (0.84-1.01)	0.0818	0.88 (0.79-0.97)	0.0111
**C34:1 PC**	HMDB0007972*	Glycerophosphocholines	0.91 (0.83-1)	0.0395	0.93 (0.85-1.02)	0.1380	0.87 (0.78-0.98)	0.0163
**C34:3 PC**	HMDB0008006*	Glycerophosphocholines	0.91 (0.83-1)	0.0394	0.92 (0.84-1.01)	0.0992	0.88 (0.79-0.98)	0.0222
**C32:1 PC**	HMDB0007873*	Glycerophosphocholines	0.91 (0.83-0.99)	0.0387	0.94 (0.85-1.03)	0.1992	0.88 (0.79-0.98)	0.0233
**indoxylsulfate**	HMDB0000682	Arylsulfates	0.88 (0.81-0.96)	0.0046	0.87 (0.8-0.96)	0.0031	0.9 (0.82-1)	0.0416
**C34:0 PS**	HMDB0012356*	Glycerophosphocholines	0.93 (0.85-1.02)	0.1204	0.95 (0.86-1.05)	0.3005	0.89 (0.8-1)	0.0443
**C58:7 TAG**	HMDB0005471*	Triradylcglycerols (>=3 DB)	1.1 (1.01-1.2)	0.0317	1.09 (1-1.19)	0.0613	1.11 (1-1.23)	0.0491
**C14:0 CE**	HMDB0006725	Steroid esters	0.97 (0.89-1.05)	0.4485	0.95 (0.87-1.03)	0.2226	0.9 (0.82-1)	0.0546
**alpha-keto-beta-methylvalerate/alpha-ketoisocaproate**	HMDB0000491*	.	1.03 (0.94-1.13)	0.5417	1.04 (0.95-1.15)	0.4012	1.11 (0.99-1.24)	0.0630
**C18:3 CE**	HMDB0010370*	Steroid esters	0.96 (0.88-1.05)	0.3493	0.93 (0.85-1.02)	0.1282	0.91 (0.82-1.01)	0.0646
**C36:2 PC plasmalogen**	HMDB0011243*	Glycerophosphocholines	1.11 (1.02-1.22)	0.0176	1.08 (0.99-1.19)	0.0986	1.1 (0.99-1.23)	0.0652
**C40:7 PE plasmalogen**	HMDB0011394*	Glycerophosphocholines	1.12 (1.02-1.23)	0.0143	1.1 (1-1.2)	0.0524	1.1 (0.99-1.23)	0.0715
**C56:5 TAG**	HMDB0005406*	Triradylcglycerols (>=3 DB)	1.06 (0.97-1.16)	0.1906	1.07 (0.98-1.18)	0.1157	1.1 (0.99-1.22)	0.0731
**N-acetylglutamate**	HMDB0001138	Amino acids, peptides, and analogues	1.05 (0.96-1.15)	0.2957	1.08 (0.98-1.18)	0.1310	1.1 (0.99-1.22)	0.0921
**C56:4 TAG**	HMDB0005398*	Triradylcglycerols (>=3 DB)	1.05 (0.96-1.14)	0.3082	1.05 (0.97-1.15)	0.2425	1.09 (0.99-1.2)	0.0940
**kynurenine**	HMDB0000684	Carbonyl compounds	0.88 (0.8-0.97)	0.0092	0.89 (0.81-0.99)	0.0277	0.91 (0.81-1.02)	0.1010
**C34:3 PC plasmalogen**	HMDB0011211*	Glycerophosphocholines	1.1 (1.01-1.2)	0.0351	1.06 (0.97-1.17)	0.2009	1.09 (0.98-1.21)	0.1097
**C34:1 PC plasmalogen-B**	HMDB0011239*	Glycerophosphocholines	1.12 (1.02-1.22)	0.0159	1.09 (0.99-1.2)	0.0851	1.09 (0.98-1.22)	0.1113
**C36:4 DAG**	HMDB0007248*	Lineolic acids and derivatives	1.01 (0.93-1.1)	0.7507	1.03 (0.94-1.12)	0.5202	1.09 (0.98-1.2)	0.1149
**indoleacetate**	HMDB0000197	Pyridinecarboxylic acids and derivatives	0.98 (0.9-1.08)	0.7290	0.97 (0.88-1.06)	0.4669	0.92 (0.84-1.02)	0.1206
**2-aminobutyrate**	HMDB0000650	Amino acids, peptides, and analogues	1.02 (0.93-1.12)	0.6336	1.04 (0.95-1.14)	0.4011	1.09 (0.98-1.2)	0.1226
**C36:3 PC plasmalogen**	HMDB0011244*	Glycerophosphocholines	1.1 (1.01-1.2)	0.0344	1.05 (0.96-1.16)	0.2754	1.09 (0.98-1.21)	0.1253
**C36:1 PC**	HMDB0008038*	Glycerophosphocholines	0.93 (0.85-1.02)	0.1327	0.95 (0.87-1.04)	0.2817	0.92 (0.83-1.02)	0.1255
**C52:1 TAG**	HMDB0005367*	Triradylcglycerols (<3 DB)	0.89 (0.82-0.98)	0.0129	0.93 (0.84-1.02)	0.1347	0.92 (0.82-1.03)	0.1286

**C54:5 TAG**	HMDB0005385*	Triradylcglycerols (>=3 DB)	0.92 (0.84-1)	0.0625	0.95 (0.87-1.05)	0.3343	0.92 (0.83-1.03)	0.1328
**C30:1 PC**	HMDB0007870*	Glycerophosphocholines	0.93 (0.85-1.02)	0.1219	0.96 (0.87-1.05)	0.3350	0.92 (0.83-1.02)	0.1365
**C36:3 DAG**	HMDB0007219*	Lineolic acids and derivatives	0.99 (0.91-1.08)	0.8300	1.02 (0.93-1.11)	0.6924	1.08 (0.97-1.19)	0.1531
**C46:0 TAG**	HMDB0010411*	Triradylcglycerols (<3 DB)	0.93 (0.85-1.01)	0.0789	0.95 (0.86-1.03)	0.2231	0.93 (0.84-1.03)	0.1557
**palmitoylethanolamide**	HMDB0002100	Carboximidic acids	1.02 (0.92-1.12)	0.7497	1.04 (0.94-1.16)	0.4478	1.09 (0.97-1.22)	0.1612
**2-aminoadipate**	HMDB0000510	Amino acids, peptides, and analogues	0.9 (0.81-0.99)	0.0337	0.93 (0.84-1.04)	0.1971	0.92 (0.82-1.03)	0.1643
**C34:4 PC**	HMDB0007883*	Glycerophosphocholines	0.93 (0.85-1.02)	0.1382	0.96 (0.87-1.05)	0.3676	0.93 (0.83-1.03)	0.1701
**C55:3 TAG**	HMDB0042466*	Triradylcglycerols (>=3 DB)	1.05 (0.96-1.14)	0.3077	1.04 (0.95-1.13)	0.4098	1.07 (0.97-1.18)	0.1736
**2-hydroxyglutarate**	HMDB0000694	Fatty acids and conjugates	1.06 (0.96-1.18)	0.2597	1.08 (0.97-1.2)	0.1562	1.09 (0.96-1.22)	0.1745
**C52:7 TAG**	HMDB0010517*	Triradylcglycerols (>=3 DB)	0.93 (0.85-1.02)	0.1272	0.96 (0.87-1.05)	0.3279	0.93 (0.84-1.03)	0.1795
**C50:6 TAG**	HMDB0010497*	Triradylcglycerols (>=3 DB)	0.92 (0.84-1)	0.0585	0.94 (0.86-1.03)	0.2125	0.93 (0.84-1.03)	0.1811
**C34:1 PC plasmalogen**	HMDB0011208*	Glycerophosphocholines	1.11 (1.01-1.21)	0.0220	1.09 (0.99-1.19)	0.0752	1.07 (0.97-1.19)	0.1826
**C18:0 LPC**	HMDB0010384	Glycerophosphocholines	1.06 (0.97-1.17)	0.1778	1.05 (0.96-1.15)	0.3064	1.07 (0.97-1.19)	0.1828
**C50:0 TAG**	HMDB0005357*	Triradylcglycerols (<3 DB)	0.92 (0.85-1.01)	0.0722	0.95 (0.87-1.04)	0.2815	0.93 (0.84-1.04)	0.1856
**C54:4 TAG**	HMDB0005370*	Triradylcglycerols (>=3 DB)	1.03 (0.94-1.12)	0.5132	1.02 (0.94-1.12)	0.6168	1.07 (0.97-1.18)	0.1946
**mesaconate**	HMDB0000749	.	1.06 (0.96-1.16)	0.2608	1.06 (0.96-1.17)	0.2269	1.07 (0.96-1.19)	0.1951
**alpha-ketoisovalerate**	HMDB0000019	Short-chain keto acids and derivatives	1.03 (0.94-1.14)	0.5243	1.06 (0.95-1.17)	0.2844	1.08 (0.96-1.21)	0.2005
**indole-3-propionate**	HMDB0002302	Indolyl carboxylic acids and derivatives	1.07 (0.98-1.17)	0.1180	1.05 (0.96-1.14)	0.3363	1.07 (0.96-1.18)	0.2014
**C48:0 TAG**	HMDB0005356*	Triradylcglycerols (<3 DB)	0.93 (0.85-1.02)	0.1152	0.96 (0.87-1.05)	0.3637	0.93 (0.84-1.04)	0.2044
**C38:7 PE plasmalogen**	HMDB0011420*	Glycerophosphocholines	1.08 (0.99-1.19)	0.0849	1.08 (0.99-1.19)	0.0856	1.07 (0.96-1.2)	0.2061
**xanthurenate**	HMDB0000881	Quinoline carboxylic acids	0.95 (0.87-1.05)	0.3102	0.97 (0.88-1.06)	0.4940	0.93 (0.84-1.04)	0.2085
**C50:5 TAG**	HMDB0010471*	Triradylcglycerols (>=3 DB)	0.92 (0.84-1)	0.0499	0.94 (0.86-1.03)	0.2094	0.94 (0.84-1.04)	0.2095
**C18:0 LPE**	HMDB0011130	Glycerophosphocholines	1.07 (0.98-1.18)	0.1305	1.06 (0.97-1.16)	0.2037	1.07 (0.96-1.19)	0.2105
**C22:0 LPE**	HMDB0011520	Glycerophosphocholines	1.1 (1.01-1.21)	0.0327	1.05 (0.95-1.16)	0.3001	1.07 (0.96-1.2)	0.2133
**C43:1 TAG**	HMDB0042098*	Triradylcglycerols (<3 DB)	0.93 (0.85-1.02)	0.1112	0.95 (0.87-1.04)	0.2948	0.94 (0.85-1.04)	0.2148
**C46:1 TAG**	HMDB0010412*	Triradylcglycerols (<3 DB)	0.91 (0.83-0.99)	0.0318	0.93 (0.85-1.02)	0.1361	0.94 (0.85-1.04)	0.2215
**2-hydroxy-3-methylbutyrate**	HMDB0000407	Fatty acids and conjugates	0.89 (0.82-0.98)	0.0153	0.91 (0.83-1)	0.0531	0.94 (0.84-1.04)	0.2260
**C36:5 PE plasmalogen**	HMDB0011410*	Glycerophosphocholines	1.03 (0.94-1.13)	0.4819	1.04 (0.95-1.14)	0.3797	1.07 (0.96-1.18)	0.2287

**C22:5 CE**	HMDB0010375*	Steroid esters	1.01 (0.92-1.11)	0.8267	0.96 (0.87-1.07)	0.4710	0.93 (0.83-1.05)	0.2314
**C56:7 TAG**	HMDB0005462*	Triradylcglycerols (>=3 DB)	1.05 (0.96-1.15)	0.2824	1.06 (0.97-1.16)	0.2071	1.06 (0.96-1.18)	0.2383
**C54:9 TAG**	HMDB0010498*	Triradylcglycerols (>=3 DB)	0.97 (0.89-1.07)	0.5723	0.99 (0.9-1.08)	0.7661	0.94 (0.85-1.04)	0.2471
**C48:1 TAG**	HMDB0005359*	Triradylcglycerols (<3 DB)	0.92 (0.84-1)	0.0537	0.95 (0.87-1.04)	0.2840	0.94 (0.85-1.04)	0.2483
**C50:2 TAG**	HMDB0005377*	Triradylcglycerols (<3 DB)	0.91 (0.83-0.99)	0.0321	0.95 (0.86-1.05)	0.2965	0.94 (0.84-1.05)	0.2484
**C50:1 TAG**	HMDB0005360*	Triradylcglycerols (<3 DB)	0.91 (0.83-1)	0.0415	0.95 (0.86-1.05)	0.3296	0.94 (0.84-1.05)	0.2487
**C58:6 TAG**	HMDB0005458*	Triradylcglycerols (>=3 DB)	1.05 (0.96-1.15)	0.3085	1.05 (0.96-1.15)	0.2642	1.06 (0.96-1.18)	0.2490
**C22:6 CE**	HMDB0006733	Steroid esters	1.11 (1.02-1.21)	0.0159	1.08 (0.99-1.18)	0.1009	1.06 (0.96-1.18)	0.2500
**campesterol**	HMDB0002869	Ergostane steroids	0.94 (0.85-1.03)	0.1891	0.96 (0.87-1.06)	0.4355	0.94 (0.84-1.05)	0.2509
**cholesterol**	HMDB0000067	Cholestane steroids	0.94 (0.85-1.03)	0.1700	0.96 (0.87-1.06)	0.4597	0.94 (0.84-1.05)	0.2626
**C58:9 TAG**	HMDB0005463*	Triradylcglycerols (>=3 DB)	1.11 (1.02-1.21)	0.0216	1.09 (0.99-1.19)	0.0744	1.06 (0.96-1.18)	0.2634
**C48:2 TAG**	HMDB0005376*	Triradylcglycerols (<3 DB)	0.91 (0.84-0.99)	0.0374	0.94 (0.86-1.03)	0.2075	0.94 (0.85-1.05)	0.2743
**C30:0 PC**	HMDB0007869*	Glycerophosphocholines	0.97 (0.89-1.06)	0.4933	0.98 (0.9-1.08)	0.7316	0.95 (0.85-1.05)	0.2784
**C53:3 TAG**	HMDB0043058*	Triradylcglycerols (>=3 DB)	0.99 (0.91-1.08)	0.8630	1 (0.91-1.09)	0.9408	1.06 (0.96-1.17)	0.2796
**C20:5 CE**	HMDB0006731	Steroid esters	1.01 (0.92-1.1)	0.8982	0.99 (0.91-1.09)	0.8864	0.95 (0.85-1.05)	0.2828
**C34:5 PC plasmalogen**	HMDB0011214*	Glycerophosphocholines	1.04 (0.95-1.14)	0.3633	1.04 (0.95-1.13)	0.4499	1.06 (0.96-1.17)	0.2829
**C34:2 PC plasmalogen**	HMDB0011210*	Glycerophosphocholines	1.11 (1.02-1.22)	0.0151	1.08 (0.98-1.19)	0.1030	1.06 (0.95-1.18)	0.2841
**C38:5 PE plasmalogen**	HMDB0011386*	Glycerophosphocholines	1.03 (0.95-1.13)	0.4668	1.03 (0.94-1.13)	0.5144	1.06 (0.95-1.17)	0.2851
**C22:6 LPE**	HMDB0011526	Glycerophosphocholines	1.09 (0.99-1.2)	0.0675	1.09 (0.99-1.2)	0.0649	1.06 (0.95-1.18)	0.2897
**C54:8 TAG**	HMDB0010518*	Triradylcglycerols (>=3 DB)	0.97 (0.89-1.06)	0.4843	0.98 (0.9-1.07)	0.6486	0.95 (0.86-1.05)	0.2925
**C20:4 LPE**	HMDB0011517	Glycerophosphocholines	1.05 (0.96-1.15)	0.2769	1.05 (0.96-1.15)	0.2643	1.06 (0.95-1.17)	0.2947
**C22:6 LPC**	HMDB0010404	Glycerophosphocholines	1.11 (1.02-1.22)	0.0181	1.09 (0.99-1.2)	0.0716	1.06 (0.95-1.18)	0.3018
**C54:3 TAG**	HMDB0005405*	Triradylcglycerols (>=3 DB)	1 (0.92-1.09)	0.9960	1.02 (0.93-1.11)	0.6801	1.05 (0.95-1.17)	0.3055
**C56:8 TAG**	HMDB0005392*	Triradylcglycerols (>=3 DB)	1.07 (0.98-1.16)	0.1368	1.06 (0.97-1.16)	0.1954	1.05 (0.95-1.16)	0.3074
**C36:1 PE plasmalogen**	HMDB0009016*	Glycerophosphocholines	1.09 (1-1.19)	0.0581	1.05 (0.96-1.15)	0.3098	1.06 (0.95-1.17)	0.3125
**C38:4 PC plasmalogen**	HMDB0011252*	Glycerophosphocholines	1.04 (0.95-1.14)	0.3452	1.03 (0.94-1.13)	0.5596	1.06 (0.95-1.17)	0.3159
**C56:10 TAG**	HMDB0010513*	Triradylcglycerols (>=3 DB)	0.99 (0.9-1.08)	0.7811	1 (0.91-1.09)	0.9372	0.95 (0.85-1.05)	0.3190
**quinolinate**	HMDB0000232	Pyrimidines and pyrimidine derivatives	0.93 (0.85-1.01)	0.0966	0.96 (0.87-1.05)	0.3755	0.95 (0.85-1.05)	0.3266

**C48:3 TAG**	HMDB0005432*	Triradylcglycerols (>=3 DB)	0.91 (0.83-0.99)	0.0267	0.94 (0.85-1.03)	0.1572	0.95 (0.86-1.05)	0.3271
**hexose monophosphate**	HMDB0001401*	.	1.06 (0.96-1.16)	0.2452	1.03 (0.94-1.14)	0.5188	1.06 (0.95-1.18)	0.3316
**C44:0 TAG**	HMDB0042063*	Triradylcglycerols (<3 DB)	0.93 (0.85-1.01)	0.0762	0.95 (0.87-1.03)	0.2240	0.95 (0.86-1.05)	0.3350
**C52:0 TAG**	HMDB0005365*	Triradylcglycerols (<3 DB)	0.93 (0.85-1.01)	0.0907	0.95 (0.87-1.05)	0.3170	0.95 (0.85-1.06)	0.3365
**C36:4 PC plasmalogen**	HMDB0011310*	Glycerophosphocholines	1.1 (1.01-1.2)	0.0360	1.07 (0.97-1.18)	0.1705	1.05 (0.95-1.17)	0.3381
**4-pyridoxate**	HMDB0000017	Pyridinecarboxylic acids and derivatives	0.98 (0.9-1.07)	0.6366	0.98 (0.9-1.07)	0.6755	0.95 (0.86-1.05)	0.3394
**C46:2 TAG**	HMDB0010419*	Triradylcglycerols (<3 DB)	0.92 (0.84-1)	0.0526	0.94 (0.86-1.03)	0.2162	0.95 (0.86-1.05)	0.3411
**C51:3 TAG**	HMDB0011701*	Triradylcglycerols (>=3 DB)	0.97 (0.89-1.06)	0.5254	1 (0.91-1.09)	0.9376	1.05 (0.95-1.17)	0.3445
**C45:1 TAG**	HMDB0042099*	Triradylcglycerols (<3 DB)	0.93 (0.86-1.02)	0.1151	0.95 (0.87-1.04)	0.3030	0.95 (0.86-1.05)	0.3454
**C51:1 TAG**	HMDB0042104*	Triradylcglycerols (<3 DB)	0.93 (0.85-1.01)	0.0851	0.96 (0.87-1.05)	0.3585	0.95 (0.86-1.06)	0.3572
**C51:2 TAG**	HMDB0005362*	Triradylcglycerols (<3 DB)	0.92 (0.84-1)	0.0602	0.95 (0.87-1.04)	0.2493	0.95 (0.86-1.06)	0.3621
**C52:4 TAG**	HMDB0005363*	Triradylcglycerols (>=3 DB)	0.99 (0.91-1.07)	0.7570	1.01 (0.92-1.1)	0.8787	1.05 (0.95-1.16)	0.3630
**C52:6 TAG**	HMDB0005436*	Triradylcglycerols (>=3 DB)	0.94 (0.86-1.03)	0.1724	0.96 (0.88-1.05)	0.4008	0.95 (0.86-1.06)	0.3667
**C20:3 CE**	HMDB0006736*	Steroid esters	0.99 (0.91-1.09)	0.9028	0.98 (0.9-1.07)	0.6943	0.96 (0.87-1.05)	0.3683
**C38:2 PE**	HMDB0008942*	Glycerophosphocholines	1.06 (0.97-1.15)	0.1850	1.04 (0.95-1.13)	0.4427	1.05 (0.95-1.16)	0.3688
**CMPF**	HMDB0061112	Fatty acids and conjugates	1.09 (0.99-1.19)	0.0659	1.07 (0.98-1.18)	0.1288	1.05 (0.94-1.17)	0.3754
**C60:12 TAG**	HMDB0005478*	Triradylcglycerols (>=3 DB)	1.09 (1-1.19)	0.0596	1.08 (0.99-1.18)	0.0973	1.05 (0.94-1.17)	0.3760
**C51:0 TAG**	HMDB0031106*	Triradylcglycerols (<3 DB)	0.95 (0.87-1.03)	0.2250	0.97 (0.88-1.06)	0.4580	0.96 (0.86-1.06)	0.3835
**C38:3 PE plasmalogen**	HMDB0011384*	Glycerophosphocholines	1.06 (0.97-1.15)	0.2229	1.02 (0.93-1.11)	0.7338	1.05 (0.94-1.16)	0.3943
**C34:3 DAG**	HMDB0007132*	Lineolic acids and derivatives	0.96 (0.88-1.05)	0.4105	1 (0.91-1.1)	0.9565	1.05 (0.94-1.16)	0.3958
**C36:4 PC-A**	HMDB0007983*	Glycerophosphocholines	1 (0.91-1.09)	0.9504	0.98 (0.9-1.07)	0.6908	0.96 (0.87-1.06)	0.3984
**C43:2 TAG**	HMDB0043169*	Triradylcglycerols (<3 DB)	0.95 (0.87-1.04)	0.2631	0.98 (0.89-1.07)	0.6322	0.96 (0.86-1.06)	0.4031
**C38:3 PC**	HMDB0008047*	Glycerophosphocholines	0.92 (0.84-1.01)	0.0819	0.96 (0.87-1.06)	0.4736	0.95 (0.85-1.07)	0.4043
**MDA**	HMDB0006112	Carbonyl compounds	1.04 (0.93-1.16)	0.4764	1.05 (0.94-1.17)	0.4013	1.05 (0.93-1.19)	0.4063
**C56:3 TAG**	HMDB0005410*	Triradylcglycerols (>=3 DB)	0.99 (0.9-1.09)	0.8447	1.02 (0.93-1.12)	0.6731	1.05 (0.94-1.16)	0.4086
**phenyllactate**	HMDB0000779	.	1.03 (0.93-1.14)	0.5378	1.02 (0.92-1.13)	0.6606	1.05 (0.93-1.18)	0.4103
**C38:2 PC**	HMDB0008270*	Glycerophosphocholines	0.95 (0.87-1.05)	0.3077	0.98 (0.89-1.07)	0.6353	0.96 (0.86-1.06)	0.4110
**aspartate**	HMDB0000191	Indolyl carboxylic acids and derivatives	1.01 (0.92-1.11)	0.7697	1.04 (0.95-1.15)	0.3975	1.04 (0.94-1.16)	0.4274

**C36:4 PC-B**	HMDB0008138*	Glycerophosphocholines	0.95 (0.87-1.05)	0.3216	0.98 (0.89-1.08)	0.7264	0.96 (0.85-1.07)	0.4309
**3-hydroxymethylglutarate/anhydroDglucose**	HMDB0000355	.	1.04 (0.95-1.15)	0.4038	1.05 (0.95-1.16)	0.3598	1.05 (0.94-1.17)	0.4309
**glycerate**	HMDB0000139	Carbohydrates and carbohydrate conjugates	1.01 (0.92-1.12)	0.8051	1.03 (0.93-1.14)	0.6047	1.05 (0.93-1.17)	0.4433
**C47:2 TAG**	HMDB0042076*	Triradylcglycerols (<3 DB)	0.94 (0.86-1.02)	0.1456	0.96 (0.88-1.05)	0.3998	0.96 (0.87-1.06)	0.4442
**C45:2 TAG**	HMDB0043170*	Triradylcglycerols (<3 DB)	0.95 (0.87-1.03)	0.2103	0.97 (0.89-1.06)	0.5375	0.96 (0.87-1.06)	0.4502
**threitol**	HMDB0004136	Carbohydrates and carbohydrate conjugates	1.06 (0.97-1.16)	0.2099	1.06 (0.97-1.16)	0.2103	1.04 (0.94-1.15)	0.4504
**2-hydroxy-3-methylpentanoate/hydroxyisocaproate**	HMDB0000317*	Fatty acids and conjugates	0.96 (0.88-1.05)	0.3730	0.98 (0.89-1.08)	0.6931	1.04 (0.94-1.16)	0.4571
**C16:0 LPE**	HMDB0011503	Glycerophosphocholines	1.07 (0.98-1.17)	0.1319	1.06 (0.96-1.16)	0.2363	1.04 (0.94-1.16)	0.4614
**C36:2 PE plasmalogen**	HMDB0009082*	Glycerophosphocholines	1.03 (0.94-1.12)	0.5513	1.02 (0.93-1.12)	0.6777	1.04 (0.94-1.15)	0.4699
**C34:3 PE plasmalogen**	HMDB0011343*	Glycerophosphocholines	1.02 (0.94-1.11)	0.6425	1.01 (0.92-1.1)	0.8638	1.04 (0.94-1.15)	0.4702
**C18:2 LPC**	HMDB0010386*	Glycerophosphocholines	1.09 (1-1.2)	0.0517	1.05 (0.96-1.16)	0.2844	1.04 (0.94-1.16)	0.4737
**C16:1 LPC**	HMDB0010383*	Glycerophosphocholines	0.96 (0.88-1.05)	0.3836	0.97 (0.89-1.06)	0.5248	0.96 (0.87-1.07)	0.4797
**C18:1 LPE**	HMDB0011506*	Glycerophosphocholines	1.05 (0.96-1.15)	0.2612	1.04 (0.95-1.14)	0.3908	1.04 (0.94-1.15)	0.4803
**2-aminoheptanoate**	HMDB0094649	.	1.01 (0.92-1.1)	0.8259	1.03 (0.94-1.13)	0.4602	1.04 (0.94-1.15)	0.4921
**C56:6 TAG**	HMDB0005456*	Triradylcglycerols (>=3 DB)	1.03 (0.94-1.12)	0.5664	1.03 (0.94-1.13)	0.4824	1.04 (0.94-1.15)	0.4959
**C54:7 TAG**	HMDB0005447*	Triradylcglycerols (>=3 DB)	0.98 (0.9-1.07)	0.6691	0.98 (0.9-1.07)	0.6484	0.97 (0.88-1.07)	0.5091
**C18:1 SM**	HMDB0012101*	Phosphosphingolipids	0.99 (0.9-1.08)	0.7951	1.03 (0.93-1.13)	0.5895	1.04 (0.93-1.16)	0.5141
**C36:5 PC plasmalogen-B**	HMDB0011220*	Glycerophosphocholines	1.01 (0.93-1.11)	0.7739	1.01 (0.92-1.11)	0.8270	1.03 (0.93-1.15)	0.5142
**C36:3 PE**	HMDB0009060*	Glycerophosphocholines	1.01 (0.93-1.11)	0.7487	1.03 (0.94-1.12)	0.5583	1.03 (0.93-1.14)	0.5182
**C18:2 LPE**	HMDB0011507*	Glycerophosphocholines	1.06 (0.97-1.16)	0.2006	1.04 (0.95-1.14)	0.3578	1.03 (0.93-1.14)	0.5260
**gentisate**	HMDB0000152	Carbohydrates and carbohydrate conjugates	0.95 (0.84-1.09)	0.4772	0.95 (0.84-1.09)	0.4675	0.95 (0.82-1.11)	0.5283
**C34:2 PE plasmalogen**	HMDB0008952*	Glycerophosphocholines	1.03 (0.95-1.13)	0.4529	1.03 (0.94-1.12)	0.5727	1.03 (0.93-1.14)	0.5392
**C38:6 PE plasmalogen**	HMDB0011387*	Glycerophosphocholines	1.02 (0.93-1.11)	0.7356	1.01 (0.93-1.11)	0.7763	1.03 (0.93-1.14)	0.5406
**homovanillate**	HMDB0000118	Methoxyphenols	1 (0.92-1.09)	0.9917	1.02 (0.94-1.12)	0.5984	1.03 (0.93-1.15)	0.5409
**C18:0 SM**	HMDB0001348	Phosphosphingolipids	1.02 (0.93-1.12)	0.6338	1.05 (0.96-1.16)	0.2709	1.03 (0.93-1.15)	0.5441
**C18:1 CE**	HMDB0000918*	Steroid esters	1.04 (0.95-1.14)	0.3772	1 (0.91-1.1)	0.9642	0.97 (0.87-1.08)	0.5493
**coenzyme Q10**	HMDB0001072	.	0.96 (0.88-1.05)	0.3326	0.97 (0.89-1.06)	0.5475	0.97 (0.87-1.08)	0.5496
**C32:0 PC**	HMDB0007871*	Glycerophosphocholines	1.01 (0.93-1.11)	0.7679	1.02 (0.93-1.12)	0.6665	0.97 (0.87-1.08)	0.5508

**C32:0 DAG**	HMDB0007098*	Diacylglycerols	0.94 (0.86-1.02)	0.1479	0.97 (0.88-1.07)	0.5923	0.97 (0.87-1.08)	0.5537
**C22:0 Ceramide (d18:1)**	HMDB0004952	Ceramides	0.92 (0.84-1.01)	0.0667	0.95 (0.87-1.05)	0.3143	0.97 (0.88-1.07)	0.5629
**C40:10 PC**	HMDB0008511*	Glycerophosphocholines	1 (0.91-1.1)	0.9949	1 (0.91-1.09)	0.9433	0.97 (0.87-1.08)	0.5632
**C43:0 TAG**	HMDB0042062*	Triradylcglycerols (<3 DB)	0.96 (0.88-1.05)	0.3587	0.97 (0.89-1.07)	0.5666	0.97 (0.88-1.07)	0.5634
**C20:5 LPC**	HMDB0010397	Glycerophosphocholines	1.09 (1-1.19)	0.0641	1.05 (0.96-1.16)	0.3031	1.03 (0.93-1.15)	0.5697
**C47:1 TAG**	HMDB0042100*	Triradylcglycerols (<3 DB)	0.96 (0.88-1.04)	0.2997	0.97 (0.89-1.06)	0.5592	0.97 (0.88-1.07)	0.5699
**C32:2 PC**	HMDB0007874*	Glycerophosphocholines	0.97 (0.88-1.06)	0.4714	0.99 (0.9-1.09)	0.8522	0.97 (0.88-1.08)	0.5726
**alpha-hydroxybutyrate/beta-hydroxybutyrate/hydroxyisobutyrate**	HMDB0000008*	Alpha hydroxy acids and derivatives	0.97 (0.88-1.06)	0.4917	1.01 (0.91-1.11)	0.8740	1.03 (0.93-1.15)	0.5763
**C58:11 TAG**	HMDB0010531*	Triradylcglycerols (>=3 DB)	1.01 (0.92-1.11)	0.7989	1.02 (0.93-1.12)	0.7050	0.97 (0.87-1.08)	0.5847
**C18:1 LPC**	HMDB0002815*	Glycerophosphocholines	1.05 (0.96-1.15)	0.2711	1.02 (0.93-1.12)	0.6529	1.03 (0.93-1.14)	0.5939
**C16:0 SM**	HMDB0010169	Phosphosphingolipids	1.06 (0.97-1.16)	0.2236	1.05 (0.96-1.15)	0.2783	1.03 (0.93-1.14)	0.5956
**C36:4 PE plasmalogen**	HMDB0011442*	Glycerophosphocholines	1.02 (0.93-1.11)	0.7250	1 (0.91-1.09)	0.9711	1.03 (0.93-1.14)	0.6003
**C14:0 SM**	HMDB0012097	Phosphosphingolipids	0.97 (0.88-1.06)	0.4651	0.99 (0.9-1.08)	0.7998	0.97 (0.88-1.08)	0.6108
**C32:0 PE**	HMDB0008923*	Glycerophosphocholines	0.99 (0.91-1.08)	0.8405	1 (0.91-1.09)	0.9662	0.98 (0.88-1.08)	0.6180
**C36:3 PC**	HMDB0008105*	Glycerophosphocholines	0.95 (0.87-1.04)	0.3021	0.98 (0.89-1.07)	0.6083	0.97 (0.88-1.08)	0.6204
**C36:0 PE**	HMDB0008991*	Glycerophosphocholines	1 (0.92-1.09)	0.9899	0.99 (0.91-1.08)	0.8623	0.98 (0.88-1.08)	0.6230
**malonate**	HMDB0000691	Amino acids, peptides, and analogues	0.95 (0.86-1.05)	0.3446	0.95 (0.86-1.06)	0.3666	0.97 (0.87-1.09)	0.6232
**C52:3 TAG**	HMDB0005384*	Triradylcglycerols (>=3 DB)	0.94 (0.86-1.03)	0.1936	0.99 (0.9-1.08)	0.7862	1.03 (0.92-1.14)	0.6265
**C34:0 PE**	HMDB0008925*	Glycerophosphocholines	1 (0.91-1.09)	0.9362	1 (0.92-1.09)	0.9790	0.98 (0.88-1.08)	0.6277
**C54:1 TAG**	HMDB0005395*	Triradylcglycerols (<3 DB)	0.93 (0.85-1.01)	0.0990	0.96 (0.87-1.05)	0.3741	0.97 (0.88-1.08)	0.6344
**C36:1 DAG**	HMDB0007216*	Diacylglycerols	0.91 (0.84-1)	0.0470	0.95 (0.86-1.05)	0.2954	0.97 (0.87-1.09)	0.6348
**C18:3 LPC**	HMDB0010387*	Glycerophosphocholines	1.03 (0.94-1.13)	0.4686	1.03 (0.94-1.13)	0.5796	1.03 (0.92-1.14)	0.6398
**C18:0 CE**	HMDB0010368	Steroid esters	1 (0.91-1.09)	0.9910	0.99 (0.91-1.09)	0.8906	1.02 (0.92-1.14)	0.6526
**C49:2 TAG**	HMDB0011706*	Triradylcglycerols (<3 DB)	0.95 (0.88-1.04)	0.2856	0.97 (0.89-1.06)	0.5470	0.98 (0.88-1.08)	0.6540
**C52:2 TAG**	HMDB0005369*	Triradylcglycerols (<3 DB)	0.91 (0.83-0.99)	0.0384	0.96 (0.87-1.06)	0.3996	0.97 (0.87-1.09)	0.6551
**uracil**	HMDB0000300	Fatty acids and conjugates	0.96 (0.87-1.06)	0.4144	0.97 (0.88-1.07)	0.5269	0.98 (0.87-1.09)	0.6605
**C38:6 PC**	HMDB0007991*	Glycerophosphocholines	1.07 (0.97-1.17)	0.1719	1.06 (0.97-1.17)	0.2011	1.03 (0.92-1.15)	0.6608
**C24:1 SM**	HMDB0012107*	Phosphosphingolipids	1.08 (0.98-1.18)	0.1230	1.08 (0.98-1.19)	0.1123	1.02 (0.92-1.14)	0.6661

**C50:4 TAG**	HMDB0005435*	Triradylcglycerols (>=3 DB)	0.93 (0.85-1.02)	0.1093	0.96 (0.88-1.06)	0.4297	0.98 (0.88-1.08)	0.6736
**fucose**	HMDB0000174	Amino acids, peptides, and analogues	0.95 (0.87-1.04)	0.2624	0.97 (0.89-1.06)	0.5443	1.02 (0.92-1.13)	0.6744
**C36:5 PC plasmalogen-A**	HMDB0011221*	Glycerophosphocholines	1.01 (0.93-1.1)	0.8196	1.01 (0.92-1.1)	0.8420	0.98 (0.89-1.08)	0.6815
**C36:3 PE plasmalogen**	HMDB0011441*	Glycerophosphocholines	1.02 (0.93-1.11)	0.6600	1 (0.92-1.09)	0.9830	1.02 (0.92-1.13)	0.6820
**C36:2 DAG**	HMDB0007218*	Diacylglycerols	0.94 (0.86-1.03)	0.1819	0.99 (0.9-1.08)	0.7668	1.02 (0.92-1.14)	0.6855
**adipate/methylglutarate**	HMDB0000448*	Fatty acids and conjugates	1.04 (0.95-1.14)	0.4210	1.03 (0.94-1.13)	0.5584	1.02 (0.92-1.13)	0.6966
**C34:2 PC**	HMDB0007973*	Glycerophosphocholines	0.99 (0.9-1.08)	0.8126	1 (0.92-1.1)	0.9220	0.98 (0.88-1.09)	0.6967
**C56:9 TAG**	HMDB0005448*	Triradylcglycerols (>=3 DB)	1.01 (0.93-1.11)	0.7575	1.02 (0.93-1.11)	0.6891	0.98 (0.88-1.09)	0.7067
**C20:4 LPC**	HMDB0010395	Glycerophosphocholines	1.04 (0.95-1.14)	0.3692	1.03 (0.94-1.12)	0.5687	1.02 (0.92-1.13)	0.7082
**C16:0 Ceramide (d18:1)**	HMDB0004949	Ceramides	0.99 (0.9-1.08)	0.7705	1.01 (0.92-1.11)	0.7823	1.02 (0.92-1.13)	0.7169
**C14:0 LPC**	HMDB0010379	Glycerophosphocholines	0.98 (0.9-1.07)	0.6234	0.99 (0.91-1.09)	0.9014	0.98 (0.89-1.08)	0.7177
**C38:4 PC**	HMDB0008048*	Glycerophosphocholines	0.96 (0.88-1.06)	0.4295	0.99 (0.9-1.09)	0.8472	0.98 (0.88-1.09)	0.7245
**suberate**	HMDB0000893	Fatty acids and conjugates	1 (0.9-1.11)	0.9652	1.01 (0.9-1.12)	0.9232	1.02 (0.9-1.16)	0.7248
**pentose monophosphate**	HMDB0001548	.	1.08 (0.98-1.18)	0.1260	1.04 (0.94-1.15)	0.4124	1.02 (0.92-1.13)	0.7330
**C24:0 Ceramide (d18:1)**	HMDB0004956	Ceramides	0.97 (0.89-1.06)	0.5022	1 (0.91-1.09)	0.9439	0.98 (0.89-1.09)	0.7477
**C52:5 TAG**	HMDB0005380*	Triradylcglycerols (>=3 DB)	0.98 (0.9-1.06)	0.5961	0.99 (0.91-1.08)	0.7843	1.02 (0.92-1.12)	0.7486
**C56:2 TAG**	HMDB0005404*	Triradylcglycerols (<3 DB)	0.97 (0.89-1.07)	0.5620	1 (0.91-1.1)	0.9330	1.02 (0.91-1.13)	0.7560
**C38:5 PE**	HMDB0009069*	Glycerophosphocholines	0.99 (0.9-1.08)	0.7944	1 (0.92-1.1)	0.9348	1.02 (0.92-1.13)	0.7768
**C16:0 CE**	HMDB0000885	Steroid esters	1.05 (0.95-1.15)	0.3438	1 (0.91-1.11)	0.9562	0.98 (0.88-1.1)	0.7770
**C34:2 DAG**	HMDB0007103*	Lineolic acids and derivatives	0.94 (0.86-1.03)	0.2060	0.99 (0.9-1.09)	0.7911	1.02 (0.91-1.13)	0.7820
**C40:9 PC**	HMDB0008731*	Glycerophosphocholines	1.06 (0.96-1.16)	0.2415	1.05 (0.96-1.15)	0.3029	1.02 (0.91-1.13)	0.7892
**C38:6 PE**	HMDB0009102*	Glycerophosphocholines	1.02 (0.93-1.12)	0.6516	1.04 (0.95-1.15)	0.4011	1.01 (0.91-1.13)	0.7916
**glutamate**	HMDB0000148	Amino acids, peptides, and analogues	0.98 (0.89-1.08)	0.6685	1.02 (0.92-1.13)	0.7504	1.02 (0.9-1.14)	0.7996
**C34:0 DAG**	HMDB0007100*	Diacylglycerols	0.94 (0.86-1.03)	0.2090	0.98 (0.89-1.08)	0.6624	0.99 (0.89-1.1)	0.8012
**C36:2 PC**	HMDB0008039*	Glycerophosphocholines	1.01 (0.93-1.11)	0.7817	1.02 (0.93-1.12)	0.6257	1.01 (0.92-1.12)	0.8044
**C38:4 PE**	HMDB0009003*	Glycerophosphocholines	0.98 (0.9-1.07)	0.6863	1.02 (0.92-1.12)	0.7443	1.01 (0.91-1.13)	0.8142
**C24:1 Ceramide (d18:1)**	HMDB0004953*	Ceramides	0.97 (0.89-1.06)	0.4795	0.99 (0.91-1.08)	0.8373	0.99 (0.89-1.09)	0.8196
**erythronate/threonate**	HMDB0000613*	Carbohydrates and carbohydrate conjugates	1.02 (0.93-1.11)	0.7168	1 (0.91-1.1)	0.9455	1.01 (0.91-1.12)	0.8319

**C16:0 LPC**	HMDB0010382	Glycerophosphocholines	1.02 (0.93-1.12)	0.6418	1.01 (0.93-1.11)	0.7575	1.01 (0.91-1.12)	0.8373
**adonitol/arabitol**	HMDB0000508*	Carbohydrates and carbohydrate conjugates	1.03 (0.94-1.13)	0.5137	1.03 (0.94-1.13)	0.4932	1.01 (0.91-1.12)	0.8492
**chenodeoxycholate/deoxycholate**	HMDB0000626	alcohols and derivatives	0.97 (0.89-1.06)	0.5307	0.98 (0.9-1.07)	0.7114	0.99 (0.9-1.09)	0.8495
**C22:1 SM**	HMDB0012104*	Phosphosphingolipids	0.99 (0.9-1.09)	0.8593	1.01 (0.92-1.11)	0.8177	1.01 (0.91-1.12)	0.8521
**C54:6 TAG**	HMDB0005391*	Triradylcglycerols (>=3 DB)	1.01 (0.93-1.1)	0.8583	0.99 (0.91-1.08)	0.8246	1.01 (0.91-1.11)	0.8541
**C20:4 CE**	HMDB0006726	Steroid esters	1.04 (0.96-1.14)	0.3522	1.01 (0.93-1.11)	0.7574	1.01 (0.91-1.12)	0.8683
**C54:2 TAG**	HMDB0005403*	Triradylcglycerols (<3 DB)	0.93 (0.85-1.02)	0.1389	0.97 (0.88-1.07)	0.5809	0.99 (0.89-1.11)	0.8778
**C50:3 TAG**	HMDB0005433*	Triradylcglycerols (>=3 DB)	0.93 (0.85-1.01)	0.0985	0.97 (0.88-1.07)	0.5329	0.99 (0.89-1.1)	0.8816
**C36:4 PE**	HMDB0008937*	Glycerophosphocholines	0.99 (0.9-1.08)	0.7717	1.01 (0.92-1.11)	0.7968	0.99 (0.89-1.1)	0.8983
**C55:2 TAG**	HMDB0042226*	Triradylcglycerols (<3 DB)	0.95 (0.87-1.04)	0.3002	0.98 (0.9-1.08)	0.7411	0.99 (0.89-1.11)	0.9154
**C53:2 TAG**	HMDB0042196*	Triradylcglycerols (<3 DB)	0.93 (0.85-1.02)	0.1061	0.96 (0.88-1.05)	0.3691	0.99 (0.9-1.1)	0.9243
**C38:5 DAG**	HMDB0007199*	Diacylglycerols	0.96 (0.88-1.05)	0.3909	0.99 (0.9-1.09)	0.8579	1 (0.91-1.12)	0.9271
**C36:2 PE**	HMDB0008994*	Glycerophosphocholines	0.98 (0.9-1.07)	0.6888	1.01 (0.92-1.11)	0.8496	1 (0.91-1.11)	0.9307
**C20:0 SM**	HMDB0012102	Phosphosphingolipids	1 (0.91-1.1)	0.9681	1.02 (0.93-1.12)	0.6502	1 (0.9-1.12)	0.9354
**3-methyladipate/pimelate**	HMDB0000555*	Fatty acids and conjugates	0.99 (0.89-1.1)	0.8240	1.02 (0.92-1.15)	0.6665	1 (0.89-1.14)	0.9474
**thymine**	HMDB0000262	Pyrimidines and pyrimidine derivatives	1.02 (0.93-1.12)	0.7173	1.01 (0.91-1.11)	0.9071	1 (0.9-1.12)	0.9520
**C34:1 DAG**	HMDB0007102*	Diacylglycerols	0.93 (0.85-1.02)	0.1066	0.97 (0.88-1.07)	0.5654	1 (0.89-1.11)	0.9566
**C56:1 TAG**	HMDB0005396*	Triradylcglycerols (<3 DB)	0.96 (0.88-1.05)	0.3965	0.99 (0.9-1.08)	0.7872	1 (0.9-1.11)	0.9623
**C36:1 PE**	HMDB0008993*	Glycerophosphocholines	0.98 (0.89-1.07)	0.6565	1 (0.91-1.1)	0.9249	1 (0.9-1.11)	0.9660
**C38:7 PC plasmalogen**	HMDB0011229*	Glycerophosphocholines	1.02 (0.93-1.12)	0.6951	1 (0.91-1.1)	0.9964	1 (0.9-1.11)	0.9753
**C40:6 PC**	HMDB0008057*	Glycerophosphocholines	1.03 (0.94-1.13)	0.5387	1.03 (0.94-1.13)	0.4823	1 (0.9-1.11)	0.9756
**C34:2 PE**	HMDB0008928*	Glycerophosphocholines	0.98 (0.9-1.08)	0.7318	1.01 (0.92-1.11)	0.8070	1 (0.9-1.11)	0.9770
**C32:1 DAG**	HMDB0007099*	Diacylglycerols	0.94 (0.86-1.03)	0.1735	0.98 (0.89-1.08)	0.7055	1 (0.9-1.11)	0.9834
**C49:3 TAG**	HMDB0042103*	Triradylcglycerols (>=3 DB)	0.96 (0.88-1.04)	0.3196	0.98 (0.9-1.07)	0.6611	1 (0.9-1.11)	0.9859
**C18:2 CE**	HMDB0000610*	Steroid esters	1.06 (0.96-1.16)	0.2381	1.01 (0.92-1.12)	0.8134	1 (0.9-1.12)	0.9979

Model adjusted for BMI at age 18, weight change (from age 18 to time of first blood draw), age at menarche, parity and age at first birth, breastfeeding history, family history of breast cancer in a first degree relative, personal history of benign breast disease, physical activity, alcohol intake (by quintile), and oral contraceptive use at blood collection.
