# Supplementary material for: Prospective study of circulating metabolomic profiles and breast cancer incidence among predominantly premenopausal women
Source: Br J Cancer. Author manuscript; Available in PMC 2025 Dec 6. (PMC12572396; doi:10.1038/s41416-025-03159-2)
Supplement: Suppl Table 3 [file NIHMS2109610-supplement-Suppl_Table_3.pdf]

Supplemental Table 3. Odds ratios and 95% confidence intervals for associations between individual metabolites (per 1 SD increase in metabolite level) and breast cancer incidence (restricting to premenopausal women at blood collection), with metabolites reaching nominal significance, in the Nurses' Health Study II (1996-2011)

METABOLITE	Full population OR (95% CI)	P-value	Premenopausal population OR (95% CI)	P-value
**Taurodeoxycholate**	1.15 (1.04-1.28)	0.01	1.13 (1-1.27)	0.05
**C16:1 CE**	0.88 (0.79-0.97)	0.01	0.91 (0.81-1.02)	0.11
**C34:1 PC**	0.87 (0.78-0.98)	0.02	0.88 (0.78-0.99)	0.03
**C34:3 PC**	0.88 (0.79-0.98)	0.02	0.87 (0.77-0.97)	0.01
**C32:1 PC**	0.88 (0.79-0.98)	0.02	0.89 (0.79-1.01)	0.07
**Indoxyl sulfate**	0.90 (0.82-1.00)	0.04	0.88 (0.79-0.99)	0.03

Abbreviation: CE: cholesteryl ester; PC: phosphatidylcholine; PS: phosphatidylserine; TAG: triacylglycerides; OR: odds ratio; CI: confidence interval.

Model adjusted for BMI at age 18, weight change (from age 18 to time of first blood draw), age at menarche, parity and age at first birth, breastfeeding history, family history of breast cancer in a first degree relative, personal history of benign breast disease, physical activity, alcohol intake (by quintile), and oral contraceptive use at blood collection.
