# Supplementary material for: Prospective study of circulating metabolomic profiles and breast cancer incidence among predominantly premenopausal women
Source: Br J Cancer. Author manuscript; Available in PMC 2025 Dec 6. (PMC12572396; doi:10.1038/s41416-025-03159-2)
Supplement: Suppl Table 2 [file NIHMS2109610-supplement-Suppl_Table_2.pdf]

Supplemental Table 2. Odds ratios and 95% confidence intervals for associations between individual metabolites (per 1 SD increase in metabolite level) and breast cancer incidence, stratified by subgroups, Nurses' Health Study II (1996-2011).

METABOLITE	Premenopausal at blood collection		Premenopausal at dx		Normal BMI at blood draw		High BMI at blood draw		ER-positive		ER-negative	
	OR.CI	p.value	OR.CI	p.value	OR.CI	p.value	OR.CI	p.value	OR.CI	p.value	OR.CI	p.value
2-aminoadipate	0.93 (0.84-1.04)	0.23	0.93 (0.8-1.09)	0.38	0.89 (0.78-1.01)	0.08	1 (0.85-1.17)	0.97	0.91 (0.81-1.02)	0.11	1.28 (1.04-1.58)	0.02
2-aminobutyrate	1.08 (0.97-1.21)	0.18	1.21 (1.04-1.42)	0.01	1.01 (0.89-1.16)	0.84	1.11 (0.95-1.3)	0.19	1.1 (0.98-1.24)	0.11	1.25 (1.02-1.55)	0.04
2-aminoheptanoate	1.01 (0.91-1.13)	0.82	1.03 (0.88-1.2)	0.72	1.07 (0.94-1.22)	0.28	0.95 (0.82-1.11)	0.55	1.07 (0.95-1.2)	0.25	0.8 (0.65-0.99)	0.04
2-hydroxy-3-methylbutyrate	1 (0.89-1.12)	0.97	1.02 (0.87-1.19)	0.85	0.96 (0.84-1.1)	0.57	0.92 (0.78-1.08)	0.30	1 (0.88-1.12)	0.94	1.23 (1-1.51)	0.05
2-hydroxy-3-methylpentanoate/hydroxyisocaproate	1.11 (0.99-1.24)	0.08	1.09 (0.93-1.28)	0.28	1.01 (0.89-1.16)	0.87	1.05 (0.9-1.23)	0.51	1.13 (1-1.27)	0.04	0.81 (0.66-1)	0.05
2-hydroxyglutarate	1.08 (0.97-1.21)	0.17	1.07 (0.92-1.24)	0.39	1.06 (0.93-1.21)	0.40	1.09 (0.93-1.27)	0.30	1.07 (0.96-1.21)	0.23	1.23 (1-1.52)	0.06
3-hydroxymethylglutarate/anhydroDglucose	1.02 (0.91-1.14)	0.71	1 (0.86-1.17)	0.97	0.93 (0.82-1.06)	0.30	1.18 (1.01-1.39)	0.04	1.08 (0.96-1.22)	0.18	0.82 (0.66-1.02)	0.07
3-methyladipate/pimelate	1 (0.9-1.11)	0.97	0.92 (0.8-1.06)	0.26	0.97 (0.85-1.1)	0.63	1.04 (0.89-1.2)	0.64	1.01 (0.9-1.13)	0.87	0.83 (0.67-1.04)	0.10
4-pyridoxate	0.95 (0.85-1.06)	0.34	0.92 (0.79-1.07)	0.26	0.97 (0.85-1.11)	0.70	0.92 (0.78-1.07)	0.27	0.98 (0.87-1.11)	0.78	0.84 (0.69-1.03)	0.10
adipate/methylglutarate	1.04 (0.93-1.16)	0.49	1.03 (0.88-1.19)	0.73	0.95 (0.84-1.07)	0.39	1.13 (0.97-1.32)	0.12	1.06 (0.95-1.18)	0.32	0.84 (0.68-1.03)	0.10
adonitol/arabitol	1.04 (0.93-1.17)	0.46	0.99 (0.85-1.16)	0.95	1.05 (0.91-1.2)	0.50	0.95 (0.81-1.12)	0.57	1.01 (0.89-1.14)	0.88	0.85 (0.69-1.04)	0.11
alpha-hydroxybutyrate/beta-hydroxybutyrate/hydroxyisobutyrate	1.07 (0.95-1.2)	0.29	1.11 (0.95-1.3)	0.20	1 (0.87-1.14)	0.96	1.1 (0.94-1.29)	0.24	1.01 (0.9-1.14)	0.84	0.84 (0.68-1.04)	0.11
alpha-keto-beta-methylvalerate/alpha-ketoisocaproate	1.12 (1-1.26)	0.05	1.11 (0.95-1.29)	0.20	1.05 (0.92-1.2)	0.43	1.1 (0.94-1.29)	0.22	1.1 (0.98-1.23)	0.12	0.85 (0.69-1.04)	0.12
alpha-ketoisovalerate	1.09 (0.98-1.22)	0.12	1.1 (0.95-1.28)	0.22	1.04 (0.91-1.19)	0.57	1.12 (0.96-1.3)	0.14	1.06 (0.94-1.19)	0.33	0.85 (0.68-1.05)	0.13
aspartate	1.02 (0.92-1.14)	0.70	1.04 (0.9-1.21)	0.59	0.94 (0.82-1.06)	0.31	1.15 (0.98-1.35)	0.08	0.98 (0.87-1.1)	0.70	0.85 (0.69-1.05)	0.13
C14:0 CE	0.97 (0.87-1.08)	0.62	1 (0.87-1.16)	0.96	0.87 (0.77-0.99)	0.04	0.99 (0.85-1.16)	0.92	0.93 (0.83-1.05)	0.23	0.86 (0.7-1.05)	0.15
C14:0 LPC	0.97 (0.87-1.08)	0.57	0.99 (0.86-1.15)	0.93	0.98 (0.86-1.11)	0.73	0.91 (0.78-1.06)	0.23	0.9 (0.8-1.01)	0.07	0.86 (0.69-1.06)	0.15
C14:0 SM	1.01 (0.9-1.13)	0.88	1.09 (0.94-1.28)	0.26	0.96 (0.84-1.1)	0.56	0.99 (0.84-1.16)	0.88	0.95 (0.84-1.07)	0.38	0.86 (0.69-1.06)	0.16
C16:0 CE	1.06 (0.95-1.19)	0.31	1.06 (0.91-1.24)	0.46	0.99 (0.87-1.14)	0.93	1.05 (0.9-1.23)	0.52	1.06 (0.94-1.19)	0.34	0.87 (0.71-1.06)	0.16
C16:0 Ceramide (d18:1)	1.03 (0.92-1.15)	0.56	1.04 (0.89-1.22)	0.59	1 (0.87-1.14)	0.96	0.98 (0.85-1.14)	0.84	0.93 (0.83-1.04)	0.22	1.16 (0.94-1.42)	0.17
C16:0 LPC	0.96 (0.86-1.08)	0.51	0.91 (0.78-1.06)	0.25	0.97 (0.85-1.11)	0.69	0.96 (0.83-1.11)	0.58	0.97 (0.86-1.08)	0.54	1.16 (0.94-1.44)	0.17
C16:0 LPE	0.97 (0.87-1.1)	0.66	0.9 (0.76-1.06)	0.21	0.92 (0.8-1.06)	0.25	1.02 (0.89-1.18)	0.75	0.96 (0.86-1.09)	0.56	0.87 (0.71-1.06)	0.17
C16:0 SM	1.06 (0.95-1.18)	0.30	1.1 (0.94-1.28)	0.22	0.99 (0.87-1.12)	0.90	1.09 (0.94-1.27)	0.25	1.03 (0.92-1.15)	0.61	0.87 (0.71-1.07)	0.18
C16:1 CE	0.91 (0.81-1.02)	0.11	0.85 (0.72-0.99)	0.04	0.83 (0.72-0.95)	0.01	0.95 (0.82-1.11)	0.52	0.89 (0.79-1.01)	0.06	0.86 (0.69-1.07)	0.19
C16:1 LPC	0.94 (0.84-1.05)	0.25	0.88 (0.76-1.03)	0.11	0.92 (0.82-1.05)	0.22	0.96 (0.83-1.11)	0.60	0.94 (0.84-1.05)	0.26	0.87 (0.71-1.08)	0.21
C18:0 CE	1.03 (0.93-1.16)	0.56	1.02 (0.87-1.2)	0.77	1.01 (0.88-1.16)	0.90	1.03 (0.89-1.2)	0.70	1.08 (0.96-1.21)	0.22	0.87 (0.7-1.09)	0.22
C18:0 LPC	1.04 (0.93-1.16)	0.51	1.04 (0.88-1.21)	0.66	1.06 (0.93-1.21)	0.40	1 (0.86-1.15)	0.96	1.02 (0.91-1.15)	0.74	0.88 (0.72-1.08)	0.22
C18:0 LPE	1.02 (0.91-1.14)	0.79	0.97 (0.83-1.13)	0.71	0.99 (0.87-1.13)	0.90	1.01 (0.88-1.17)	0.87	0.98 (0.87-1.1)	0.71	0.88 (0.71-1.09)	0.23

C18:0 SM	1.05 (0.94-1.17)	0.42	1.08 (0.93-1.26)	0.32	0.94 (0.82-1.08)	0.40	1.12 (0.96-1.3)	0.15	0.99 (0.88-1.12)	0.91	1.13 (0.92-1.4)	0.23
C18:1 CE	1.03 (0.92-1.16)	0.59	1.04 (0.88-1.22)	0.65	0.96 (0.83-1.1)	0.55	1.07 (0.92-1.25)	0.39	1.07 (0.95-1.21)	0.25	0.88 (0.71-1.09)	0.24
C18:1 LPC	1.01 (0.9-1.14)	0.81	0.97 (0.83-1.15)	0.74	0.97 (0.85-1.11)	0.66	1.05 (0.89-1.22)	0.57	1.01 (0.9-1.14)	0.84	0.88 (0.71-1.09)	0.24
C18:1 LPE	1.01 (0.9-1.13)	0.83	0.96 (0.82-1.13)	0.65	0.96 (0.85-1.1)	0.58	1.06 (0.9-1.24)	0.49	0.98 (0.87-1.11)	0.78	0.88 (0.71-1.09)	0.24
C18:1 SM	1.04 (0.93-1.16)	0.50	1.06 (0.91-1.24)	0.47	0.94 (0.82-1.07)	0.34	1.14 (0.98-1.33)	0.09	1 (0.89-1.13)	0.94	1.13 (0.92-1.4)	0.24
C18:2 CE	1.06 (0.94-1.19)	0.33	1.09 (0.93-1.28)	0.28	0.99 (0.86-1.13)	0.83	1.08 (0.92-1.27)	0.33	1.08 (0.96-1.22)	0.22	0.88 (0.72-1.09)	0.24
C18:2 LPC	1.02 (0.91-1.16)	0.70	1.03 (0.87-1.22)	0.72	1.01 (0.88-1.15)	0.92	1.01 (0.85-1.19)	0.95	1 (0.88-1.13)	0.98	0.88 (0.72-1.09)	0.25
C18:2 LPE	0.98 (0.88-1.1)	0.78	0.96 (0.83-1.12)	0.64	0.97 (0.85-1.11)	0.67	1 (0.85-1.18)	0.96	0.96 (0.85-1.08)	0.48	0.88 (0.7-1.1)	0.25
C18:3 CE	0.96 (0.86-1.08)	0.51	0.95 (0.82-1.11)	0.53	0.91 (0.8-1.04)	0.16	0.94 (0.81-1.1)	0.45	0.97 (0.86-1.09)	0.61	0.89 (0.72-1.09)	0.26
C18:3 LPC	0.98 (0.88-1.1)	0.78	0.92 (0.79-1.07)	0.30	0.98 (0.86-1.12)	0.77	0.98 (0.85-1.13)	0.79	0.98 (0.88-1.1)	0.77	0.89 (0.72-1.09)	0.26
C20:0 SM	1.03 (0.92-1.16)	0.58	1.19 (1.02-1.4)	0.03	0.99 (0.86-1.13)	0.84	1.03 (0.88-1.2)	0.75	0.99 (0.88-1.12)	0.89	1.13 (0.91-1.41)	0.27
C20:3 CE	1.03 (0.93-1.15)	0.54	1.02 (0.88-1.19)	0.75	0.97 (0.85-1.11)	0.70	1.02 (0.89-1.18)	0.78	1.01 (0.9-1.13)	0.93	0.89 (0.72-1.1)	0.27
C20:4 CE	1.12 (1-1.25)	0.05	1.12 (0.96-1.31)	0.15	1.03 (0.9-1.17)	0.70	1.09 (0.94-1.26)	0.27	1.1 (0.98-1.24)	0.09	0.9 (0.73-1.09)	0.28
C20:4 LPC	1.05 (0.94-1.18)	0.37	1.01 (0.87-1.18)	0.86	1.04 (0.92-1.19)	0.51	0.96 (0.83-1.12)	0.63	1.01 (0.9-1.13)	0.93	0.89 (0.73-1.1)	0.29
C20:4 LPE	1.05 (0.94-1.17)	0.38	0.98 (0.84-1.14)	0.76	1.04 (0.92-1.18)	0.54	0.99 (0.85-1.16)	0.91	1 (0.89-1.12)	0.95	0.89 (0.72-1.1)	0.29
C20:5 CE	1.03 (0.92-1.15)	0.61	1.09 (0.93-1.27)	0.30	0.95 (0.83-1.08)	0.41	0.96 (0.83-1.12)	0.61	1 (0.88-1.12)	0.94	0.89 (0.73-1.1)	0.29
C20:5 LPC	1.01 (0.9-1.15)	0.82	1.02 (0.86-1.2)	0.84	1 (0.87-1.15)	1.00	0.99 (0.84-1.18)	0.94	0.99 (0.87-1.12)	0.89	1.12 (0.91-1.38)	0.30
C22:0 Ceramide (d18:1)	0.96 (0.86-1.07)	0.44	1.04 (0.89-1.22)	0.61	0.98 (0.85-1.12)	0.76	0.91 (0.78-1.06)	0.23	0.92 (0.82-1.04)	0.20	0.9 (0.73-1.11)	0.31
C22:0 LPE	1.04 (0.92-1.17)	0.57	1.02 (0.86-1.2)	0.86	1.04 (0.9-1.2)	0.59	1.02 (0.88-1.2)	0.76	1.03 (0.91-1.16)	0.65	0.9 (0.74-1.1)	0.32
C22:1 SM	1.01 (0.91-1.13)	0.80	1.14 (0.98-1.34)	0.09	0.98 (0.86-1.11)	0.73	1.02 (0.88-1.2)	0.76	0.99 (0.88-1.11)	0.85	0.89 (0.71-1.12)	0.32
C22:5 CE	1.02 (0.91-1.15)	0.68	1.07 (0.91-1.26)	0.41	0.95 (0.83-1.09)	0.46	1.03 (0.87-1.21)	0.75	1.01 (0.89-1.14)	0.88	1.11 (0.9-1.37)	0.33
C22:6 CE	1.13 (1-1.26)	0.04	1.19 (1.02-1.4)	0.03	1.06 (0.92-1.22)	0.44	1.13 (0.97-1.32)	0.12	1.13 (1-1.28)	0.05	0.9 (0.73-1.11)	0.33
C22:6 LPC	1.02 (0.91-1.15)	0.69	1.05 (0.9-1.22)	0.53	1.04 (0.91-1.18)	0.61	0.99 (0.85-1.16)	0.94	0.99 (0.88-1.11)	0.82	0.9 (0.73-1.12)	0.35
C22:6 LPE	1.01 (0.9-1.13)	0.91	1.03 (0.88-1.21)	0.69	1.03 (0.9-1.18)	0.63	0.99 (0.84-1.16)	0.88	0.98 (0.87-1.1)	0.74	0.91 (0.73-1.12)	0.36
C24:0 Ceramide (d18:1)	0.99 (0.88-1.1)	0.82	1.09 (0.93-1.27)	0.27	1 (0.87-1.14)	0.95	0.94 (0.81-1.09)	0.39	0.95 (0.84-1.06)	0.35	0.9 (0.72-1.13)	0.36
C24:1 Ceramide (d18:1)	0.98 (0.88-1.09)	0.68	1.03 (0.88-1.2)	0.71	0.92 (0.8-1.05)	0.21	1.04 (0.9-1.2)	0.59	0.95 (0.85-1.06)	0.36	1.11 (0.89-1.38)	0.37
C24:1 SM	1.04 (0.93-1.16)	0.50	1.09 (0.93-1.28)	0.28	0.97 (0.84-1.11)	0.63	1.09 (0.93-1.28)	0.29	1 (0.89-1.13)	0.99	0.91 (0.73-1.13)	0.37
C30:0 PC	0.97 (0.87-1.09)	0.61	1.02 (0.87-1.19)	0.81	0.93 (0.81-1.06)	0.28	0.97 (0.84-1.13)	0.71	0.87 (0.78-0.99)	0.03	0.91 (0.72-1.13)	0.38
C30:1 PC	0.95 (0.84-1.06)	0.35	0.96 (0.82-1.12)	0.60	0.89 (0.78-1.03)	0.11	0.97 (0.84-1.13)	0.73	0.86 (0.76-0.97)	0.02	1.1 (0.88-1.36)	0.40
C32:0 DAG	0.93 (0.82-1.04)	0.20	0.87 (0.73-1.02)	0.09	0.95 (0.82-1.09)	0.45	0.91 (0.78-1.07)	0.26	0.88 (0.77-0.99)	0.04	0.91 (0.74-1.13)	0.40
C32:0 PC	0.99 (0.89-1.11)	0.91	1 (0.85-1.18)	0.98	0.96 (0.83-1.1)	0.54	0.98 (0.85-1.14)	0.83	0.94 (0.83-1.07)	0.35	0.91 (0.73-1.14)	0.41
C32:0 PE	0.99 (0.89-1.11)	0.93	1.04 (0.9-1.21)	0.59	0.93 (0.82-1.06)	0.28	1.02 (0.88-1.18)	0.81	0.91 (0.81-1.02)	0.10	0.92 (0.74-1.13)	0.41

C32:1 DAG	0.96 (0.85-1.07)	0.44	0.91 (0.77-1.07)	0.26	1 (0.87-1.15)	0.99	0.92 (0.78-1.07)	0.27	0.89 (0.79-1.01)	0.07	0.92 (0.74-1.13)	0.41
C32:1 PC	0.89 (0.79-1.01)	0.07	0.92 (0.77-1.08)	0.30	0.85 (0.73-0.98)	0.03	0.94 (0.8-1.11)	0.48	0.85 (0.75-0.96)	0.01	0.92 (0.75-1.13)	0.42
C32:2 PC	0.96 (0.86-1.08)	0.48	1.03 (0.88-1.21)	0.68	1 (0.87-1.15)	0.99	0.91 (0.78-1.06)	0.22	0.9 (0.8-1.01)	0.08	0.92 (0.75-1.13)	0.42
C34:0 DAG	0.96 (0.86-1.08)	0.50	0.85 (0.72-1)	0.05	0.97 (0.84-1.11)	0.66	0.93 (0.8-1.08)	0.36	0.91 (0.8-1.02)	0.11	0.91 (0.73-1.15)	0.43
C34:0 PE	1 (0.89-1.11)	0.95	1.06 (0.91-1.23)	0.47	0.92 (0.8-1.05)	0.20	1.04 (0.89-1.2)	0.64	0.92 (0.82-1.03)	0.16	0.91 (0.73-1.14)	0.43
C34:0 PS	0.91 (0.81-1.02)	0.10	0.95 (0.82-1.11)	0.54	0.92 (0.8-1.05)	0.20	0.81 (0.69-0.96)	0.01	0.86 (0.76-0.97)	0.02	0.92 (0.75-1.13)	0.43
C34:1 DAG	0.94 (0.83-1.05)	0.28	0.9 (0.76-1.06)	0.20	0.99 (0.86-1.14)	0.84	0.9 (0.77-1.05)	0.20	0.89 (0.78-1)	0.05	0.92 (0.74-1.14)	0.43
C34:1 PC	0.88 (0.78-0.99)	0.03	0.89 (0.76-1.05)	0.19	0.82 (0.71-0.94)	0.01	0.94 (0.81-1.1)	0.46	0.85 (0.75-0.96)	0.01	0.92 (0.74-1.14)	0.44
C34:1 PC plasmalogen	1.1 (0.99-1.23)	0.08	1.14 (0.98-1.32)	0.10	1.03 (0.9-1.16)	0.70	1.17 (1-1.36)	0.05	1.12 (1-1.26)	0.05	0.92 (0.74-1.14)	0.44
C34:1 PC plasmalogen-B	1.09 (0.98-1.21)	0.13	1.13 (0.97-1.32)	0.11	1.03 (0.9-1.16)	0.70	1.12 (0.96-1.31)	0.16	1.09 (0.97-1.22)	0.17	1.09 (0.88-1.35)	0.45
C34:2 DAG	0.93 (0.83-1.04)	0.22	0.9 (0.76-1.06)	0.21	1.01 (0.88-1.15)	0.94	0.91 (0.78-1.07)	0.26	0.92 (0.81-1.03)	0.15	0.92 (0.74-1.15)	0.45
C34:2 PC	0.96 (0.86-1.08)	0.51	1.03 (0.89-1.21)	0.68	0.98 (0.86-1.12)	0.77	0.93 (0.8-1.09)	0.37	0.92 (0.82-1.03)	0.14	0.92 (0.74-1.14)	0.46
C34:2 PC plasmalogen	1.07 (0.96-1.21)	0.23	1.09 (0.93-1.28)	0.27	0.97 (0.85-1.12)	0.71	1.14 (0.98-1.34)	0.10	1.07 (0.95-1.21)	0.27	0.93 (0.76-1.14)	0.47
C34:2 PE	0.96 (0.86-1.08)	0.52	0.97 (0.82-1.13)	0.67	0.97 (0.84-1.12)	0.66	0.98 (0.85-1.14)	0.80	0.92 (0.81-1.03)	0.15	0.93 (0.75-1.14)	0.47
C34:2 PE plasmalogen	1.05 (0.94-1.17)	0.40	1.06 (0.91-1.22)	0.46	1 (0.88-1.14)	0.99	1.06 (0.92-1.22)	0.44	1.04 (0.93-1.17)	0.46	0.93 (0.76-1.14)	0.49
C34:3 DAG	0.95 (0.85-1.07)	0.42	0.94 (0.8-1.1)	0.45	1.07 (0.94-1.23)	0.31	0.9 (0.77-1.06)	0.20	0.96 (0.85-1.09)	0.55	0.93 (0.75-1.15)	0.50
C34:3 PC	0.87 (0.77-0.97)	0.01	0.91 (0.78-1.07)	0.25	0.9 (0.79-1.04)	0.15	0.87 (0.75-1.01)	0.07	0.87 (0.77-0.98)	0.02	0.93 (0.74-1.16)	0.51
C34:3 PC plasmalogen	1.08 (0.97-1.2)	0.17	1.12 (0.97-1.3)	0.12	1.04 (0.91-1.18)	0.55	1.05 (0.9-1.22)	0.57	1.03 (0.92-1.16)	0.62	0.93 (0.76-1.15)	0.51
C34:3 PE plasmalogen	1.04 (0.93-1.15)	0.50	1.03 (0.89-1.19)	0.69	1 (0.88-1.14)	0.96	1.03 (0.9-1.19)	0.65	1 (0.89-1.12)	0.99	0.93 (0.75-1.16)	0.51
C34:4 PC	0.95 (0.84-1.07)	0.38	1.01 (0.86-1.18)	0.94	1 (0.87-1.15)	0.99	0.85 (0.72-0.99)	0.04	0.88 (0.78-0.99)	0.03	1.07 (0.87-1.32)	0.52
C34:5 PC plasmalogen	1.07 (0.96-1.19)	0.20	1.03 (0.89-1.19)	0.71	1.03 (0.91-1.17)	0.64	1.04 (0.9-1.21)	0.62	1.01 (0.9-1.12)	0.92	1.07 (0.86-1.34)	0.52
C36:0 PE	0.98 (0.88-1.09)	0.70	1.03 (0.89-1.19)	0.71	0.91 (0.8-1.04)	0.16	1.01 (0.87-1.17)	0.87	0.9 (0.8-1.01)	0.07	0.93 (0.75-1.16)	0.52
C36:1 DAG	0.92 (0.82-1.04)	0.18	0.89 (0.76-1.05)	0.16	0.95 (0.82-1.09)	0.45	0.89 (0.76-1.05)	0.16	0.88 (0.78-0.99)	0.04	1.07 (0.87-1.31)	0.53
C36:1 PC	0.93 (0.84-1.04)	0.23	0.96 (0.83-1.12)	0.64	0.87 (0.76-1)	0.04	0.97 (0.85-1.12)	0.72	0.9 (0.8-1.01)	0.07	0.93 (0.75-1.16)	0.53
C36:1 PE	0.97 (0.87-1.09)	0.65	1.01 (0.86-1.19)	0.87	0.98 (0.85-1.13)	0.77	0.95 (0.82-1.11)	0.54	0.92 (0.81-1.04)	0.17	0.93 (0.75-1.16)	0.53
C36:1 PE plasmalogen	1.03 (0.92-1.15)	0.61	1.03 (0.88-1.21)	0.69	0.95 (0.83-1.09)	0.48	1.13 (0.96-1.32)	0.14	1.02 (0.91-1.15)	0.72	0.93 (0.75-1.16)	0.53
C36:2 DAG	0.95 (0.85-1.06)	0.37	0.95 (0.8-1.11)	0.50	1.02 (0.89-1.16)	0.82	0.91 (0.78-1.07)	0.26	0.93 (0.83-1.05)	0.26	0.93 (0.75-1.16)	0.54
C36:2 PC	1 (0.89-1.11)	0.95	1.08 (0.93-1.26)	0.31	1.02 (0.9-1.16)	0.73	0.95 (0.82-1.1)	0.48	0.95 (0.85-1.06)	0.34	0.93 (0.75-1.16)	0.54
C36:2 PC plasmalogen	1.11 (1-1.24)	0.05	1.15 (0.99-1.33)	0.08	1.05 (0.92-1.19)	0.48	1.18 (1-1.38)	0.05	1.12 (1-1.26)	0.05	0.94 (0.76-1.16)	0.54
C36:2 PE	0.95 (0.85-1.07)	0.42	0.97 (0.82-1.14)	0.70	1 (0.86-1.15)	0.96	0.92 (0.79-1.07)	0.30	0.91 (0.8-1.03)	0.12	0.94 (0.76-1.16)	0.54
C36:2 PE plasmalogen	1.06 (0.95-1.18)	0.31	1.09 (0.95-1.27)	0.23	1 (0.88-1.14)	0.96	1.04 (0.9-1.21)	0.57	1.03 (0.92-1.15)	0.62	0.93 (0.75-1.16)	0.54
C36:3 DAG	0.97 (0.87-1.08)	0.61	0.99 (0.85-1.16)	0.93	1.1 (0.97-1.26)	0.14	0.91 (0.78-1.07)	0.25	1 (0.89-1.12)	1.00	0.93 (0.74-1.17)	0.55

C36:3 PC	0.95 (0.85-1.06)	0.34	0.98 (0.84-1.15)	0.84	0.99 (0.86-1.13)	0.83	0.94 (0.8-1.09)	0.39	0.94 (0.84-1.06)	0.32	0.94 (0.76-1.16)	0.56
C36:3 PC plasmalogen	1.11 (0.99-1.24)	0.07	1.14 (0.98-1.32)	0.09	1.01 (0.89-1.16)	0.83	1.13 (0.97-1.32)	0.13	1.06 (0.94-1.19)	0.36	0.94 (0.77-1.15)	0.56
C36:3 PE	0.99 (0.89-1.11)	0.92	1.01 (0.87-1.19)	0.86	1.02 (0.89-1.17)	0.78	0.98 (0.84-1.14)	0.80	0.96 (0.85-1.08)	0.52	0.93 (0.73-1.18)	0.56
C36:3 PE plasmalogen	1.04 (0.94-1.16)	0.44	1.05 (0.91-1.21)	0.52	0.98 (0.86-1.11)	0.71	1.05 (0.91-1.22)	0.52	0.99 (0.88-1.11)	0.88	0.94 (0.76-1.16)	0.56
C36:4 DAG	0.99 (0.88-1.1)	0.81	1.01 (0.87-1.18)	0.88	1.17 (1.02-1.33)	0.02	0.88 (0.75-1.03)	0.12	1.03 (0.92-1.16)	0.62	0.94 (0.76-1.16)	0.56
C36:4 PC plasmalogen	1.07 (0.96-1.2)	0.20	1.11 (0.95-1.29)	0.19	0.98 (0.87-1.12)	0.81	1.16 (0.99-1.36)	0.07	1.1 (0.98-1.24)	0.10	1.07 (0.86-1.33)	0.57
C36:4 PC-A	0.94 (0.85-1.05)	0.27	1 (0.86-1.15)	0.95	0.99 (0.87-1.13)	0.91	0.9 (0.77-1.04)	0.15	0.93 (0.83-1.04)	0.22	0.94 (0.75-1.17)	0.57
C36:4 PC-B	1.04 (0.92-1.17)	0.57	1.03 (0.87-1.22)	0.70	1.04 (0.9-1.2)	0.59	0.91 (0.77-1.07)	0.26	0.96 (0.85-1.09)	0.53	1.07 (0.86-1.33)	0.57
C36:4 PE	1.01 (0.9-1.14)	0.88	1 (0.85-1.18)	0.97	1.01 (0.87-1.17)	0.90	0.96 (0.83-1.12)	0.63	0.92 (0.81-1.04)	0.16	1.06 (0.86-1.31)	0.57
C36:4 PE plasmalogen	1.03 (0.93-1.15)	0.54	1.01 (0.87-1.16)	0.91	0.97 (0.85-1.1)	0.64	1.08 (0.93-1.24)	0.32	1.01 (0.9-1.13)	0.87	1.06 (0.86-1.32)	0.57
C36:5 PC plasmalogen-A	0.99 (0.89-1.1)	0.88	1.01 (0.87-1.17)	0.89	0.99 (0.87-1.12)	0.86	0.87 (0.74-1.01)	0.07	0.94 (0.84-1.05)	0.25	0.94 (0.75-1.17)	0.57
C36:5 PC plasmalogen-B	1.06 (0.95-1.18)	0.30	1.05 (0.92-1.21)	0.46	1.06 (0.94-1.2)	0.36	0.95 (0.81-1.11)	0.49	0.99 (0.88-1.11)	0.84	1.06 (0.87-1.29)	0.58
C36:5 PE plasmalogen	1.07 (0.96-1.19)	0.22	1.04 (0.9-1.19)	0.63	1.06 (0.94-1.21)	0.34	1.02 (0.87-1.19)	0.82	1.01 (0.9-1.13)	0.89	1.06 (0.86-1.31)	0.58
C38:2 PC	0.93 (0.83-1.04)	0.21	0.99 (0.84-1.16)	0.87	0.96 (0.83-1.11)	0.61	0.92 (0.79-1.06)	0.24	0.89 (0.79-1.01)	0.06	1.06 (0.86-1.3)	0.58
C38:2 PE	1.01 (0.91-1.13)	0.80	1.13 (0.97-1.31)	0.12	0.99 (0.87-1.12)	0.85	1.02 (0.88-1.19)	0.78	0.95 (0.84-1.06)	0.35	0.94 (0.75-1.17)	0.58
C38:3 PC	0.94 (0.83-1.06)	0.29	0.97 (0.82-1.14)	0.68	1.01 (0.87-1.17)	0.88	0.87 (0.74-1.02)	0.09	0.88 (0.78-1)	0.05	0.94 (0.76-1.17)	0.58
C38:3 PE plasmalogen	1.06 (0.95-1.18)	0.29	1.08 (0.93-1.25)	0.30	1 (0.88-1.14)	0.97	1.04 (0.89-1.2)	0.63	0.99 (0.88-1.11)	0.86	0.94 (0.75-1.18)	0.58
C38:4 PC	1.05 (0.94-1.18)	0.38	1.08 (0.92-1.28)	0.33	1.08 (0.94-1.23)	0.29	0.92 (0.78-1.07)	0.27	0.99 (0.87-1.11)	0.82	1.06 (0.87-1.29)	0.58
C38:4 PC plasmalogen	1.09 (0.98-1.21)	0.13	1.1 (0.95-1.28)	0.22	1.04 (0.92-1.17)	0.57	1.07 (0.91-1.26)	0.42	1.07 (0.95-1.2)	0.26	0.94 (0.77-1.16)	0.58
C38:4 PE	1.02 (0.91-1.14)	0.76	1.03 (0.88-1.22)	0.69	1.05 (0.91-1.22)	0.48	0.94 (0.8-1.09)	0.39	0.94 (0.83-1.07)	0.35	0.94 (0.76-1.17)	0.60
C38:5 DAG	0.98 (0.88-1.1)	0.78	0.95 (0.82-1.12)	0.55	1.07 (0.93-1.22)	0.34	0.87 (0.75-1.02)	0.09	0.97 (0.86-1.09)	0.57	1.05 (0.86-1.29)	0.61
C38:5 PE	1.01 (0.9-1.13)	0.88	1.02 (0.87-1.19)	0.82	1.04 (0.91-1.19)	0.57	0.95 (0.82-1.11)	0.54	0.96 (0.85-1.09)	0.56	0.95 (0.77-1.16)	0.61
C38:5 PE plasmalogen	1.09 (0.98-1.22)	0.11	1.06 (0.92-1.22)	0.45	1.06 (0.93-1.2)	0.40	1.03 (0.88-1.2)	0.73	1.01 (0.9-1.13)	0.93	0.95 (0.77-1.17)	0.61
C38:6 PC	1.02 (0.91-1.15)	0.71	1.1 (0.94-1.29)	0.24	1.05 (0.91-1.2)	0.50	0.95 (0.81-1.13)	0.59	0.99 (0.88-1.12)	0.90	0.94 (0.76-1.18)	0.62
C38:6 PE	1 (0.88-1.12)	0.95	1.07 (0.91-1.26)	0.41	1.04 (0.9-1.2)	0.58	0.95 (0.82-1.12)	0.56	0.95 (0.84-1.08)	0.41	0.95 (0.77-1.17)	0.62
C38:6 PE plasmalogen	1.05 (0.95-1.17)	0.33	1.02 (0.89-1.18)	0.78	1.01 (0.89-1.15)	0.87	1.02 (0.88-1.19)	0.78	0.99 (0.89-1.11)	0.91	0.95 (0.76-1.18)	0.62
C38:7 PC plasmalogen	1 (0.9-1.11)	0.97	1.03 (0.89-1.19)	0.68	0.99 (0.88-1.12)	0.92	0.94 (0.81-1.1)	0.48	0.92 (0.82-1.03)	0.17	0.95 (0.78-1.16)	0.62
C38:7 PE plasmalogen	1.04 (0.93-1.16)	0.46	1.1 (0.95-1.28)	0.21	1.06 (0.93-1.21)	0.35	1 (0.84-1.18)	0.99	1.02 (0.9-1.14)	0.80	0.94 (0.75-1.19)	0.62
C40:10 PC	0.95 (0.85-1.06)	0.35	1.02 (0.88-1.19)	0.80	0.99 (0.87-1.13)	0.94	0.87 (0.74-1.03)	0.10	0.95 (0.85-1.07)	0.40	0.95 (0.77-1.17)	0.63
C40:6 PC	0.99 (0.89-1.11)	0.91	1.07 (0.91-1.25)	0.42	1.03 (0.9-1.17)	0.69	0.95 (0.81-1.11)	0.50	0.96 (0.85-1.08)	0.49	0.95 (0.78-1.17)	0.63
C40:7 PE plasmalogen	1.08 (0.97-1.21)	0.14	1.12 (0.97-1.3)	0.12	1.1 (0.97-1.25)	0.15	1.05 (0.89-1.23)	0.58	1.07 (0.95-1.2)	0.27	0.95 (0.76-1.18)	0.63
C40:9 PC	1.01 (0.91-1.14)	0.80	1.1 (0.94-1.28)	0.25	1.04 (0.91-1.19)	0.57	0.94 (0.79-1.11)	0.44	0.99 (0.87-1.11)	0.82	0.95 (0.76-1.18)	0.64

C43:0 TAG	0.99 (0.88-1.1)	0.80	0.91 (0.78-1.06)	0.21	0.93 (0.81-1.06)	0.26	0.98 (0.84-1.14)	0.80	0.92 (0.82-1.03)	0.15	1.05 (0.85-1.29)	0.64
C43:1 TAG	0.94 (0.84-1.05)	0.30	0.89 (0.76-1.04)	0.15	0.89 (0.77-1.01)	0.08	0.95 (0.82-1.11)	0.54	0.86 (0.77-0.97)	0.02	1.05 (0.85-1.29)	0.64
C43:2 TAG	0.94 (0.84-1.06)	0.33	0.89 (0.75-1.05)	0.16	0.91 (0.79-1.05)	0.20	0.95 (0.81-1.11)	0.51	0.88 (0.78-1)	0.05	0.95 (0.77-1.17)	0.64
C44:0 TAG	0.95 (0.85-1.07)	0.41	0.88 (0.76-1.03)	0.10	0.9 (0.79-1.03)	0.13	0.95 (0.82-1.11)	0.55	0.87 (0.77-0.99)	0.03	1.05 (0.85-1.3)	0.65
C45:1 TAG	0.97 (0.86-1.08)	0.53	0.89 (0.76-1.04)	0.16	0.89 (0.78-1.02)	0.10	0.97 (0.83-1.13)	0.69	0.88 (0.78-0.99)	0.03	1.05 (0.85-1.3)	0.65
C45:2 TAG	0.95 (0.85-1.07)	0.41	0.92 (0.79-1.08)	0.32	0.92 (0.8-1.06)	0.24	0.96 (0.82-1.12)	0.60	0.9 (0.79-1.01)	0.08	0.96 (0.78-1.18)	0.67
C46:0 TAG	0.93 (0.83-1.04)	0.21	0.87 (0.74-1.01)	0.07	0.89 (0.78-1.02)	0.09	0.94 (0.81-1.1)	0.48	0.87 (0.77-0.98)	0.02	0.96 (0.78-1.17)	0.67
C46:1 TAG	0.94 (0.84-1.05)	0.24	0.88 (0.75-1.02)	0.09	0.89 (0.78-1.02)	0.10	0.95 (0.81-1.1)	0.48	0.85 (0.76-0.96)	0.01	0.95 (0.77-1.19)	0.68
C46:2 TAG	0.93 (0.83-1.04)	0.21	0.89 (0.76-1.03)	0.13	0.91 (0.79-1.04)	0.18	0.94 (0.81-1.1)	0.43	0.85 (0.76-0.96)	0.01	0.96 (0.78-1.18)	0.68
C47:1 TAG	0.99 (0.89-1.11)	0.87	0.94 (0.81-1.09)	0.41	0.91 (0.8-1.04)	0.17	1.01 (0.86-1.18)	0.93	0.9 (0.8-1.01)	0.08	0.96 (0.77-1.18)	0.68
C47:2 TAG	0.96 (0.86-1.08)	0.50	0.92 (0.79-1.07)	0.30	0.91 (0.8-1.04)	0.17	0.97 (0.83-1.13)	0.67	0.88 (0.78-0.99)	0.03	0.95 (0.76-1.19)	0.68
C48:0 TAG	0.93 (0.83-1.05)	0.25	0.89 (0.76-1.05)	0.17	0.89 (0.77-1.02)	0.09	0.96 (0.82-1.12)	0.58	0.86 (0.76-0.97)	0.02	0.95 (0.77-1.19)	0.68
C48:1 TAG	0.93 (0.83-1.04)	0.18	0.9 (0.77-1.05)	0.19	0.91 (0.79-1.04)	0.17	0.93 (0.79-1.08)	0.33	0.85 (0.75-0.96)	0.01	0.96 (0.77-1.19)	0.68
C48:2 TAG	0.94 (0.83-1.05)	0.25	0.89 (0.76-1.04)	0.16	0.91 (0.79-1.04)	0.16	0.94 (0.8-1.1)	0.44	0.85 (0.76-0.96)	0.01	1.04 (0.85-1.27)	0.69
C48:3 TAG	0.91 (0.82-1.02)	0.12	0.88 (0.75-1.03)	0.11	0.93 (0.81-1.07)	0.31	0.9 (0.77-1.05)	0.18	0.85 (0.75-0.96)	0.01	0.96 (0.77-1.19)	0.69
C49:2 TAG	0.97 (0.87-1.08)	0.55	0.95 (0.82-1.1)	0.47	0.92 (0.81-1.04)	0.19	0.99 (0.85-1.15)	0.89	0.89 (0.8-1)	0.06	0.96 (0.77-1.19)	0.69
C49:3 TAG	0.96 (0.86-1.08)	0.52	0.97 (0.83-1.13)	0.69	0.95 (0.84-1.08)	0.45	0.98 (0.84-1.15)	0.81	0.91 (0.81-1.02)	0.10	0.96 (0.76-1.2)	0.69
C50:0 TAG	0.92 (0.82-1.03)	0.14	0.89 (0.76-1.04)	0.15	0.88 (0.76-1.01)	0.06	0.94 (0.8-1.1)	0.43	0.85 (0.75-0.96)	0.01	0.96 (0.79-1.17)	0.69
C50:1 TAG	0.91 (0.81-1.03)	0.12	0.9 (0.76-1.07)	0.24	0.9 (0.78-1.04)	0.17	0.9 (0.77-1.06)	0.23	0.84 (0.74-0.95)	0.01	1.04 (0.85-1.27)	0.69
C50:2 TAG	0.9 (0.8-1.02)	0.10	0.88 (0.74-1.05)	0.15	0.9 (0.78-1.04)	0.15	0.92 (0.78-1.08)	0.29	0.84 (0.74-0.95)	0.01	1.04 (0.85-1.29)	0.70
C50:3 TAG	0.93 (0.83-1.04)	0.22	0.91 (0.77-1.07)	0.25	0.98 (0.85-1.13)	0.78	0.92 (0.78-1.08)	0.32	0.89 (0.79-1.01)	0.07	0.96 (0.79-1.18)	0.70
C50:4 TAG	0.91 (0.81-1.02)	0.10	0.91 (0.78-1.07)	0.26	1.01 (0.88-1.16)	0.84	0.86 (0.73-1)	0.05	0.89 (0.79-1)	0.06	1.04 (0.84-1.3)	0.70
C50:5 TAG	0.89 (0.79-0.99)	0.04	0.88 (0.75-1.03)	0.10	0.99 (0.86-1.13)	0.85	0.83 (0.71-0.97)	0.02	0.87 (0.77-0.98)	0.02	1.04 (0.84-1.3)	0.70
C50:6 TAG	0.9 (0.8-1)	0.06	0.89 (0.76-1.04)	0.15	0.97 (0.85-1.12)	0.71	0.85 (0.72-0.99)	0.03	0.88 (0.78-0.99)	0.03	0.96 (0.77-1.19)	0.71
C51:0 TAG	0.95 (0.85-1.07)	0.42	0.92 (0.79-1.07)	0.29	0.89 (0.78-1.02)	0.09	0.98 (0.84-1.15)	0.79	0.89 (0.79-1)	0.05	0.96 (0.78-1.18)	0.71
C51:1 TAG	0.94 (0.84-1.05)	0.25	0.93 (0.79-1.09)	0.37	0.9 (0.78-1.03)	0.11	0.95 (0.81-1.11)	0.51	0.85 (0.75-0.96)	0.01	0.96 (0.77-1.19)	0.72
C51:2 TAG	0.95 (0.85-1.06)	0.35	0.94 (0.81-1.09)	0.42	0.89 (0.78-1.01)	0.08	0.97 (0.83-1.13)	0.67	0.86 (0.77-0.97)	0.01	0.96 (0.78-1.18)	0.72
C51:3 TAG	0.96 (0.86-1.08)	0.50	1.01 (0.86-1.19)	0.89	1 (0.88-1.14)	0.99	0.97 (0.83-1.13)	0.67	0.93 (0.83-1.05)	0.25	1.04 (0.84-1.29)	0.72
C52:0 TAG	0.92 (0.82-1.04)	0.18	0.89 (0.76-1.04)	0.15	0.88 (0.77-1.01)	0.08	0.94 (0.8-1.11)	0.48	0.87 (0.77-0.98)	0.02	0.96 (0.79-1.18)	0.72
C52:1 TAG	0.9 (0.8-1.01)	0.07	0.89 (0.75-1.05)	0.17	0.88 (0.76-1.01)	0.07	0.88 (0.75-1.03)	0.11	0.83 (0.73-0.94)	0.00	0.96 (0.79-1.18)	0.72
C52:2 TAG	0.93 (0.83-1.05)	0.24	0.95 (0.8-1.12)	0.52	0.96 (0.84-1.11)	0.58	0.88 (0.75-1.04)	0.14	0.88 (0.78-0.99)	0.04	0.96 (0.77-1.19)	0.73
C52:3 TAG	0.95 (0.85-1.06)	0.38	0.99 (0.84-1.16)	0.86	1.03 (0.9-1.17)	0.69	0.87 (0.74-1.03)	0.10	0.93 (0.82-1.05)	0.23	0.96 (0.78-1.19)	0.73

C52:4 TAG	0.94 (0.84-1.05)	0.27	0.98 (0.84-1.14)	0.78	1.08 (0.95-1.22)	0.25	0.88 (0.75-1.03)	0.11	0.98 (0.87-1.1)	0.70	0.96 (0.78-1.19)	0.73
C52:5 TAG	0.94 (0.84-1.04)	0.24	0.98 (0.84-1.14)	0.75	1.08 (0.95-1.22)	0.26	0.88 (0.75-1.03)	0.10	0.99 (0.88-1.11)	0.84	0.96 (0.77-1.19)	0.73
C52:6 TAG	0.9 (0.8-1)	0.06	0.91 (0.78-1.07)	0.25	1.05 (0.92-1.19)	0.49	0.8 (0.68-0.93)	0.01	0.93 (0.82-1.04)	0.21	0.96 (0.78-1.19)	0.73
C52:7 TAG	0.9 (0.8-1)	0.05	0.91 (0.78-1.06)	0.22	1.01 (0.89-1.16)	0.84	0.81 (0.69-0.94)	0.01	0.91 (0.81-1.02)	0.11	1.04 (0.84-1.28)	0.74
C53:2 TAG	0.94 (0.84-1.06)	0.31	0.99 (0.85-1.16)	0.94	0.94 (0.82-1.07)	0.34	0.93 (0.79-1.09)	0.38	0.89 (0.79-1)	0.06	1.04 (0.84-1.28)	0.74
C53:3 TAG	0.96 (0.86-1.08)	0.52	1.03 (0.89-1.21)	0.67	1.02 (0.9-1.17)	0.74	0.95 (0.82-1.1)	0.51	0.96 (0.85-1.07)	0.46	0.96 (0.77-1.2)	0.74
C54:1 TAG	0.93 (0.83-1.04)	0.21	0.91 (0.78-1.06)	0.25	0.92 (0.8-1.06)	0.24	0.92 (0.78-1.09)	0.33	0.9 (0.79-1.01)	0.08	1.04 (0.84-1.28)	0.75
C54:2 TAG	0.93 (0.83-1.04)	0.23	0.92 (0.79-1.08)	0.32	0.96 (0.84-1.09)	0.50	0.9 (0.76-1.06)	0.22	0.91 (0.81-1.03)	0.14	0.96 (0.77-1.2)	0.75
C54:3 TAG	0.98 (0.88-1.09)	0.68	0.98 (0.85-1.14)	0.82	1.05 (0.93-1.19)	0.43	0.92 (0.78-1.08)	0.32	1.02 (0.91-1.14)	0.76	0.97 (0.78-1.19)	0.75
C54:4 TAG	0.98 (0.88-1.1)	0.78	0.99 (0.85-1.15)	0.85	1.08 (0.95-1.22)	0.23	0.94 (0.8-1.11)	0.46	1.05 (0.94-1.18)	0.41	1.03 (0.84-1.27)	0.76
C54:5 TAG	0.95 (0.84-1.06)	0.34	0.91 (0.77-1.07)	0.24	0.96 (0.84-1.1)	0.56	0.87 (0.75-1.02)	0.10	0.89 (0.79-1.01)	0.06	0.97 (0.78-1.2)	0.77
C54:6 TAG	0.95 (0.85-1.05)	0.32	0.97 (0.84-1.13)	0.73	1.06 (0.94-1.21)	0.33	0.9 (0.77-1.05)	0.17	1.02 (0.91-1.14)	0.74	0.97 (0.79-1.19)	0.77
C54:7 TAG	0.91 (0.82-1.01)	0.08	0.94 (0.81-1.08)	0.37	1.05 (0.93-1.19)	0.40	0.81 (0.69-0.95)	0.01	0.97 (0.87-1.09)	0.62	1.03 (0.84-1.26)	0.77
C54:8 TAG	0.9 (0.81-1)	0.05	0.93 (0.8-1.07)	0.30	1.03 (0.91-1.17)	0.63	0.8 (0.68-0.94)	0.01	0.93 (0.83-1.05)	0.25	0.97 (0.78-1.2)	0.77
C54:9 TAG	0.93 (0.83-1.04)	0.20	0.96 (0.82-1.11)	0.57	1.03 (0.91-1.17)	0.63	0.83 (0.7-0.97)	0.02	0.95 (0.85-1.07)	0.41	1.03 (0.84-1.27)	0.77
C55:2 TAG	0.95 (0.85-1.06)	0.36	0.95 (0.81-1.11)	0.53	0.93 (0.81-1.06)	0.29	0.95 (0.81-1.11)	0.50	0.9 (0.79-1.01)	0.07	1.03 (0.84-1.27)	0.78
C55:3 TAG	0.99 (0.89-1.11)	0.92	1.02 (0.88-1.19)	0.79	1 (0.88-1.14)	0.95	1.04 (0.9-1.2)	0.60	1.02 (0.91-1.14)	0.71	1.03 (0.83-1.28)	0.78
C56:1 TAG	0.94 (0.84-1.05)	0.28	0.92 (0.79-1.08)	0.31	0.96 (0.84-1.1)	0.54	0.92 (0.78-1.08)	0.31	0.93 (0.82-1.04)	0.21	0.97 (0.78-1.2)	0.78
C56:10 TAG	0.95 (0.85-1.06)	0.34	0.98 (0.84-1.14)	0.82	1.05 (0.92-1.19)	0.50	0.83 (0.7-0.97)	0.02	0.98 (0.87-1.1)	0.70	1.03 (0.83-1.29)	0.79
C56:2 TAG	0.96 (0.86-1.07)	0.47	0.98 (0.84-1.14)	0.75	0.98 (0.85-1.12)	0.73	0.93 (0.79-1.09)	0.36	0.94 (0.84-1.06)	0.34	0.97 (0.78-1.21)	0.79
C56:3 TAG	0.97 (0.87-1.08)	0.61	0.98 (0.84-1.14)	0.76	1.02 (0.9-1.16)	0.75	0.94 (0.8-1.1)	0.43	0.98 (0.87-1.1)	0.73	0.97 (0.77-1.22)	0.79
C56:4 TAG	1.01 (0.91-1.12)	0.88	1.03 (0.89-1.2)	0.68	1.09 (0.96-1.23)	0.17	0.97 (0.83-1.13)	0.71	1.04 (0.93-1.16)	0.53	0.97 (0.8-1.19)	0.80
C56:5 TAG	1.1 (0.98-1.23)	0.09	1.15 (0.99-1.34)	0.06	1.13 (0.99-1.28)	0.07	1.01 (0.87-1.17)	0.89	1.09 (0.97-1.22)	0.15	0.97 (0.78-1.21)	0.81
C56:6 TAG	1.04 (0.93-1.16)	0.50	1.05 (0.91-1.22)	0.52	1.1 (0.96-1.25)	0.16	0.94 (0.8-1.09)	0.40	1.06 (0.94-1.19)	0.34	0.97 (0.79-1.21)	0.81
C56:7 TAG	1 (0.89-1.11)	0.94	1.03 (0.88-1.19)	0.74	1.13 (0.99-1.28)	0.07	0.91 (0.78-1.07)	0.26	1.06 (0.95-1.19)	0.32	1.02 (0.83-1.26)	0.82
C56:8 TAG	1.01 (0.91-1.13)	0.80	1.06 (0.92-1.23)	0.40	1.14 (1-1.29)	0.05	0.94 (0.8-1.09)	0.40	1.09 (0.98-1.23)	0.13	0.98 (0.79-1.2)	0.82
C56:9 TAG	0.96 (0.86-1.07)	0.49	1 (0.86-1.16)	1.00	1.07 (0.94-1.22)	0.28	0.85 (0.72-1)	0.05	1.01 (0.9-1.14)	0.81	1.02 (0.83-1.26)	0.82
C58:11 TAG	0.97 (0.87-1.09)	0.64	1.01 (0.87-1.18)	0.90	1.04 (0.91-1.19)	0.56	0.86 (0.73-1.02)	0.08	1.01 (0.89-1.13)	0.92	0.97 (0.78-1.22)	0.83
C58:6 TAG	1.04 (0.93-1.17)	0.47	1.05 (0.9-1.23)	0.52	1.09 (0.95-1.24)	0.21	1 (0.85-1.16)	0.96	1.07 (0.95-1.2)	0.26	0.98 (0.79-1.21)	0.83
C58:7 TAG	1.07 (0.96-1.2)	0.21	1.11 (0.96-1.29)	0.16	1.13 (0.99-1.29)	0.07	1.04 (0.9-1.21)	0.56	1.14 (1.02-1.28)	0.03	1.02 (0.83-1.26)	0.84
C58:9 TAG	1.05 (0.94-1.18)	0.37	1.09 (0.94-1.27)	0.27	1.12 (0.97-1.28)	0.12	0.99 (0.85-1.16)	0.93	1.13 (1.01-1.27)	0.04	1.02 (0.83-1.25)	0.84
C60:12 TAG	1.05 (0.94-1.18)	0.37	1.12 (0.96-1.31)	0.16	1.07 (0.94-1.23)	0.30	1.01 (0.86-1.19)	0.89	1.1 (0.98-1.24)	0.12	0.98 (0.78-1.22)	0.85

campesterol	0.95 (0.85-1.06)	0.34	1 (0.86-1.17)	0.97	0.92 (0.81-1.05)	0.23	0.95 (0.81-1.1)	0.48	0.91 (0.81-1.03)	0.14	0.98 (0.79-1.21)	0.85
chenodeoxycholate/deoxycholate	0.95 (0.86-1.06)	0.36	1 (0.86-1.16)	0.99	1.02 (0.9-1.16)	0.78	0.88 (0.75-1.02)	0.09	0.91 (0.81-1.02)	0.09	0.98 (0.79-1.21)	0.85
cholesterol	0.95 (0.85-1.06)	0.35	1 (0.85-1.17)	0.96	0.94 (0.82-1.08)	0.37	0.93 (0.79-1.08)	0.33	0.92 (0.81-1.03)	0.16	0.98 (0.78-1.24)	0.86
CMPF	0.99 (0.88-1.11)	0.84	1.05 (0.9-1.23)	0.52	1.06 (0.93-1.21)	0.38	0.95 (0.81-1.11)	0.52	1 (0.88-1.12)	0.96	0.98 (0.8-1.2)	0.86
coenzyme Q10	0.99 (0.89-1.11)	0.90	1.11 (0.95-1.29)	0.19	1.01 (0.88-1.15)	0.90	0.89 (0.77-1.03)	0.13	0.96 (0.85-1.07)	0.45	0.98 (0.79-1.22)	0.87
erythronate/threonate	1.04 (0.93-1.15)	0.52	1.08 (0.93-1.25)	0.31	1.05 (0.93-1.2)	0.42	0.98 (0.84-1.14)	0.78	1.04 (0.93-1.17)	0.49	0.98 (0.79-1.22)	0.87
fucose	1.01 (0.91-1.13)	0.80	1.02 (0.88-1.17)	0.83	1.06 (0.93-1.21)	0.40	0.96 (0.83-1.11)	0.56	0.96 (0.85-1.07)	0.46	0.98 (0.81-1.2)	0.88
gentisate	0.96 (0.85-1.1)	0.58	1.07 (0.9-1.27)	0.45	1.02 (0.88-1.18)	0.84	0.99 (0.83-1.18)	0.90	1.01 (0.88-1.16)	0.87	1.02 (0.82-1.26)	0.88
glutamate	1 (0.89-1.12)	0.99	0.96 (0.83-1.12)	0.63	0.94 (0.83-1.07)	0.34	1.14 (0.96-1.36)	0.13	0.95 (0.84-1.08)	0.44	1.02 (0.82-1.25)	0.88
glycerate	1.03 (0.93-1.15)	0.54	1.05 (0.9-1.23)	0.51	0.98 (0.87-1.11)	0.74	1.12 (0.96-1.31)	0.16	1.04 (0.93-1.17)	0.51	0.98 (0.79-1.22)	0.89
hexose monophosphate	1.02 (0.91-1.13)	0.75	0.99 (0.86-1.15)	0.92	0.95 (0.84-1.08)	0.45	1.11 (0.95-1.3)	0.20	1.02 (0.9-1.14)	0.78	1.02 (0.82-1.26)	0.89
homovanillate	1.06 (0.95-1.19)	0.33	1.03 (0.88-1.21)	0.68	1.04 (0.92-1.19)	0.51	1.02 (0.86-1.21)	0.84	1.03 (0.92-1.16)	0.62	0.98 (0.79-1.23)	0.89
indole-3-propionate	1.03 (0.92-1.16)	0.56	1.05 (0.9-1.23)	0.53	1.04 (0.91-1.19)	0.55	1.09 (0.93-1.28)	0.27	1.09 (0.96-1.22)	0.17	1.02 (0.82-1.26)	0.89
indoleacetate	0.97 (0.87-1.08)	0.61	0.97 (0.83-1.13)	0.68	0.98 (0.86-1.11)	0.72	0.88 (0.76-1.02)	0.08	0.98 (0.88-1.11)	0.79	1.02 (0.81-1.27)	0.89
indoxylsulfate	0.88 (0.79-0.99)	0.03	0.88 (0.76-1.02)	0.09	0.93 (0.82-1.06)	0.28	0.85 (0.74-0.99)	0.03	0.9 (0.8-1)	0.06	0.99 (0.81-1.21)	0.91
kynurenine	0.93 (0.83-1.03)	0.17	0.94 (0.81-1.09)	0.41	0.95 (0.84-1.09)	0.48	0.88 (0.75-1.03)	0.12	0.95 (0.85-1.07)	0.43	1.01 (0.82-1.25)	0.91
malonate	0.96 (0.86-1.06)	0.41	1 (0.86-1.15)	0.97	0.93 (0.81-1.05)	0.25	1.01 (0.87-1.17)	0.90	0.93 (0.84-1.05)	0.24	0.99 (0.8-1.23)	0.93
MDA	1.06 (0.95-1.18)	0.32	1.07 (0.93-1.25)	0.34	1.04 (0.91-1.18)	0.58	1.01 (0.87-1.18)	0.86	1.03 (0.92-1.15)	0.62	1.01 (0.83-1.23)	0.93
mesaconate	1.06 (0.95-1.19)	0.27	1.02 (0.88-1.19)	0.77	0.99 (0.87-1.12)	0.83	1.16 (1-1.35)	0.06	1.08 (0.96-1.21)	0.21	0.99 (0.8-1.22)	0.93
N-acetylglutamate	1.09 (0.97-1.22)	0.17	1.09 (0.93-1.27)	0.29	0.96 (0.84-1.1)	0.57	1.23 (1.04-1.46)	0.02	1.06 (0.94-1.2)	0.34	0.99 (0.8-1.22)	0.94
palmitoylethanolamide	1.08 (0.97-1.21)	0.14	1.19 (1.03-1.39)	0.02	1.1 (0.97-1.25)	0.12	0.94 (0.8-1.11)	0.47	1.02 (0.91-1.15)	0.72	1.01 (0.8-1.26)	0.94
pentose monophosphate	0.99 (0.89-1.11)	0.91	0.96 (0.82-1.11)	0.56	0.99 (0.87-1.13)	0.87	1.07 (0.91-1.26)	0.43	1.09 (0.96-1.22)	0.17	0.99 (0.81-1.21)	0.95
phenyllactate	1.06 (0.95-1.18)	0.34	1.01 (0.88-1.18)	0.85	1 (0.88-1.14)	0.97	1.04 (0.89-1.21)	0.60	1.09 (0.98-1.23)	0.13	0.99 (0.8-1.23)	0.96
quinolinate	0.9 (0.81-1.01)	0.06	0.87 (0.74-1.02)	0.08	0.96 (0.84-1.09)	0.50	0.94 (0.81-1.1)	0.43	0.94 (0.83-1.05)	0.26	1 (0.81-1.23)	0.97
suberate	0.97 (0.87-1.08)	0.62	0.94 (0.81-1.08)	0.38	0.95 (0.83-1.07)	0.40	1.04 (0.89-1.2)	0.65	1.03 (0.92-1.16)	0.57	1 (0.81-1.25)	0.97
taurodeoxycholate/taurochenodeoxycholate	1.13 (1-1.27)	0.05	1.05 (0.89-1.23)	0.57	1.04 (0.91-1.2)	0.58	1.24 (1.05-1.48)	0.01	1.14 (1.01-1.3)	0.04	1 (0.81-1.24)	0.98
threitol	1.04 (0.93-1.16)	0.53	0.98 (0.84-1.14)	0.81	1.07 (0.94-1.22)	0.31	1.02 (0.87-1.2)	0.79	1.05 (0.93-1.18)	0.44	1 (0.82-1.23)	0.99
thymine	1.02 (0.91-1.14)	0.75	1.01 (0.87-1.18)	0.89	0.95 (0.84-1.08)	0.44	1.13 (0.97-1.31)	0.11	1.08 (0.96-1.21)	0.19	1 (0.81-1.24)	0.99
uracil	0.99 (0.88-1.11)	0.88	1 (0.86-1.17)	0.96	1 (0.88-1.15)	0.95	1 (0.85-1.17)	0.96	1.01 (0.89-1.14)	0.90	1 (0.81-1.24)	0.99
xanthurenate	0.92 (0.82-1.03)	0.13	0.94 (0.81-1.09)	0.41	0.95 (0.84-1.08)	0.46	0.92 (0.78-1.07)	0.29	0.96 (0.85-1.08)	0.48	1 (0.8-1.25)	1.00

Model adjusted for BMI at age 18, weight change (from age 18 to time of first blood draw), age at menarche, parity and age at first birth, breastfeeding history, family history of breast cancer in a first degree relative, personal history of benign breast disease, physical activity, alcohol intake (by quintile), and oral contraceptive use at blood collection.
