# Supplementary material for: Prospective study of circulating metabolomic profiles and breast cancer incidence among predominantly premenopausal women
Source: Br J Cancer. Author manuscript; Available in PMC 2025 Dec 6. (PMC12572396; doi:10.1038/s41416-025-03159-2)
Supplement: Suppl Table 5 [file NIHMS2109610-supplement-Suppl_Table_5.pdf]

Supplemental Table 5. Metabolite set enrichment analysis (MSEA) by class of metabolites (n=218) for breast cancer incidence stratified by subgroups, Nurses' Health Study II (1996-2011).

	Premenopausal at blood collection			Postmenopausal at blood collection		
**Pathway**	**pval.m3**	**padj.m3**	**NES.m3**	**pval.m3**	**padj.m3**	**NES.m3**
**Amino acids, peptides, and analogues**	0.2188	0.2917	1.2788	0.2815	0.4288	1.1254
**Carbohydrates and carbohydrate conjugates**	0.1004	0.1461	1.4803	0.8557	0.8557	-0.7234
**Ceramides**	0.8079	0.8617	0.6867	0.1769	0.4288	-1.2331
**Cholesteryl esters**	0.0855	0.1368	1.4355	0.2567	0.4288	-1.1680
**Diglycerides**	0.0461	0.1230	-1.5362	0.3484	0.4288	1.0633
**Fatty acids and conjugates**	0.0795	0.1368	1.4896	0.1618	0.4288	-1.2736
**Lysophosphatidylcholines**	0.9470	0.9470	0.6009	0.3220	0.4288	1.0993
**Lysophosphatidylethanolamines**	0.2564	0.3156	1.1722	0.2471	0.4288	1.1537
**Steroids**	0.0637	0.1297	1.4954	0.1093	0.4288	-1.3458
**Phosphatidylcholine plasmalogens**	0.0000	0.0000	2.5863	0.1241	0.4288	-1.3240
**Phosphatidylcholines**	0.0109	0.0435	-1.7424	0.7255	0.7739	-0.8155
**Phosphatidylethanolamine plasmalogens**	0.0002	0.0012	2.2708	0.2668	0.4288	-1.1560
**Phosphatidylethanolamines**	0.4643	0.5306	-0.9913	0.3338	0.4288	-1.1164
**Sphingomyelins**	0.0648	0.1297	1.5856	0.6897	0.7739	-0.8498
**Triglycerides (<3 DB)**	0.0000	0.0000	-2.3807	0.0000	0.0000	-2.4767
**Triglycerides (≧3 DB)**	0.0160	0.0511	-1.5466	0.0000	0.0000	3.0682

	Normal BMI at blood collection			High BMI at blood collection		
**Pathway**	**pval.m3**	**padj.m3**	**NES.m3**	**pval.m3**	**padj.m3**	**NES.m3**
**Amino acids, peptides, and analogues**	0.2265	0.4027	-1.2130	0.0002	0.0011	2.0426
**Carbohydrates and carbohydrate conjugates**	0.0418	0.1224	1.6079	0.4536	0.6048	1.0057
**Ceramides**	0.3557	0.5452	-1.1082	0.7632	0.8722	-0.8004
**Cholesteryl esters**	0.2003	0.4006	-1.2700	0.0459	0.0816	1.5068
**Diglycerides**	0.0187	0.0996	1.7236	0.0009	0.0036	-1.9062
**Fatty acids and conjugates**	0.8628	0.9861	-0.6848	0.0727	0.1120	1.5207
**Lysophosphatidylcholines**	0.9969	0.9969	0.5027	0.9877	0.9877	-0.5169
**Lysophosphatidylethanolamines**	0.9937	0.9969	-0.4816	0.0770	0.1120	1.5544
**Steroids**	0.4795	0.6394	1.0221	0.9067	0.9672	-0.6762
**Phosphatidylcholine plasmalogens**	0.1982	0.4006	1.2553	0.0025	0.0081	2.1036
**Phosphatidylcholines**	0.0438	0.1224	-1.5260	0.0186	0.0373	-1.5928
**Phosphatidylethanolamine plasmalogens**	0.0459	0.1224	1.6241	0.0002	0.0011	2.2644
**Phosphatidylethanolamines**	0.6873	0.8459	-0.8213	0.6744	0.8301	0.8669
**Sphingomyelins**	0.3748	0.5452	-1.0862	0.0052	0.0138	1.9460
**Triglycerides (<3 DB)**	0.0000	0.0000	-2.6053	0.0125	0.0287	-1.6139
**Triglycerides (≧3 DB)**	0.0000	0.0000	2.5814	0.0000	0.0000	-2.3573

	ER-positive			ER-negtive		
**Pathway**	**pval.m3**	**padj.m3**	**NES.m3**	**pval.m3**	**padj.m3**	**NES.m3**
**Amino acids, peptides, and analogues**	0.7158	0.8180	0.8057	0.0035	0.0556	1.9514
**Carbohydrates and carbohydrate conjugates**	0.2105	0.3368	1.2132	0.0087	0.0697	1.8589
**Ceramides**	0.3472	0.4629	-1.0925	0.1281	0.2277	-1.2885
**Cholesteryl esters**	0.0148	0.0472	1.7273	0.0603	0.1609	-1.4616
**Diglycerides**	0.1097	0.2195	-1.3163	0.1899	0.3039	1.2410
**Fatty acids and conjugates**	0.0011	0.0086	2.0975	0.5638	0.6189	0.9106
**Lysophosphatidylcholines**	0.9284	0.9890	-0.6302	0.2482	0.3610	1.1909
**Lysophosphatidylethanolamines**	0.1872	0.3329	1.1982	0.3595	0.4424	1.0524
**Steroids**	0.6880	0.8180	0.8153	0.0293	0.1173	-1.5614
**Phosphatidylcholine plasmalogens**	0.0094	0.0377	1.8899	0.0919	0.2101	-1.3793
**Phosphatidylcholines**	0.0264	0.0705	-1.5349	0.5802	0.6189	-0.9324
**Phosphatidylethanolamine plasmalogens**	0.0031	0.0164	1.9508	0.0216	0.1150	-1.5876
**Phosphatidylethanolamines**	0.0889	0.2032	-1.3414	0.3555	0.4424	1.0422
**Sphingomyelins**	0.3021	0.4395	1.1126	0.0404	0.1293	-1.5670
**Triglycerides (<3 DB)**	0.0000	0.0000	-2.3774	0.1218	0.2277	-1.3125
**Triglycerides (≧3 DB)**	0.9890	0.9890	-0.5454	0.6942	0.6942	-0.8567

	Premenopausal at dx			Postmenopausal at dx		
**Pathway**	**pval.m3**	**padj.m3**	**NES.m3**	**pval.m3**	**padj.m3**	**NES.m3**
**Amino acids, peptides, and analogues**	0.3941	0.4952	1.0579	0.8766	0.8770	0.6890
**Carbohydrates and carbohydrate conjugates**	0.3795	0.4952	1.0881	0.6298	0.8770	0.8697
**Ceramides**	0.2244	0.4952	1.2137	0.3563	0.7126	-1.0829
**Cholesteryl esters**	0.1746	0.4655	1.2584	0.5093	0.8770	-1.0022
**Diglycerides**	0.0020	0.0106	-1.8998	0.7872	0.8770	0.7865
**Fatty acids and conjugates**	0.8975	0.9078	0.6664	0.8417	0.8770	-0.7418
**Lysophosphatidylcholines**	0.3126	0.4952	-1.1091	0.7547	0.8770	-0.8095
**Lysophosphatidylethanolamines**	0.4023	0.4952	-1.0384	0.0271	0.0868	1.6991
**Steroids**	0.0001	0.0005	2.1889	0.3099	0.7082	1.0799
**Phosphatidylcholine plasmalogens**	0.9078	0.9078	0.6637	0.0009	0.0075	-1.7848
**Phosphatidylcholines**	0.0100	0.0321	1.7120	0.0003	0.0055	2.3523
**Phosphatidylethanolamine plasmalogens**	0.3341	0.4952	1.1089	0.1012	0.2698	-1.3198
**Phosphatidylethanolamines**	0.0033	0.0133	1.8746	0.0055	0.0295	-1.6953
**Sphingomyelins**	0.3761	0.4952	1.0960	0.8030	0.8770	-0.7913
**Triglycerides (<3 DB)**	0.0000	0.0000	-2.8303	0.0078	0.0314	-1.6122
**Triglycerides (≧3 DB)**	0.4459	0.5096	-0.9946	0.8770	0.8770	-0.7205
