# Supplementary material for: Prospective study of circulating metabolomic profiles and breast cancer incidence among predominantly premenopausal women
Source: Br J Cancer. Author manuscript; Available in PMC 2025 Dec 6. (PMC12572396; doi:10.1038/s41416-025-03159-2)
Supplement: Suppl Table 4 [file NIHMS2109610-supplement-Suppl_Table_4.pdf]

Supplemental Table 4. Odds ratios and 95% confidence intervals for associations between individual metabolites (per 1 SD increase in metabolite level) and breast cancer incidence, stratified by median time since blood collection, Nurses' Health Study (1996-2011).

		≤ 6.5 years			> 6.5 years	
HMDB_ID	METABOLITE	OR.CI	p.value		OR.CI	p.value
HMDB0000510	2-aminoadipate	0.95 (0.85-1.07)	0.382		0.92 (0.8-1.05)	0.209
HMDB0000650	2-aminobutyrate	1.07 (0.95-1.2)	0.253		1.08 (0.95-1.23)	0.26
HMDB0094649	2-aminoheptanoate	1.01 (0.9-1.13)	0.918		1.04 (0.91-1.19)	0.558
HMDB0000407	2-hydroxy-3-methylbutyrate	0.94 (0.84-1.05)	0.288		0.97 (0.85-1.11)	0.699
HMDB0000317*	2-hydroxy-3-methylpentanoate/hydroxyisocaproate	1.05 (0.94-1.18)	0.397		1.04 (0.91-1.18)	0.595
HMDB0000694	2-hydroxyglutarate	1.13 (1.01-1.26)	0.038		0.97 (0.85-1.11)	0.622
HMDB0000355	3-hydroxymethylglutarate/anhydroDglucose	1 (0.89-1.12)	0.963		1.04 (0.91-1.19)	0.558
HMDB0000555*	3-methyladipate/pimelate	1.05 (0.94-1.17)	0.379		0.92 (0.8-1.04)	0.185
HMDB0000017	4-pyridoxate	0.95 (0.85-1.07)	0.381		0.96 (0.84-1.1)	0.601
HMDB0000448*	adipate/methylglutarate	1.01 (0.91-1.13)	0.816		0.99 (0.87-1.12)	0.829
HMDB0000508*	adonitol/arabitol	1.03 (0.92-1.16)	0.598		0.95 (0.83-1.1)	0.505
HMDB0000008*	alpha-hydroxybutyrate/beta-hydroxybutyrate/hydroxyisobutyrate	1.02 (0.91-1.15)	0.685		1.08 (0.94-1.24)	0.269
HMDB0000491*	alpha-keto-beta-methylvalerate/alpha-ketoisocaproate	1.12 (1-1.26)	0.046		1.05 (0.92-1.2)	0.469
HMDB0000019	alpha-ketoisovalerate	1.09 (0.98-1.22)	0.128		1.08 (0.95-1.24)	0.251
HMDB0000191	aspartate	0.99 (0.89-1.11)	0.904		1.08 (0.95-1.24)	0.24
HMDB0006725	C14:0 CE	0.92 (0.82-1.03)	0.139		0.94 (0.82-1.07)	0.332
HMDB0010379	C14:0 LPC	0.95 (0.85-1.07)	0.413		0.95 (0.83-1.09)	0.456
HMDB0012097	C14:0 SM	1 (0.89-1.13)	0.985		0.96 (0.83-1.1)	0.518
HMDB0000885	C16:0 CE	0.95 (0.85-1.07)	0.422		1.14 (1-1.31)	0.05
HMDB0004949	C16:0 Ceramide (d18:1)	1.03 (0.92-1.15)	0.661		0.95 (0.83-1.08)	0.433
HMDB0010382	C16:0 LPC	0.97 (0.87-1.08)	0.58		0.95 (0.84-1.08)	0.474
HMDB0011503	C16:0 LPE	1 (0.89-1.12)	0.959		0.93 (0.81-1.06)	0.288
HMDB0010169	C16:0 SM	1.05 (0.94-1.17)	0.4		1.03 (0.9-1.17)	0.678
HMDB0000658*	C16:1 CE	0.9 (0.8-1.01)	0.07		0.85 (0.74-0.97)	0.02
HMDB0010383*	C16:1 LPC	0.96 (0.86-1.07)	0.476		0.9 (0.79-1.02)	0.099
HMDB0010368	C18:0 CE	1.02 (0.91-1.14)	0.702		1.06 (0.93-1.21)	0.405
HMDB0010384	C18:0 LPC	1.03 (0.92-1.15)	0.645		1.03 (0.9-1.17)	0.672
HMDB0011130	C18:0 LPE	1.02 (0.91-1.14)	0.745		0.96 (0.84-1.09)	0.546
HMDB0001348	C18:0 SM	1.01 (0.9-1.13)	0.844		1.06 (0.92-1.21)	0.431
HMDB0000918*	C18:1 CE	0.97 (0.87-1.09)	0.653		1.08 (0.95-1.24)	0.257
HMDB0002815*	C18:1 LPC	1 (0.89-1.13)	0.962		0.97 (0.85-1.11)	0.663
HMDB0011506*	C18:1 LPE	1.03 (0.91-1.15)	0.677		0.92 (0.81-1.06)	0.261
HMDB0012101*	C18:1 SM	1.02 (0.91-1.14)	0.744		1.03 (0.9-1.18)	0.628
HMDB0000610*	C18:2 CE	0.97 (0.86-1.09)	0.579		1.13 (0.98-1.3)	0.084

HMDB0010386*	C18:2 LPC	1.01 (0.89-1.14)	0.855		0.98 (0.85-1.13)	0.828
HMDB0011507*	C18:2 LPE	1.01 (0.9-1.14)	0.832		0.92 (0.81-1.06)	0.252
HMDB0010370*	C18:3 CE	0.93 (0.83-1.04)	0.207		0.92 (0.81-1.05)	0.238
HMDB0010387*	C18:3 LPC	0.99 (0.88-1.1)	0.813		0.96 (0.84-1.09)	0.514
HMDB0012102	C20:0 SM	1.02 (0.9-1.14)	0.797		1.01 (0.88-1.16)	0.86
HMDB0006736*	C20:3 CE	0.96 (0.86-1.08)	0.525		1.07 (0.94-1.21)	0.322
HMDB0006726	C20:4 CE	1.04 (0.92-1.16)	0.545		1.09 (0.96-1.25)	0.177
HMDB0010395	C20:4 LPC	1.03 (0.92-1.15)	0.627		0.98 (0.86-1.12)	0.792
HMDB0011517	C20:4 LPE	1.1 (0.99-1.24)	0.084		0.91 (0.79-1.04)	0.148
HMDB0006731	C20:5 CE	0.95 (0.85-1.07)	0.412		0.99 (0.87-1.13)	0.907
HMDB0010397	C20:5 LPC	1 (0.89-1.13)	0.947		0.97 (0.85-1.12)	0.717
HMDB0004952	C22:0 Ceramide (d18:1)	1 (0.89-1.12)	0.947		0.89 (0.78-1.02)	0.103
HMDB0011520	C22:0 LPE	1.01 (0.9-1.14)	0.819		1.07 (0.93-1.22)	0.368
HMDB0012104*	C22:1 SM	1.03 (0.92-1.15)	0.626		0.98 (0.85-1.11)	0.718
HMDB0010375*	C22:5 CE	0.96 (0.85-1.09)	0.535		1.04 (0.9-1.19)	0.601
HMDB0006733	C22:6 CE	1.03 (0.91-1.16)	0.618		1.22 (1.07-1.4)	0.004
HMDB0010404	C22:6 LPC	1 (0.89-1.12)	0.992		1.07 (0.94-1.23)	0.302
HMDB0011526	C22:6 LPE	1.05 (0.93-1.18)	0.429		1.01 (0.88-1.15)	0.94
HMDB0004956	C24:0 Ceramide (d18:1)	1.02 (0.91-1.14)	0.706		0.91 (0.8-1.04)	0.167
HMDB0004953*	C24:1 Ceramide (d18:1)	1.02 (0.91-1.14)	0.7		0.92 (0.81-1.05)	0.218
HMDB0012107*	C24:1 SM	1.03 (0.91-1.16)	0.652		1.03 (0.9-1.18)	0.653
HMDB0007869*	C30:0 PC	0.96 (0.86-1.08)	0.488		0.93 (0.81-1.07)	0.303
HMDB0007870*	C30:1 PC	0.96 (0.85-1.08)	0.478		0.88 (0.77-1.01)	0.07
HMDB0007098*	C32:0 DAG	0.95 (0.84-1.07)	0.371		0.9 (0.78-1.03)	0.138
HMDB0007871*	C32:0 PC	1.01 (0.89-1.13)	0.915		0.93 (0.81-1.07)	0.297
HMDB0008923*	C32:0 PE	0.97 (0.87-1.09)	0.623		0.97 (0.85-1.11)	0.645
HMDB0007099*	C32:1 DAG	0.97 (0.86-1.09)	0.623		0.94 (0.82-1.08)	0.392
HMDB0007873*	C32:1 PC	0.94 (0.83-1.06)	0.3		0.82 (0.71-0.95)	0.007
HMDB0007874*	C32:2 PC	0.98 (0.87-1.1)	0.757		0.93 (0.81-1.07)	0.326
HMDB0007100*	C34:0 DAG	0.97 (0.86-1.09)	0.621		0.91 (0.79-1.04)	0.15
HMDB0008925*	C34:0 PE	0.98 (0.87-1.1)	0.703		0.97 (0.85-1.1)	0.621
HMDB0012356*	C34:0 PS	0.9 (0.8-1.01)	0.072		0.88 (0.76-1.01)	0.06
HMDB0007102*	C34:1 DAG	0.96 (0.85-1.08)	0.464		0.93 (0.81-1.07)	0.3
HMDB0007972*	C34:1 PC	0.93 (0.82-1.04)	0.213		0.8 (0.69-0.92)	0.002
HMDB0011208*	C34:1 PC plasmalogen	1.08 (0.97-1.21)	0.156		1.06 (0.93-1.2)	0.409
HMDB0011239*	C34:1 PC plasmalogen-B	1.05 (0.94-1.18)	0.362		1.1 (0.96-1.25)	0.169
HMDB0007103*	C34:2 DAG	0.97 (0.86-1.09)	0.637		0.96 (0.83-1.1)	0.52
HMDB0007973*	C34:2 PC	0.98 (0.87-1.1)	0.732		0.93 (0.81-1.06)	0.279
HMDB0011210*	C34:2 PC plasmalogen	1.04 (0.92-1.17)	0.529		1.06 (0.92-1.21)	0.433

HMDB0008928*	C34:2 PE	1.01 (0.9-1.14)	0.891		0.91 (0.79-1.04)	0.174
HMDB0008952*	C34:2 PE plasmalogen	1.03 (0.92-1.15)	0.632		1.04 (0.91-1.19)	0.532
HMDB0007132*	C34:3 DAG	1.01 (0.9-1.14)	0.87		0.99 (0.86-1.13)	0.832
HMDB0008006*	C34:3 PC	0.94 (0.83-1.05)	0.279		0.82 (0.72-0.94)	0.004
HMDB0011211*	C34:3 PC plasmalogen	1.03 (0.92-1.15)	0.638		1.08 (0.95-1.24)	0.232
HMDB0011343*	C34:3 PE plasmalogen	1.01 (0.91-1.13)	0.803		1.05 (0.92-1.19)	0.501
HMDB0007883*	C34:4 PC	0.96 (0.85-1.08)	0.499		0.9 (0.78-1.04)	0.143
HMDB0011214*	C34:5 PC plasmalogen	1.04 (0.93-1.16)	0.457		1.06 (0.93-1.2)	0.364
HMDB0008991*	C36:0 PE	0.94 (0.84-1.06)	0.325		0.99 (0.87-1.12)	0.847
HMDB0007216*	C36:1 DAG	0.95 (0.84-1.07)	0.375		0.88 (0.76-1.01)	0.066
HMDB0008038*	C36:1 PC	0.95 (0.85-1.06)	0.382		0.86 (0.76-0.98)	0.026
HMDB0008993*	C36:1 PE	0.99 (0.88-1.12)	0.875		0.92 (0.8-1.06)	0.231
HMDB0009016*	C36:1 PE plasmalogen	0.99 (0.88-1.11)	0.897		1.08 (0.94-1.24)	0.257
HMDB0007218*	C36:2 DAG	1.02 (0.91-1.15)	0.727		0.9 (0.78-1.03)	0.114
HMDB0008039*	C36:2 PC	1 (0.89-1.11)	0.977		0.97 (0.85-1.1)	0.623
HMDB0011243*	C36:2 PC plasmalogen	1.09 (0.97-1.22)	0.13		1.08 (0.95-1.24)	0.231
HMDB0008994*	C36:2 PE	0.98 (0.87-1.1)	0.75		0.91 (0.8-1.05)	0.209
HMDB0009082*	C36:2 PE plasmalogen	1.02 (0.92-1.14)	0.698		1.04 (0.91-1.18)	0.572
HMDB0007219*	C36:3 DAG	1.05 (0.94-1.18)	0.398		0.98 (0.86-1.11)	0.733
HMDB0008105*	C36:3 PC	0.99 (0.89-1.11)	0.899		0.91 (0.8-1.04)	0.168
HMDB0011244*	C36:3 PC plasmalogen	1.05 (0.93-1.17)	0.445		1.09 (0.95-1.24)	0.211
HMDB0009060*	C36:3 PE	1.03 (0.91-1.15)	0.675		0.94 (0.82-1.08)	0.374
HMDB0011441*	C36:3 PE plasmalogen	1.01 (0.91-1.13)	0.835		1.01 (0.89-1.15)	0.852
HMDB0007248*	C36:4 DAG	1.04 (0.93-1.16)	0.511		1.05 (0.92-1.19)	0.507
HMDB0011310*	C36:4 PC plasmalogen	1.06 (0.95-1.19)	0.3		1.03 (0.91-1.18)	0.614
HMDB0007983*	C36:4 PC-A	0.96 (0.86-1.07)	0.455		0.93 (0.82-1.06)	0.282
HMDB0008138*	C36:4 PC-B	1.02 (0.91-1.15)	0.731		0.95 (0.83-1.09)	0.457
HMDB0008937*	C36:4 PE	1.03 (0.91-1.16)	0.635		0.92 (0.8-1.06)	0.231
HMDB0011442*	C36:4 PE plasmalogen	1.01 (0.9-1.12)	0.917		1.03 (0.91-1.17)	0.627
HMDB0011221*	C36:5 PC plasmalogen-A	0.96 (0.86-1.07)	0.502		0.95 (0.84-1.08)	0.47
HMDB0011220*	C36:5 PC plasmalogen-B	1.02 (0.91-1.13)	0.783		1.04 (0.91-1.18)	0.552
HMDB0011410*	C36:5 PE plasmalogen	1.06 (0.95-1.19)	0.294		1.07 (0.94-1.22)	0.32
HMDB0008270*	C38:2 PC	0.95 (0.85-1.07)	0.427		0.92 (0.8-1.06)	0.233
HMDB0008942*	C38:2 PE	0.98 (0.88-1.1)	0.755		1.06 (0.93-1.21)	0.366
HMDB0008047*	C38:3 PC	0.96 (0.85-1.09)	0.535		0.93 (0.81-1.08)	0.337
HMDB0011384*	C38:3 PE plasmalogen	0.99 (0.88-1.1)	0.796		1.09 (0.95-1.24)	0.213
HMDB0008048*	C38:4 PC	1.03 (0.92-1.16)	0.603		0.99 (0.86-1.13)	0.84
HMDB0011252*	C38:4 PC plasmalogen	1.06 (0.95-1.18)	0.317		1.04 (0.91-1.19)	0.525
HMDB0009003*	C38:4 PE	1.05 (0.93-1.19)	0.421		0.92 (0.8-1.06)	0.239

HMDB0007199*	C38:5 DAG	1.01 (0.9-1.13)	0.89		0.96 (0.83-1.09)	0.514
HMDB0009069*	C38:5 PE	1.06 (0.95-1.2)	0.303		0.9 (0.79-1.03)	0.14
HMDB0011386*	C38:5 PE plasmalogen	1.06 (0.95-1.19)	0.288		1.05 (0.92-1.2)	0.425
HMDB0007991*	C38:6 PC	1.02 (0.91-1.15)	0.723		1.05 (0.91-1.2)	0.526
HMDB0009102*	C38:6 PE	1.03 (0.91-1.16)	0.666		0.98 (0.85-1.13)	0.753
HMDB0011387*	C38:6 PE plasmalogen	1.03 (0.92-1.14)	0.636		1.03 (0.91-1.17)	0.646
HMDB0011229*	C38:7 PC plasmalogen	0.94 (0.84-1.05)	0.273		1.08 (0.95-1.23)	0.228
HMDB0011420*	C38:7 PE plasmalogen	1.04 (0.92-1.17)	0.533		1.1 (0.96-1.26)	0.158
HMDB0008511*	C40:10 PC	0.97 (0.87-1.09)	0.666		0.96 (0.84-1.09)	0.497
HMDB0008057*	C40:6 PC	0.99 (0.88-1.11)	0.884		1.03 (0.9-1.18)	0.631
HMDB0011394*	C40:7 PE plasmalogen	1.08 (0.96-1.2)	0.209		1.13 (0.99-1.29)	0.067
HMDB0008731*	C40:9 PC	1.01 (0.89-1.13)	0.915		1.04 (0.91-1.2)	0.529
HMDB0042062*	C43:0 TAG	0.96 (0.86-1.08)	0.474		0.93 (0.82-1.07)	0.321
HMDB0042098*	C43:1 TAG	0.92 (0.82-1.03)	0.15		0.9 (0.79-1.04)	0.144
HMDB0043169*	C43:2 TAG	0.93 (0.82-1.05)	0.232		0.92 (0.8-1.06)	0.247
HMDB0042063*	C44:0 TAG	0.94 (0.84-1.05)	0.285		0.89 (0.78-1.02)	0.1
HMDB0042099*	C45:1 TAG	0.93 (0.83-1.05)	0.242		0.91 (0.8-1.05)	0.19
HMDB0043170*	C45:2 TAG	0.94 (0.84-1.06)	0.316		0.92 (0.8-1.06)	0.258
HMDB0010411*	C46:0 TAG	0.93 (0.83-1.04)	0.212		0.87 (0.76-1)	0.051
HMDB0010412*	C46:1 TAG	0.93 (0.83-1.04)	0.213		0.89 (0.77-1.01)	0.08
HMDB0010419*	C46:2 TAG	0.94 (0.83-1.05)	0.261		0.89 (0.78-1.02)	0.107
HMDB0042100*	C47:1 TAG	0.95 (0.85-1.06)	0.354		0.95 (0.84-1.09)	0.465
HMDB0042076*	C47:2 TAG	0.94 (0.83-1.05)	0.256		0.93 (0.81-1.06)	0.282
HMDB0005356*	C48:0 TAG	0.92 (0.82-1.04)	0.19		0.89 (0.78-1.02)	0.105
HMDB0005359*	C48:1 TAG	0.93 (0.83-1.05)	0.238		0.88 (0.76-1.01)	0.061
HMDB0005376*	C48:2 TAG	0.93 (0.83-1.05)	0.261		0.89 (0.78-1.02)	0.099
HMDB0005432*	C48:3 TAG	0.93 (0.83-1.05)	0.235		0.89 (0.77-1.02)	0.091
HMDB0011706*	C49:2 TAG	0.94 (0.84-1.05)	0.304		0.95 (0.83-1.08)	0.438
HMDB0042103*	C49:3 TAG	0.96 (0.86-1.08)	0.489		0.96 (0.84-1.09)	0.502
HMDB0005357*	C50:0 TAG	0.92 (0.81-1.03)	0.148		0.87 (0.76-1)	0.053
HMDB0005360*	C50:1 TAG	0.93 (0.82-1.05)	0.242		0.86 (0.75-0.99)	0.042
HMDB0005377*	C50:2 TAG	0.94 (0.83-1.06)	0.306		0.86 (0.74-0.99)	0.034
HMDB0005433*	C50:3 TAG	0.98 (0.87-1.1)	0.744		0.9 (0.79-1.04)	0.143
HMDB0005435*	C50:4 TAG	0.97 (0.86-1.09)	0.564		0.9 (0.78-1.03)	0.128
HMDB0010471*	C50:5 TAG	0.94 (0.84-1.05)	0.285		0.87 (0.76-1)	0.052
HMDB0010497*	C50:6 TAG	0.93 (0.83-1.04)	0.203		0.9 (0.78-1.04)	0.143
HMDB0031106*	C51:0 TAG	0.93 (0.83-1.04)	0.202		0.91 (0.8-1.05)	0.191
HMDB0042104*	C51:1 TAG	0.91 (0.81-1.02)	0.112		0.94 (0.82-1.07)	0.354
HMDB0005362*	C51:2 TAG	0.92 (0.82-1.03)	0.154		0.93 (0.81-1.06)	0.257

HMDB0011701*	C51:3 TAG	0.98 (0.88-1.1)	0.76		0.98 (0.86-1.12)	0.821
HMDB0005365*	C52:0 TAG	0.94 (0.83-1.06)	0.289		0.85 (0.74-0.98)	0.025
HMDB0005367*	C52:1 TAG	0.9 (0.8-1.02)	0.108		0.83 (0.72-0.96)	0.013
HMDB0005369*	C52:2 TAG	0.97 (0.86-1.1)	0.662		0.87 (0.75-1)	0.045
HMDB0005384*	C52:3 TAG	1 (0.89-1.12)	0.988		0.91 (0.8-1.05)	0.194
HMDB0005363*	C52:4 TAG	1 (0.9-1.12)	0.97		0.98 (0.86-1.11)	0.731
HMDB0005380*	C52:5 TAG	1.02 (0.91-1.14)	0.766		0.95 (0.83-1.08)	0.446
HMDB0005436*	C52:6 TAG	0.97 (0.87-1.09)	0.649		0.9 (0.78-1.03)	0.115
HMDB0010517*	C52:7 TAG	0.95 (0.85-1.06)	0.351		0.9 (0.78-1.03)	0.134
HMDB0042196*	C53:2 TAG	0.95 (0.84-1.06)	0.349		0.94 (0.82-1.07)	0.361
HMDB0043058*	C53:3 TAG	0.99 (0.89-1.11)	0.869		1.01 (0.89-1.15)	0.911
HMDB0005395*	C54:1 TAG	0.95 (0.85-1.07)	0.437		0.87 (0.75-1)	0.047
HMDB0005403*	C54:2 TAG	0.98 (0.87-1.1)	0.727		0.86 (0.75-0.99)	0.032
HMDB0005405*	C54:3 TAG	1.05 (0.94-1.17)	0.394		0.92 (0.81-1.05)	0.234
HMDB0005370*	C54:4 TAG	1.05 (0.94-1.18)	0.357		0.97 (0.85-1.1)	0.651
HMDB0005385*	C54:5 TAG	0.94 (0.84-1.06)	0.335		0.9 (0.78-1.04)	0.149
HMDB0005391*	C54:6 TAG	1 (0.9-1.12)	0.943		0.97 (0.85-1.1)	0.656
HMDB0005447*	C54:7 TAG	0.97 (0.87-1.08)	0.529		0.95 (0.84-1.08)	0.458
HMDB0010518*	C54:8 TAG	0.95 (0.85-1.06)	0.336		0.94 (0.82-1.07)	0.351
HMDB0010498*	C54:9 TAG	0.95 (0.84-1.06)	0.334		0.97 (0.85-1.11)	0.684
HMDB0042226*	C55:2 TAG	0.97 (0.86-1.09)	0.579		0.9 (0.78-1.03)	0.122
HMDB0042466*	C55:3 TAG	1.03 (0.92-1.15)	0.613		1.01 (0.89-1.14)	0.889
HMDB0005396*	C56:1 TAG	0.96 (0.86-1.08)	0.539		0.91 (0.79-1.05)	0.196
HMDB0010513*	C56:10 TAG	0.96 (0.85-1.07)	0.435		0.99 (0.86-1.13)	0.85
HMDB0005404*	C56:2 TAG	0.98 (0.87-1.1)	0.754		0.92 (0.8-1.05)	0.216
HMDB0005410*	C56:3 TAG	1.04 (0.93-1.16)	0.511		0.91 (0.8-1.04)	0.163
HMDB0005398*	C56:4 TAG	1.07 (0.96-1.2)	0.211		1 (0.88-1.13)	0.973
HMDB0005406*	C56:5 TAG	1.09 (0.97-1.22)	0.134		1.09 (0.95-1.24)	0.216
HMDB0005456*	C56:6 TAG	1.04 (0.93-1.16)	0.524		1.04 (0.91-1.18)	0.571
HMDB0005462*	C56:7 TAG	1.04 (0.93-1.16)	0.522		1.05 (0.92-1.2)	0.444
HMDB0005392*	C56:8 TAG	1.02 (0.91-1.14)	0.744		1.13 (0.99-1.28)	0.07
HMDB0005448*	C56:9 TAG	0.97 (0.87-1.09)	0.606		1.03 (0.91-1.18)	0.614
HMDB0010531*	C58:11 TAG	0.95 (0.85-1.07)	0.408		1.04 (0.91-1.19)	0.566
HMDB0005458*	C58:6 TAG	1.05 (0.94-1.18)	0.378		1.07 (0.94-1.22)	0.314
HMDB0005471*	C58:7 TAG	1.11 (0.99-1.24)	0.082		1.09 (0.96-1.24)	0.162
HMDB0005463*	C58:9 TAG	1.03 (0.92-1.16)	0.574		1.14 (1-1.31)	0.051
HMDB0005478*	C60:12 TAG	1.01 (0.9-1.13)	0.917		1.16 (1.01-1.33)	0.033
HMDB0002869	campesterol	0.98 (0.87-1.1)	0.701		0.89 (0.77-1.01)	0.078
HMDB0000626	chenodeoxycholate/deoxycholate	0.96 (0.86-1.07)	0.467		0.97 (0.85-1.1)	0.608

HMDB0000067	cholesterol	0.98 (0.87-1.1)	0.734		0.89 (0.78-1.02)	0.098
HMDB0061112	CMPF	1.01 (0.9-1.13)	0.891		1.07 (0.93-1.22)	0.339
HMDB0001072	coenzyme Q10	0.95 (0.84-1.06)	0.328		1 (0.88-1.14)	0.954
HMDB0000613*	erythronate/threonate	0.97 (0.86-1.08)	0.542		1.08 (0.94-1.23)	0.262
HMDB0000174	fucose	1.04 (0.93-1.16)	0.502		0.96 (0.84-1.1)	0.59
HMDB0000152	gentisate	0.97 (0.85-1.1)	0.614		1.04 (0.89-1.2)	0.644
HMDB0000148	glutamate	1.02 (0.9-1.14)	0.794		1 (0.87-1.15)	0.97
HMDB0000139	glycerate	1.01 (0.9-1.13)	0.881		1.06 (0.93-1.21)	0.377
HMDB0001401*	hexose monophosphate	0.95 (0.85-1.06)	0.388		1.15 (1-1.32)	0.043
HMDB0000118	homovanillate	1.1 (0.98-1.23)	0.106		0.93 (0.82-1.07)	0.325
HMDB0002302	indole-3-propionate	1.06 (0.95-1.19)	0.295		1.06 (0.93-1.22)	0.393
HMDB0000197	indoleacetate	0.91 (0.81-1.02)	0.102		0.97 (0.85-1.1)	0.624
HMDB0000682	indoxylsulfate	0.86 (0.77-0.96)	0.006		0.96 (0.84-1.09)	0.494
HMDB0000684	kynurenine	0.96 (0.86-1.08)	0.537		0.86 (0.76-0.99)	0.031
HMDB0000691	malonate	0.99 (0.89-1.11)	0.902		0.9 (0.79-1.02)	0.112
HMDB0006112	MDA	1.02 (0.91-1.14)	0.778		1.09 (0.95-1.24)	0.203
HMDB0000749	mesaconate	1.01 (0.91-1.13)	0.819		1.11 (0.97-1.27)	0.121
HMDB0001138	N-acetylglutamate	1.04 (0.92-1.17)	0.526		1.07 (0.92-1.23)	0.369
HMDB0002100	palmitoylethanolamide	1.08 (0.97-1.21)	0.167		1.02 (0.9-1.17)	0.742
HMDB0001548	pentose monophosphate	1 (0.89-1.13)	0.992		1.06 (0.93-1.21)	0.391
HMDB0000779	phenyllactate	1.09 (0.97-1.22)	0.159		0.95 (0.83-1.08)	0.441
HMDB0000232	quinolinate	0.94 (0.84-1.05)	0.284		0.93 (0.81-1.06)	0.273
HMDB0000893	suberate	0.99 (0.89-1.1)	0.841		0.97 (0.86-1.11)	0.669
HMDB0000896*	taurodeoxycholate/taurochenodeoxycholate	1.12 (0.99-1.27)	0.065		1.1 (0.96-1.27)	0.175
HMDB0004136	threitol	1.03 (0.92-1.16)	0.558		1.04 (0.91-1.2)	0.533
HMDB0000262	thymine	1.04 (0.93-1.17)	0.458		1 (0.88-1.14)	0.986
HMDB0000300	uracil	0.99 (0.88-1.11)	0.834		1.08 (0.94-1.24)	0.275
HMDB0000881	xanthurenate	0.91 (0.81-1.01)	0.089		0.99 (0.87-1.13)	0.911

Model adjusted for BMI at age 18, weight change (from age 18 to time of first blood draw), age at menarche, parity and age at first birth, breastfeeding history, family history of breast cancer in a first degree relative, personal history of benign breast disease, physical activity, alcohol intake (by quintile), and oral contraceptive use at blood collection
