# Supplementary material for: Prospective study of circulating metabolomic profiles and breast cancer incidence among predominantly premenopausal women
Source: Br J Cancer. Author manuscript; Available in PMC 2025 Dec 6. (PMC12572396; doi:10.1038/s41416-025-03159-2)
Supplement: Suppl Table 6 [file NIHMS2109610-supplement-Suppl_Table_6.pdf]

**Supplemental Table 6**. Modules were identified from the WGCNA analysis (based on the complete set of 381 metabolites across three profiling platforms)

| HMDB_ID | METABOLITE | class_broad | Module |
|---|---|---|---|
| **HMDB0015150** | sulfamethoxazole | Benzene and substituted derivatives | M1 grey |
| **HMDB0000301** | urocanic acid | NA | M1 grey |
| **HMDB0000538** | ATP | NA | M1 grey |
| **HMDB0000982** | 5-methylcytidine | NA | M1 grey |
| **HMDB0001850** | verapamil | NA | M1 grey |
| **HMDB0001921** | metformin | NA | M1 grey |
| **HMDB0001930** | ranitidine | NA | M1 grey |
| **HMDB0001932** | metoprolol | NA | M1 grey |
| **HMDB0005015** | gabapentin | NA | M1 grey |
| **HMDB0010316** | acetaminophen glucuronide | NA | M1 grey |
| **HMDB0011756** | N-acetylleucine | NA | M1 grey |
| **HMDB0014323** | valsartan | NA | M1 grey |
| **HMDB0015028** | sulfapyridine | NA | M1 grey |
| **HMDB0015052** | metronidazole | NA | M1 grey |
| **HMDB0001046** | cotinine | Organoheterocyclic compounds | M1 grey |
| **HMDB0001390** | hydroxycotinine | Organoheterocyclic compounds | M1 grey |
| **HMDB0001859** | acetaminophen | Phenols | M1 grey |
| **HMDB0000610*** | C18:2 CE | Cholesteryl esters | M2 cadetblue1 |
| **HMDB0000885** | C16:0 CE | Cholesteryl esters | M2 cadetblue1 |
| **HMDB0000918*** | C18:1 CE | Cholesteryl esters | M2 cadetblue1 |
| **HMDB0006726** | C20:4 CE | Cholesteryl esters | M2 cadetblue1 |
| **HMDB0006731** | C20:5 CE | Cholesteryl esters | M2 cadetblue1 |
| **HMDB0006733** | C22:6 CE | Cholesteryl esters | M2 cadetblue1 |
| **HMDB0006736*** | C20:3 CE | Cholesteryl esters | M2 cadetblue1 |
| **HMDB0010370*** | C18:3 CE | Cholesteryl esters | M2 cadetblue1 |
| **HMDB0010375*** | C22:5 CE | Cholesteryl esters | M2 cadetblue1 |
| **HMDB0007011*** | C30:0 DAG | Diglycerides | M2 cadetblue1 |
| **HMDB0007098*** | C32:0 DAG | Diglycerides | M2 cadetblue1 |
| **HMDB0007099*** | C32:1 DAG | Diglycerides | M2 cadetblue1 |
| **HMDB0007100*** | C34:0 DAG | Diglycerides | M2 cadetblue1 |
| **HMDB0007102*** | C34:1 DAG | Diglycerides | M2 cadetblue1 |
| **HMDB0007103*** | C34:2 DAG | Diglycerides | M2 cadetblue1 |
| **HMDB0007132*** | C34:3 DAG | Diglycerides | M2 cadetblue1 |
| **HMDB0007199*** | C38:5 DAG | Diglycerides | M2 cadetblue1 |
| **HMDB0007216*** | C36:1 DAG | Diglycerides | M2 cadetblue1 |

| | | | |
|---|---|---|---|
| **HMDB0007218*** | C36:2 DAG | Diglycerides | M2 cadetblue1 |
| **HMDB0007219*** | C36:3 DAG | Diglycerides | M2 cadetblue1 |
| **HMDB0010379** | C14:0 LPC | Lysophosphatidylcholines | M2 cadetblue1 |
| **HMDB0007869*** | C30:0 PC | Phosphatidylcholines | M2 cadetblue1 |
| **HMDB0007870*** | C30:1 PC | Phosphatidylcholines | M2 cadetblue1 |
| **HMDB0007873*** | C32:1 PC | Phosphatidylcholines | M2 cadetblue1 |
| **HMDB0007874*** | C32:2 PC | Phosphatidylcholines | M2 cadetblue1 |
| **HMDB0007883*** | C34:4 PC | Phosphatidylcholines | M2 cadetblue1 |
| **HMDB0007972*** | C34:1 PC | Phosphatidylcholines | M2 cadetblue1 |
| **HMDB0008038*** | C36:1 PC | Phosphatidylcholines | M2 cadetblue1 |
| **HMDB0008047*** | C38:3 PC | Phosphatidylcholines | M2 cadetblue1 |
| **HMDB0008923*** | C32:0 PE | Phosphatidylethanolamines | M2 cadetblue1 |
| **HMDB0008925*** | C34:0 PE | Phosphatidylethanolamines | M2 cadetblue1 |
| **HMDB0008928*** | C34:2 PE | Phosphatidylethanolamines | M2 cadetblue1 |
| **HMDB0008937*** | C36:4 PE | Phosphatidylethanolamines | M2 cadetblue1 |
| **HMDB0008993*** | C36:1 PE | Phosphatidylethanolamines | M2 cadetblue1 |
| **HMDB0008994*** | C36:2 PE | Phosphatidylethanolamines | M2 cadetblue1 |
| **HMDB0009003*** | C38:4 PE | Phosphatidylethanolamines | M2 cadetblue1 |
| **HMDB0009012*** | C40:6 PE | Phosphatidylethanolamines | M2 cadetblue1 |
| **HMDB0009060*** | C36:3 PE | Phosphatidylethanolamines | M2 cadetblue1 |
| **HMDB0009069*** | C38:5 PE | Phosphatidylethanolamines | M2 cadetblue1 |
| **HMDB0009102*** | C38:6 PE | Phosphatidylethanolamines | M2 cadetblue1 |
| **HMDB0005356*** | C48:0 TAG | Triglycerides | M2 cadetblue1 |
| **HMDB0005357*** | C50:0 TAG | Triglycerides | M2 cadetblue1 |
| **HMDB0005359*** | C48:1 TAG | Triglycerides | M2 cadetblue1 |
| **HMDB0005360*** | C50:1 TAG | Triglycerides | M2 cadetblue1 |
| **HMDB0005362*** | C51:2 TAG | Triglycerides | M2 cadetblue1 |
| **HMDB0005363*** | C52:4 TAG | Triglycerides | M2 cadetblue1 |
| **HMDB0005365*** | C52:0 TAG | Triglycerides | M2 cadetblue1 |
| **HMDB0005367*** | C52:1 TAG | Triglycerides | M2 cadetblue1 |
| **HMDB0005369*** | C52:2 TAG | Triglycerides | M2 cadetblue1 |
| **HMDB0005376*** | C48:2 TAG | Triglycerides | M2 cadetblue1 |
| **HMDB0005377*** | C50:2 TAG | Triglycerides | M2 cadetblue1 |
| **HMDB0005384*** | C52:3 TAG | Triglycerides | M2 cadetblue1 |
| **HMDB0005385*** | C54:5 TAG | Triglycerides | M2 cadetblue1 |
| **HMDB0005395*** | C54:1 TAG | Triglycerides | M2 cadetblue1 |
| **HMDB0005396*** | C56:1 TAG | Triglycerides | M2 cadetblue1 |
| **HMDB0005403*** | C54:2 TAG | Triglycerides | M2 cadetblue1 |
| **HMDB0005404*** | C56:2 TAG | Triglycerides | M2 cadetblue1 |

| | | | |
|---|---|---|---|
| **HMDB0005405*** | C54:3 TAG | Triglycerides | M2 cadetblue1 |
| **HMDB0005410*** | C56:3 TAG | Triglycerides | M2 cadetblue1 |
| **HMDB0005432*** | C48:3 TAG | Triglycerides | M2 cadetblue1 |
| **HMDB0005433*** | C50:3 TAG | Triglycerides | M2 cadetblue1 |
| **HMDB0005435*** | C50:4 TAG | Triglycerides | M2 cadetblue1 |
| **HMDB0010411*** | C46:0 TAG | Triglycerides | M2 cadetblue1 |
| **HMDB0010412*** | C46:1 TAG | Triglycerides | M2 cadetblue1 |
| **HMDB0010419*** | C46:2 TAG | Triglycerides | M2 cadetblue1 |
| **HMDB0011701*** | C51:3 TAG | Triglycerides | M2 cadetblue1 |
| **HMDB0011705*** | C49:1 TAG | Triglycerides | M2 cadetblue1 |
| **HMDB0011706*** | C49:2 TAG | Triglycerides | M2 cadetblue1 |
| **HMDB0031106*** | C51:0 TAG | Triglycerides | M2 cadetblue1 |
| **HMDB0042062*** | C43:0 TAG | Triglycerides | M2 cadetblue1 |
| **HMDB0042063*** | C44:0 TAG | Triglycerides | M2 cadetblue1 |
| **HMDB0042076*** | C47:2 TAG | Triglycerides | M2 cadetblue1 |
| **HMDB0042093*** | C45:0 TAG | Triglycerides | M2 cadetblue1 |
| **HMDB0042098*** | C43:1 TAG | Triglycerides | M2 cadetblue1 |
| **HMDB0042099*** | C45:1 TAG | Triglycerides | M2 cadetblue1 |
| **HMDB0042100*** | C47:1 TAG | Triglycerides | M2 cadetblue1 |
| **HMDB0042103*** | C49:3 TAG | Triglycerides | M2 cadetblue1 |
| **HMDB0042104*** | C51:1 TAG | Triglycerides | M2 cadetblue1 |
| **HMDB0042196*** | C53:2 TAG | Triglycerides | M2 cadetblue1 |
| **HMDB0042226*** | C55:2 TAG | Triglycerides | M2 cadetblue1 |
| **HMDB0043058*** | C53:3 TAG | Triglycerides | M2 cadetblue1 |
| **HMDB0043169*** | C43:2 TAG | Triglycerides | M2 cadetblue1 |
| **HMDB0043170*** | C45:2 TAG | Triglycerides | M2 cadetblue1 |
| **HMDB0029377** | piperine | Alkaloids and derivatives | M3 cornflowerblue |
| **HMDB0001476** | 3-hydroxyanthranilic acid | Benzenoids | M3 cornflowerblue |
| **HMDB0000092** | dimethylglycine | Carboxylic acids and derivatives | M3 cornflowerblue |
| **HMDB0000158** | tyrosine | Carboxylic acids and derivatives | M3 cornflowerblue |
| **HMDB0000177** | histidine | Carboxylic acids and derivatives | M3 cornflowerblue |
| **HMDB0000182** | lysine | Carboxylic acids and derivatives | M3 cornflowerblue |
| **HMDB0000248** | thyroxine | Carboxylic acids and derivatives | M3 cornflowerblue |
| **HMDB0000687** | leucine | Carboxylic acids and derivatives | M3 cornflowerblue |
| **HMDB0000725** | hydroxyproline | Carboxylic acids and derivatives | M3 cornflowerblue |
| **HMDB0000883** | valine | Carboxylic acids and derivatives | M3 cornflowerblue |

| | | | |
|---|---|---|---|
| **HMDB0000062** | carnitine | Carnitines | M3 cornflowerblue |
| **HMDB0000688** | C5 carnitine | Carnitines | M3 cornflowerblue |
| **HMDB0000824** | C3 carnitine | Carnitines | M3 cornflowerblue |
| **HMDB0002013** | C4 carnitine | Carnitines | M3 cornflowerblue |
| **HMDB0002366** | C5:1 carnitine | Carnitines | M3 cornflowerblue |
| **HMDB0000019** | alpha-ketoisovalerate | NA | M3 cornflowerblue |
| **HMDB0000232** | quinolinate | NA | M3 cornflowerblue |
| **HMDB0000262** | thymine | NA | M3 cornflowerblue |
| **HMDB0000317*** | 2-hydroxy-3-methylpentanoate/hydroxyisocaproate | NA | M3 cornflowerblue |
| **HMDB0000407** | 2-hydroxy-3-methylbutyrate | NA | M3 cornflowerblue |
| **HMDB0000491*** | alpha-keto-beta-methylvalerate/alpha-ketoisocaproate | NA | M3 cornflowerblue |
| **HMDB0000650** | 2-aminobutyrate | NA | M3 cornflowerblue |
| **HMDB0000715** | kynurenic acid | NA | M3 cornflowerblue |
| **HMDB0000719** | homoserine | NA | M3 cornflowerblue |
| **HMDB0000881** | xanthurenate | NA | M3 cornflowerblue |
| **HMDB0001072** | coenzyme Q10 | NA | M3 cornflowerblue |
| **HMDB0002302** | indole-3-propionate | NA | M3 cornflowerblue |
| **HMDB0004207** | alpha-glutamyllysine | NA | M3 cornflowerblue |
| **HMDB0032055** | N-acetylhistidine | NA | M3 cornflowerblue |
| **HMDB0059824** | 4-hydroxy-3-methylacetophenone | NA | M3 cornflowerblue |
| **HMDB0094649** | 2-aminoheptanoate | NA | M3 cornflowerblue |
| **HMDB0000884** | ribothymidine | Nucleosides, nucleotides, and analogues | M3 cornflowerblue |
| **HMDB0000064** | creatine | Organic acids and derivatives | M3 cornflowerblue |
| **HMDB0000112** | GABA | Organic acids and derivatives | M3 cornflowerblue |
| **HMDB0000159** | phenylalanine | Organic acids and derivatives | M3 cornflowerblue |
| **HMDB0000167** | threonine | Organic acids and derivatives | M3 cornflowerblue |
| **HMDB0000168** | asparagine | Organic acids and derivatives | M3 cornflowerblue |
| **HMDB0000172** | isoleucine | Organic acids and derivatives | M3 cornflowerblue |
| **HMDB0000206** | N6-acetyllysine | Organic acids and derivatives | M3 cornflowerblue |
| **HMDB0000641** | glutamine | Organic acids and derivatives | M3 cornflowerblue |

| | | | |
|---|---|---|---|
| **HMDB0000696** | methionine | Organic acids and derivatives | M3 cornflowerblue |
| **HMDB0000904** | citrulline | Organic acids and derivatives | M3 cornflowerblue |
| **HMDB0001539** | ADMA | Organic acids and derivatives | M3 cornflowerblue |
| **HMDB0003334** | SDMA | Organic acids and derivatives | M3 cornflowerblue |
| **HMDB0004620** | N-alpha-acetyarginine | Organic acids and derivatives | M3 cornflowerblue |
| **HMDB0013287** | N6,N6-dimethyllysine | Organic acids and derivatives | M3 cornflowerblue |
| **HMDB0013713** | N-acetyltryptophan | Organic acids and derivatives | M3 cornflowerblue |
| **HMDB0029416** | NMMA | Organic acids and derivatives | M3 cornflowerblue |
| **HMDB0000929** | tryptophan | Organoheterocyclic compounds | M3 cornflowerblue |
| **HMDB0002271** | imidazole propionate | Organoheterocyclic compounds | M3 cornflowerblue |
| **HMDB0011220*** | C36:5 PC plasmalogen-B | PC plasmalogens | M3 cornflowerblue |
| **HMDB0011239*** | C34:1 PC plasmalogen-B | PC plasmalogens | M3 cornflowerblue |
| **HMDB0000779** | phenyllactate | Phenylpropanoids and polyketides | M3 cornflowerblue |
| **HMDB0011208*** | C34:1 PC plasmalogen | Phosphatidylcholine plasmalogens | M3 cornflowerblue |
| **HMDB0011210*** | C34:2 PC plasmalogen | Phosphatidylcholine plasmalogens | M3 cornflowerblue |
| **HMDB0011211*** | C34:3 PC plasmalogen | Phosphatidylcholine plasmalogens | M3 cornflowerblue |
| **HMDB0011214*** | C34:5 PC plasmalogen | Phosphatidylcholine plasmalogens | M3 cornflowerblue |
| **HMDB0011243*** | C36:2 PC plasmalogen | Phosphatidylcholine plasmalogens | M3 cornflowerblue |
| **HMDB0011244*** | C36:3 PC plasmalogen | Phosphatidylcholine plasmalogens | M3 cornflowerblue |
| **HMDB0011252*** | C38:4 PC plasmalogen | Phosphatidylcholine plasmalogens | M3 cornflowerblue |
| **HMDB0011310*** | C36:4 PC plasmalogen | Phosphatidylcholine plasmalogens | M3 cornflowerblue |
| **HMDB0008952*** | C34:2 PE plasmalogen | Phosphatidylethanolamine plasmalogens | M3 cornflowerblue |
| **HMDB0009016*** | C36:1 PE plasmalogen | Phosphatidylethanolamine plasmalogens | M3 cornflowerblue |
| **HMDB0009082*** | C36:2 PE plasmalogen | Phosphatidylethanolamine plasmalogens | M3 cornflowerblue |
| **HMDB0011253*** | C38:5 PE plasmalogen | Phosphatidylethanolamine plasmalogens | M3 cornflowerblue |
| **HMDB0011343*** | C34:3 PE plasmalogen | Phosphatidylethanolamine plasmalogens | M3 cornflowerblue |
| **HMDB0011384*** | C38:3 PE plasmalogen | Phosphatidylethanolamine plasmalogens | M3 cornflowerblue |
| **HMDB0011386*** | C38:5 PE plasmalogen | Phosphatidylethanolamine plasmalogens | M3 cornflowerblue |
| **HMDB0011387*** | C38:6 PE plasmalogen | Phosphatidylethanolamine plasmalogens | M3 cornflowerblue |
| **HMDB0011410*** | C36:5 PE plasmalogen | Phosphatidylethanolamine plasmalogens | M3 cornflowerblue |

| | | | |
|---|---|---|---|
| **HMDB0011441*** | C36:3 PE plasmalogen | Phosphatidylethanolamine plasmalogens | M3 cornflowerblue |
| **HMDB0011442*** | C36:4 PE plasmalogen | Phosphatidylethanolamine plasmalogens | M3 cornflowerblue |
| **HMDB0001431** | pyridoxamine | Pyridines and derivatives | M3 cornflowerblue |
| **HMDB0000210** | pantothenate | Carboxylic acids and derivatives | M4 darkgreen |
| **HMDB0000201** | C2 carnitine | Carnitines | M4 darkgreen |
| **HMDB0000651** | C10 carnitine | Carnitines | M4 darkgreen |
| **HMDB0000705** | C6 carnitine | Carnitines | M4 darkgreen |
| **HMDB0000791** | C8 carnitine | Carnitines | M4 darkgreen |
| **HMDB0002014** | C14:1 carnitine | Carnitines | M4 darkgreen |
| **HMDB0002250** | C12 carnitine | Carnitines | M4 darkgreen |
| **HMDB0005066** | C14 carnitine | Carnitines | M4 darkgreen |
| **HMDB0013127** | C4-OH carnitine | Carnitines | M4 darkgreen |
| **HMDB0013130** | C5-DC carnitine | Carnitines | M4 darkgreen |
| **HMDB0013238** | C7 carnitine | Carnitines | M4 darkgreen |
| **HMDB0013288** | C9 carnitine | Carnitines | M4 darkgreen |
| **HMDB0013325** | C10:2 carnitine | Carnitines | M4 darkgreen |
| **HMDB0013326** | C12:1 carnitine | Carnitines | M4 darkgreen |
| **HMDB0013331** | C14:2 carnitine | Carnitines | M4 darkgreen |
| **HMDB0000630** | cytosine | Diazines | M4 darkgreen |
| **HMDB0000008*** | alpha-hydroxybutyrate/beta-hydroxybutyrate/hydroxyisobutyrate | NA | M4 darkgreen |
| **HMDB0000125** | glutathione reduced | NA | M4 darkgreen |
| **HMDB0000139** | glycerate | NA | M4 darkgreen |
| **HMDB0000174** | fucose | NA | M4 darkgreen |
| **HMDB0000191** | aspartate | NA | M4 darkgreen |
| **HMDB0000235** | thiamine | NA | M4 darkgreen |
| **HMDB0000259** | serotonin | NA | M4 darkgreen |
| **HMDB0000300** | uracil | NA | M4 darkgreen |
| **HMDB0000355** | 3-hydroxymethylglutarate/anhydroDglucose | NA | M4 darkgreen |
| **HMDB0000448*** | adipate/methylglutarate | NA | M4 darkgreen |
| **HMDB0000544** | 5-hydroxymethyl-4-methyluracil | NA | M4 darkgreen |
| **HMDB0000613*** | erythronate/threonate | NA | M4 darkgreen |
| **HMDB0000684** | kynurenine | NA | M4 darkgreen |
| **HMDB0000691** | malonate | NA | M4 darkgreen |
| **HMDB0000749** | mesaconate | NA | M4 darkgreen |
| **HMDB0000807** | 3-phosphoglycerate | NA | M4 darkgreen |
| **HMDB0000840** | salicylurate | NA | M4 darkgreen |

| | | | |
|---|---|---|---|
| **HMDB0000853** | N-acetylgalactosamine | NA | M4 darkgreen |
| **HMDB0000893** | suberate | NA | M4 darkgreen |
| **HMDB0000898** | 1-methylhistamine | NA | M4 darkgreen |
| **HMDB0001138** | N-acetylglutamate | NA | M4 darkgreen |
| **HMDB0001257** | spermidine | NA | M4 darkgreen |
| **HMDB0001401*** | hexose monophosphate | NA | M4 darkgreen |
| **HMDB0001563** | 1-methylguanosine | NA | M4 darkgreen |
| **HMDB0001565** | phosphocholine | NA | M4 darkgreen |
| **HMDB0001924** | atenolol | NA | M4 darkgreen |
| **HMDB0002088** | N-oleoylethanolamine | NA | M4 darkgreen |
| **HMDB0002100** | palmitoylethanolamide | NA | M4 darkgreen |
| **HMDB0003282** | 1-methylguanine | NA | M4 darkgreen |
| **HMDB0003464** | 4-guanidinobutanoic acid | NA | M4 darkgreen |
| **HMDB0004030** | 21-deoxycortisol | NA | M4 darkgreen |
| **HMDB0004158** | urobilinogen | NA | M4 darkgreen |
| **HMDB0005862** | 2-methylguanosine | NA | M4 darkgreen |
| **HMDB0006112** | MDA | NA | M4 darkgreen |
| **HMDB0011745** | N-acetylmethionine | NA | M4 darkgreen |
| **HMDB0013631** | oleoyl glycine | NA | M4 darkgreen |
| **HMDB0015168** | cerulenin | NA | M4 darkgreen |
| **HMDB0034169** | methyl N-methylanthranilate | NA | M4 darkgreen |
| **HMDB0000767** | pseudouridine | Nucleosides, nucleotides, and analogues | M4 darkgreen |
| **HMDB0004824** | N2,N2-dimethylguanosine | Nucleosides, nucleotides, and analogues | M4 darkgreen |
| **HMDB0005923** | N4-acetylcytidine | Nucleosides, nucleotides, and analogues | M4 darkgreen |
| **HMDB0000679** | homocitrulline | Organic acids and derivatives | M4 darkgreen |
| **HMDB0001906*** | aminoisobutyric acid | Organic acids and derivatives | M4 darkgreen |
| **HMDB0002172** | N1,N12-diacetylspermine | Organic acids and derivatives | M4 darkgreen |
| **HMDB0003357** | N-acetylornithine | Organic acids and derivatives | M4 darkgreen |
| **HMDB0003681** | 4-acetamidobutanoate | Organic acids and derivatives | M4 darkgreen |
| **HMDB0000017** | 4-pyridoxate | Organoheterocyclic compounds | M4 darkgreen |
| **HMDB0000897** | 7-methylguanine | Organoheterocyclic compounds | M4 darkgreen |
| **HMDB0004193** | N1-methyl-2-pyridone-5-carboxamide | Organoheterocyclic compounds | M4 darkgreen |
| **HMDB0001414** | putrescine | Organonitrogen compounds | M4 darkgreen |
| **HMDB0000699** | 1-methylnicotinamide | Pyridines and derivatives | M4 darkgreen |
| **HMDB0000063** | cortisol | Steroids and steroid derivatives | M4 darkgreen |

| | | | |
|---|---|---|---|
| **HMDB0002802** | cortisone | Steroids and steroid derivatives | M4 darkgreen |
| **HMDB0000043** | betaine | Carboxylic acids and derivatives | M5 darkolivegreen |
| **HMDB0000123** | glycine | Carboxylic acids and derivatives | M5 darkolivegreen |
| **HMDB0000128** | guanidinoacetic acid | Carboxylic acids and derivatives | M5 darkolivegreen |
| **HMDB0000161** | alanine | Carboxylic acids and derivatives | M5 darkolivegreen |
| **HMDB0000162** | proline | Carboxylic acids and derivatives | M5 darkolivegreen |
| **HMDB0000187** | serine | Carboxylic acids and derivatives | M5 darkolivegreen |
| **HMDB0000658*** | C16:1 CE | Cholesteryl esters | M5 darkolivegreen |
| **HMDB0006725** | C14:0 CE | Cholesteryl esters | M5 darkolivegreen |
| **HMDB0010368** | C18:0 CE | Cholesteryl esters | M5 darkolivegreen |
| **HMDB0007248*** | C36:4 DAG | Diglycerides | M5 darkolivegreen |
| **HMDB0000289** | urate | Imidazopyrimidines | M5 darkolivegreen |
| **HMDB0011478*** | C18:3 LPE | Lysophosphatidylethanolami nes | M5 darkolivegreen |
| **HMDB0000054** | bilirubin | NA | M5 darkolivegreen |
| **HMDB0000118** | homovanillate | NA | M5 darkolivegreen |
| **HMDB0000508*** | adonitol/arabitol | NA | M5 darkolivegreen |
| **HMDB0000510** | 2-aminoadipate | NA | M5 darkolivegreen |
| **HMDB0000694** | 2-hydroxyglutarate | NA | M5 darkolivegreen |
| **HMDB0004136** | threitol | NA | M5 darkolivegreen |
| **HMDB0000716** | pipecolic acid | Organic acids and derivatives | M5 darkolivegreen |
| **HMDB0000991*** | 2-aminooctanoic acid | Organic acids and derivatives | M5 darkolivegreen |
| **HMDB0001276** | N1-acetylspermidine | Organic acids and derivatives | M5 darkolivegreen |
| **HMDB0002064** | N-acetylputrescine | Organic acids and derivatives | M5 darkolivegreen |
| **HMDB0001008** | biliverdin | Organoheterocyclic compounds | M5 darkolivegreen |
| **HMDB0007871*** | C32:0 PC | Phosphatidylcholines | M5 darkolivegreen |
| **HMDB0008991*** | C36:0 PE | Phosphatidylethanolamines | M5 darkolivegreen |
| **HMDB0000626** | chenodeoxycholate/deoxycholate | Steroids and steroid derivatives | M5 darkolivegreen |
| **HMDB0005370*** | C54:4 TAG | Triglycerides | M5 darkolivegreen |
| **HMDB0005380*** | C52:5 TAG | Triglycerides | M5 darkolivegreen |
| **HMDB0005391*** | C54:6 TAG | Triglycerides | M5 darkolivegreen |

| | | | |
|---|---|---|---|
| **HMDB0005398*** | C56:4 TAG | Triglycerides | M5 darkolivegreen |
| **HMDB0005436*** | C52:6 TAG | Triglycerides | M5 darkolivegreen |
| **HMDB0005447*** | C54:7 TAG | Triglycerides | M5 darkolivegreen |
| **HMDB0010471*** | C50:5 TAG | Triglycerides | M5 darkolivegreen |
| **HMDB0010497*** | C50:6 TAG | Triglycerides | M5 darkolivegreen |
| **HMDB0010517*** | C52:7 TAG | Triglycerides | M5 darkolivegreen |
| **HMDB0010518*** | C54:8 TAG | Triglycerides | M5 darkolivegreen |
| **HMDB0042466*** | C55:3 TAG | Triglycerides | M5 darkolivegreen |
| **HMDB0000562** | creatinine | Carboxylic acids and derivatives | M6 deeppink4 |
| **HMDB0010404** | C22:6 LPC | Lysophosphatidylcholines | M6 deeppink4 |
| **HMDB0011506*** | C18:1 LPE | Lysophosphatidylethanolamines | M6 deeppink4 |
| **HMDB0011507*** | C18:2 LPE | Lysophosphatidylethanolamines | M6 deeppink4 |
| **HMDB0011517** | C20:4 LPE | Lysophosphatidylethanolamines | M6 deeppink4 |
| **HMDB0011526** | C22:6 LPE | Lysophosphatidylethanolamines | M6 deeppink4 |
| **HMDB0000033** | carnosine | NA | M6 deeppink4 |
| **HMDB0000555*** | 3-methyladipate/pimelate | NA | M6 deeppink4 |
| **HMDB0002000** | myristoleic acid | NA | M6 deeppink4 |
| **HMDB0061112** | CMPF | NA | M6 deeppink4 |
| **HMDB0007991*** | C38:6 PC | Phosphatidylcholines | M6 deeppink4 |
| **HMDB0008048*** | C38:4 PC | Phosphatidylcholines | M6 deeppink4 |
| **HMDB0008057*** | C40:6 PC | Phosphatidylcholines | M6 deeppink4 |
| **HMDB0008138*** | C36:4 PC-B | Phosphatidylcholines | M6 deeppink4 |
| **HMDB0008511*** | C40:10 PC | Phosphatidylcholines | M6 deeppink4 |
| **HMDB0008731*** | C40:9 PC | Phosphatidylcholines | M6 deeppink4 |
| **HMDB0012356*** | C34:0 PS | Phosphatidylserines | M6 deeppink4 |
| **HMDB0005392*** | C56:8 TAG | Triglycerides | M6 deeppink4 |
| **HMDB0005406*** | C56:5 TAG | Triglycerides | M6 deeppink4 |
| **HMDB0005413*** | C58:8 TAG | Triglycerides | M6 deeppink4 |
| **HMDB0005448*** | C56:9 TAG | Triglycerides | M6 deeppink4 |
| **HMDB0005456*** | C56:6 TAG | Triglycerides | M6 deeppink4 |
| **HMDB0005458*** | C58:6 TAG | Triglycerides | M6 deeppink4 |
| **HMDB0005462*** | C56:7 TAG | Triglycerides | M6 deeppink4 |
| **HMDB0005463*** | C58:9 TAG | Triglycerides | M6 deeppink4 |
| **HMDB0005471*** | C58:7 TAG | Triglycerides | M6 deeppink4 |
| **HMDB0005478*** | C60:12 TAG | Triglycerides | M6 deeppink4 |

| | | | |
|---|---|---|---|
| **HMDB0010498*** | C54:9 TAG | Triglycerides | M6 deeppink4 |
| **HMDB0010513*** | C56:10 TAG | Triglycerides | M6 deeppink4 |
| **HMDB0010531*** | C58:11 TAG | Triglycerides | M6 deeppink4 |
| **HMDB0000875** | trigonelline | Alkaloids and derivatives | M7 deeppink3 |
| **HMDB0014611** | quinine | Alkaloids and derivatives | M7 deeppink3 |
| **HMDB0000714** | hippurate | Benzene and substituted derivatives | M7 deeppink3 |
| **HMDB0013678** | 4-hydroxyhippurate | Benzenoids | M7 deeppink3 |
| **HMDB0001847** | caffeine | Imidazopyrimidines | M7 deeppink3 |
| **HMDB0000197** | indoleacetate | Indoles and derivatives | M7 deeppink3 |
| **HMDB0000001** | 1-methylhistidine | NA | M7 deeppink3 |
| **HMDB0000099** | cystathionine | NA | M7 deeppink3 |
| **HMDB0000152** | gentisate | NA | M7 deeppink3 |
| **HMDB0000375** | 3-(3-hydroxyphenyl)propionate | NA | M7 deeppink3 |
| **HMDB0000682** | indoxylsulfate | NA | M7 deeppink3 |
| **HMDB0003072** | quinate | NA | M7 deeppink3 |
| **HMDB0003099** | 1-methylurate | NA | M7 deeppink3 |
| **HMDB0032390** | 2-methyl-4,5-benzoxazole | NA | M7 deeppink3 |
| **HMDB0000026** | N-carbamoyl-beta-alanine | Organic acids and derivatives | M7 deeppink3 |
| **HMDB0000479** | methylhistidine | Organic acids and derivatives | M7 deeppink3 |
| **HMDB0000812** | N-acetylaspartic acid | Organic acids and derivatives | M7 deeppink3 |
| **HMDB0001325** | N6,N6,N6-trimethyllysine | Organic acids and derivatives | M7 deeppink3 |
| **HMDB0004827** | proline betaine | Organic acids and derivatives | M7 deeppink3 |
| **HMDB0006344** | phenylacetylglutamine | Organic acids and derivatives | M7 deeppink3 |
| **HMDB0004400** | 5-acetylamino-6-amino-3-methyluracil | Organic nitrogen compounds | M7 deeppink3 |
| **HMDB0001886** | 3-methylxanthine | Organoheterocyclic compounds | M7 deeppink3 |
| **HMDB0001991** | 7-methylxanthine | Organoheterocyclic compounds | M7 deeppink3 |
| **HMDB0002820** | methylimidazole acetic acid | Organoheterocyclic compounds | M7 deeppink3 |
| **HMDB0011103** | 1,7-dimethyluric acid | Organoheterocyclic compounds | M7 deeppink3 |
| **HMDB0000895** | acetylcholine | Organonitrogen compounds | M7 deeppink3 |
| **HMDB0000925** | trimethylamine-N-oxide | Organonitrogen compounds | M7 deeppink3 |
| **HMDB0000462** | allantoin | Azoles | M8 darkviolet |
| **HMDB0010407*** | C16:1 LPC plasmalogen | LPC plasmalogens | M8 darkviolet |
| **HMDB0002815*** | C18:1 LPC | Lysophosphatidylcholines | M8 darkviolet |
| **HMDB0010382** | C16:0 LPC | Lysophosphatidylcholines | M8 darkviolet |
| **HMDB0010383*** | C16:1 LPC | Lysophosphatidylcholines | M8 darkviolet |
| **HMDB0010384** | C18:0 LPC | Lysophosphatidylcholines | M8 darkviolet |

| | | | |
|---|---|---|---|
| **HMDB0010386*** | C18:2 LPC | Lysophosphatidylcholines | M8 darkviolet |
| **HMDB0010387*** | C18:3 LPC | Lysophosphatidylcholines | M8 darkviolet |
| **HMDB0010391*** | C20:1 LPC | Lysophosphatidylcholines | M8 darkviolet |
| **HMDB0010395** | C20:4 LPC | Lysophosphatidylcholines | M8 darkviolet |
| **HMDB0010397** | C20:5 LPC | Lysophosphatidylcholines | M8 darkviolet |
| **HMDB0010403** | C22:5 LPC | Lysophosphatidylcholines | M8 darkviolet |
| **HMDB0011130** | C18:0 LPE | Lysophosphatidylethanolamines | M8 darkviolet |
| **HMDB0011503** | C16:0 LPE | Lysophosphatidylethanolamines | M8 darkviolet |
| **HMDB0011512*** | C20:1 LPE | Lysophosphatidylethanolamines | M8 darkviolet |
| **HMDB0011520** | C22:0 LPE | Lysophosphatidylethanolamines | M8 darkviolet |
| **HMDB0001396** | glycocholate | NA | M8 darkviolet |
| **HMDB0001548** | pentose monophosphate | NA | M8 darkviolet |
| **HMDB0011221*** | C36:5 PC plasmalogen-A | PC plasmalogens | M8 darkviolet |
| **HMDB0011229*** | C38:7 PC plasmalogen | Phosphatidylcholine plasmalogens | M8 darkviolet |
| **HMDB0011394*** | C40:7 PE plasmalogen | Phosphatidylethanolamine plasmalogens | M8 darkviolet |
| **HMDB0011420*** | C38:7 PE plasmalogen | Phosphatidylethanolamine plasmalogens | M8 darkviolet |
| **HMDB0000138** | glycocholate | Steroids and steroid derivatives | M8 darkviolet |
| **HMDB0000896*** | taurodeoxycholate/taurochenodeoxycholate | Steroids and steroid derivatives | M8 darkviolet |
| **HMDB0006347** | C26 carnitine | Carnitines | M9 darkorange |
| **HMDB0004949** | C16:0 Ceramide (d18:1) | Ceramides | M9 darkorange |
| **HMDB0004952** | C22:0 Ceramide (d18:1) | Ceramides | M9 darkorange |
| **HMDB0004953*** | C24:1 Ceramide (d18:1) | Ceramides | M9 darkorange |
| **HMDB0004956** | C24:0 Ceramide (d18:1) | Ceramides | M9 darkorange |
| **HMDB0000067** | cholesterol | NA | M9 darkorange |
| **HMDB0002869** | campesterol | NA | M9 darkorange |
| **HMDB0007973*** | C34:2 PC | Phosphatidylcholines | M9 darkorange |
| **HMDB0007983*** | C36:4 PC-A | Phosphatidylcholines | M9 darkorange |
| **HMDB0008006*** | C34:3 PC | Phosphatidylcholines | M9 darkorange |
| **HMDB0008039*** | C36:2 PC | Phosphatidylcholines | M9 darkorange |
| **HMDB0008105*** | C36:3 PC | Phosphatidylcholines | M9 darkorange |
| **HMDB0008270*** | C38:2 PC | Phosphatidylcholines | M9 darkorange |
| **HMDB0008942*** | C38:2 PE | Phosphatidylethanolamines | M9 darkorange |
| **HMDB0001348** | C18:0 SM | Sphingomyelins | M9 darkorange |
| **HMDB0010169** | C16:0 SM | Sphingomyelins | M9 darkorange |
| **HMDB0012097** | C14:0 SM | Sphingomyelins | M9 darkorange |
| **HMDB0012101*** | C18:1 SM | Sphingomyelins | M9 darkorange |

| | | | |
|---|---|---|---|
| **HMDB0012102** | C20:0 SM | Sphingomyelins | M9 darkorange |
| **HMDB0012104*** | C22:1 SM | Sphingomyelins | M9 darkorange |
| **HMDB0012107*** | C24:1 SM | Sphingomyelins | M9 darkorange |
