# Supplementary material for: Prospective study of circulating metabolomic profiles and breast cancer incidence among predominantly premenopausal women
Source: Br J Cancer. Author manuscript; Available in PMC 2025 Dec 6. (PMC12572396; doi:10.1038/s41416-025-03159-2)
Supplement: Suppl Table 7 [file NIHMS2109610-supplement-Suppl_Table_7.pdf]

Supplemental Table 7. Odds ratios and 95% confidence intervals for associations of WGCNA metabolites (n=381) module scores with breast cancer incidence, stratified by subgroup, Nurses' Health Study II (1996-2012).

Modules	Leading class	Overall	Premenopausal at blood draw	ER+	ER-	BMI<25	BMI≥25
		(1055/1055)	(816/810)	(584/1055)	(126/1055)	(636/579)	(418/475)
		OR (95% CI)	OR (95% CI)	OR (95% CI)	OR (95% CI)	OR (95% CI)	OR (95% CI)
M1 grey	NA	1.02 (0.91-1.13)	0.92 (0.81-1.04)	0.88 (0.76-1.02)	1.14 (0.82-1.59)	0.97 (0.81-1.16)	0.99 (0.76-1.3)
M2 cadetblue1	TAG < 3 DBs	0.97 (0.87-1.08)	1.03 (0.91-1.16)	**0.83 (0.71-0.97)**	0.89 (0.67-1.19)	1 (0.84-1.19)	1.03 (0.81-1.31)
M3 cornflowerblue	Organic acids, PE plasmalogens, PC plasmalogens	0.93 (0.84-1.04)	**1.14 (1.01-1.29)**	0.99 (0.86-1.15)	1.40 (0.97-2.02)	0.95 (0.79-1.14)	1.18 (0.9-1.53)
M4 darkgreen	Carnitines, NA	0.98 (0.87-1.10)	1.01 (0.89-1.14)	0.93 (0.80-1.09)	0.97 (0.69-1.38)	0.97 (0.8-1.16)	0.94 (0.68-1.31)
M5 darkolivegreen	TAG ≥ 3 DBs, amino acids, NA	0.95 (0.85-1.06)	1.06 (0.94-1.20)	**0.80 (0.68-0.94)**	0.95 (0.70-1.28)	0.92 (0.77-1.09)	0.93 (0.71-1.20)
M6 deeppink4	TAG ≥ 3 DBs, PC	1.00 (0.90-1.12)	1.01 (0.90-1.14)	0.97 (0.84-1.12)	0.98 (0.72-1.34)	**0.76 (0.63-0.91)**	0.76 (0.57-1.02)
M7 deeppink3	Organic acids and derivatives	1.03 (0.92-1.15)	1.02 (0.90-1.15)	1.10 (0.94-1.29)	0.89 (0.63-1.27)	1.12 (0.93-1.35)	0.93 (0.69-1.28)
M8 darkviolet	LPC, LPE	1.04 (0.94-1.15)	1.09 (0.96-1.23)	0.96 (0.83-1.11)	1.14 (0.84-1.55)	1.06 (0.88-1.27)	0.96 (0.74-1.25)
M9 darkorange	PC, SM	0.97 (0.86-1.08)	1.02 (0.90-1.16)	**0.82 (0.7-0.96)**	1.03 (0.72-1.49)	1.04 (0.86-1.25)	0.90 (0.67-1.2)

* Model is based on conditional regression model accounting for matching factors and with further adjustment for BMI at age 18, weight change (from age 18 to time of first blood draw), age at menarche, parity and age at first birth, breastfeeding history, family history of breast cancer in a first degree relative, personal history of benign breast disease, physical activity, alcohol intake (by quintile), and oral contraceptive use at blood collection
