# Supplementary material for: Prospective study of circulating metabolomic profiles and breast cancer incidence among predominantly premenopausal women
Source: Br J Cancer. Author manuscript; Available in PMC 2025 Dec 6. (PMC12572396; doi:10.1038/s41416-025-03159-2)
Supplement: Suppl Figure 1 [file NIHMS2109610-supplement-Suppl_Figure_1.pdf]

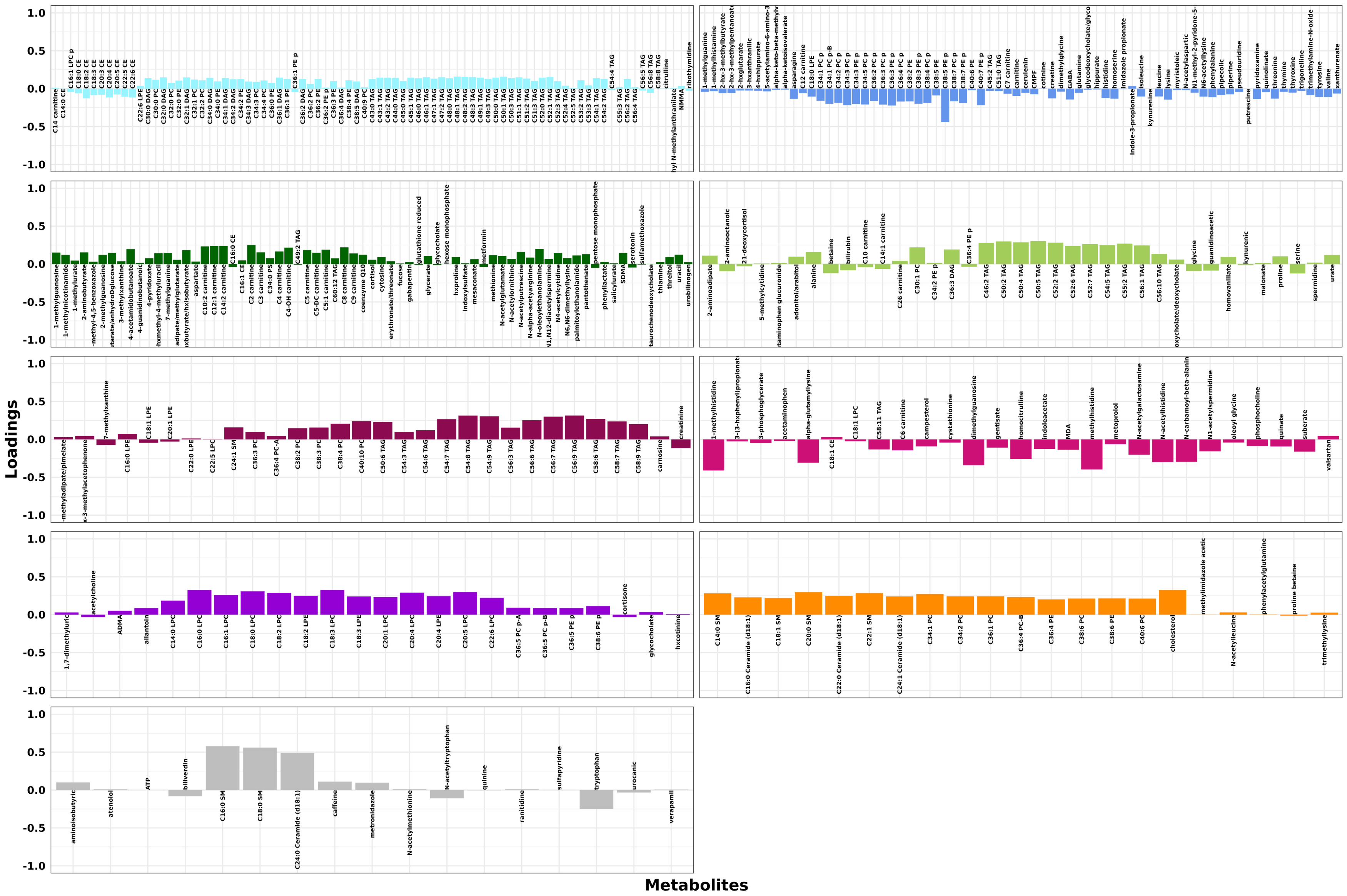

Supplementary Figure 1: WGCNA module loadings for breast cancer. Shown are loadings from modules associated with breast cancer at FDR<0.2. Loadings/module colors correspond to colors in Suppl Table 6.
