# Supplementary material for: Prospective study of circulating metabolomic profiles and breast cancer incidence among predominantly premenopausal women
Source: Br J Cancer. 2025 Sep 4;133(9):1326–34. doi: 10.1038/s41416-025-03159-2 (PMC12572396; doi:10.1038/s41416-025-03159-2)
Supplement: Supplementary file 1 [file 41416_2025_3159_MOESM1_ESM.docx]

**Supplemental Table 1**: Odds ratios and 95% confidence intervals for associations between individual metabolites (per 1 SD increase in metabolite level) and breast cancer incidence, Nurses’ Health Study II (1996-2011)

| **METABOLITE** | **HMDB_ID** | **Class (Broad annotation)** | **OR and 95%CI (model 1)** | **P-value (model 1)** | **OR and 95%CI (model 2)** | **P-value (model 2)** | **OR and 95%CI (model 3)** | **P-value (model 3)** |
| --- | --- | --- | --- | --- | --- | --- | --- | --- |
| **taurodeoxycholate/taurochenodeoxycholate** | HMDB0000896* | alcohols and derivatives | 1.15 (1.05-1.26) | 0.0039 | 1.15 (1.05-1.27) | 0.0035 | 1.15 (1.04-1.28) | 0.0094 |
| **C16:1 CE** | HMDB0000658* | Steroid esters | 0.91 (0.83-0.99) | 0.0303 | 0.92 (0.84-1.01) | 0.0818 | 0.88 (0.79-0.97) | 0.0111 |
| **C34:1 PC** | HMDB0007972* | Glycerophosphocholines | 0.91 (0.83-1) | 0.0395 | 0.93 (0.85-1.02) | 0.1380 | 0.87 (0.78-0.98) | 0.0163 |
| **C34:3 PC** | HMDB0008006* | Glycerophosphocholines | 0.91 (0.83-1) | 0.0394 | 0.92 (0.84-1.01) | 0.0992 | 0.88 (0.79-0.98) | 0.0222 |
| **C32:1 PC** | HMDB0007873* | Glycerophosphocholines | 0.91 (0.83-0.99) | 0.0387 | 0.94 (0.85-1.03) | 0.1992 | 0.88 (0.79-0.98) | 0.0233 |
| **indoxylsulfate** | HMDB0000682 | Arylsulfates | 0.88 (0.81-0.96) | 0.0046 | 0.87 (0.8-0.96) | 0.0031 | 0.9 (0.82-1) | 0.0416 |
| **C34:0 PS** | HMDB0012356* | Glycerophosphocholines | 0.93 (0.85-1.02) | 0.1204 | 0.95 (0.86-1.05) | 0.3005 | 0.89 (0.8-1) | 0.0443 |
| **C58:7 TAG** | HMDB0005471* | Triradylcglycerols (>=3 DB) | 1.1 (1.01-1.2) | 0.0317 | 1.09 (1-1.19) | 0.0613 | 1.11 (1-1.23) | 0.0491 |
| **C14:0 CE** | HMDB0006725 | Steroid esters | 0.97 (0.89-1.05) | 0.4485 | 0.95 (0.87-1.03) | 0.2226 | 0.9 (0.82-1) | 0.0546 |
| **alpha-keto-beta-methylvalerate/alpha-ketoisocaproate** | HMDB0000491* | . | 1.03 (0.94-1.13) | 0.5417 | 1.04 (0.95-1.15) | 0.4012 | 1.11 (0.99-1.24) | 0.0630 |
| **C18:3 CE** | HMDB0010370* | Steroid esters | 0.96 (0.88-1.05) | 0.3493 | 0.93 (0.85-1.02) | 0.1282 | 0.91 (0.82-1.01) | 0.0646 |
| **C36:2 PC plasmalogen** | HMDB0011243* | Glycerophosphocholines | 1.11 (1.02-1.22) | 0.0176 | 1.08 (0.99-1.19) | 0.0986 | 1.1 (0.99-1.23) | 0.0652 |
| **C40:7 PE plasmalogen** | HMDB0011394* | Glycerophosphocholines | 1.12 (1.02-1.23) | 0.0143 | 1.1 (1-1.2) | 0.0524 | 1.1 (0.99-1.23) | 0.0715 |
| **C56:5 TAG** | HMDB0005406* | Triradylcglycerols (>=3 DB) | 1.06 (0.97-1.16) | 0.1906 | 1.07 (0.98-1.18) | 0.1157 | 1.1 (0.99-1.22) | 0.0731 |
| **N-acetylglutamate** | HMDB0001138 | Amino acids, peptides, and analogues | 1.05 (0.96-1.15) | 0.2957 | 1.08 (0.98-1.18) | 0.1310 | 1.1 (0.99-1.22) | 0.0921 |
| **C56:4 TAG** | HMDB0005398* | Triradylcglycerols (>=3 DB) | 1.05 (0.96-1.14) | 0.3082 | 1.05 (0.97-1.15) | 0.2425 | 1.09 (0.99-1.2) | 0.0940 |
| **kynurenine** | HMDB0000684 | Carbonyl compounds | 0.88 (0.8-0.97) | 0.0092 | 0.89 (0.81-0.99) | 0.0277 | 0.91 (0.81-1.02) | 0.1010 |
| **C34:3 PC plasmalogen** | HMDB0011211* | Glycerophosphocholines | 1.1 (1.01-1.2) | 0.0351 | 1.06 (0.97-1.17) | 0.2009 | 1.09 (0.98-1.21) | 0.1097 |
| **C34:1 PC plasmalogen-B** | HMDB0011239* | Glycerophosphocholines | 1.12 (1.02-1.22) | 0.0159 | 1.09 (0.99-1.2) | 0.0851 | 1.09 (0.98-1.22) | 0.1113 |
| **C36:4 DAG** | HMDB0007248* | Lineolic acids and derivatives | 1.01 (0.93-1.1) | 0.7507 | 1.03 (0.94-1.12) | 0.5202 | 1.09 (0.98-1.2) | 0.1149 |
| **indoleacetate** | HMDB0000197 | Pyridinecarboxylic acids and derivatives | 0.98 (0.9-1.08) | 0.7290 | 0.97 (0.88-1.06) | 0.4669 | 0.92 (0.84-1.02) | 0.1206 |
| **2-aminobutyrate** | HMDB0000650 | Amino acids, peptides, and analogues | 1.02 (0.93-1.12) | 0.6336 | 1.04 (0.95-1.14) | 0.4011 | 1.09 (0.98-1.2) | 0.1226 |
| **C36:3 PC plasmalogen** | HMDB0011244* | Glycerophosphocholines | 1.1 (1.01-1.2) | 0.0344 | 1.05 (0.96-1.16) | 0.2754 | 1.09 (0.98-1.21) | 0.1253 |
| **C36:1 PC** | HMDB0008038* | Glycerophosphocholines | 0.93 (0.85-1.02) | 0.1327 | 0.95 (0.87-1.04) | 0.2817 | 0.92 (0.83-1.02) | 0.1255 |
| **C52:1 TAG** | HMDB0005367* | Triradylcglycerols  (<3 DB) | 0.89 (0.82-0.98) | 0.0129 | 0.93 (0.84-1.02) | 0.1347 | 0.92 (0.82-1.03) | 0.1286 |
| **C54:5 TAG** | HMDB0005385* | Triradylcglycerols (>=3 DB) | 0.92 (0.84-1) | 0.0625 | 0.95 (0.87-1.05) | 0.3343 | 0.92 (0.83-1.03) | 0.1328 |
| **C30:1 PC** | HMDB0007870* | Glycerophosphocholines | 0.93 (0.85-1.02) | 0.1219 | 0.96 (0.87-1.05) | 0.3350 | 0.92 (0.83-1.02) | 0.1365 |
| **C36:3 DAG** | HMDB0007219* | Lineolic acids and derivatives | 0.99 (0.91-1.08) | 0.8300 | 1.02 (0.93-1.11) | 0.6924 | 1.08 (0.97-1.19) | 0.1531 |
| **C46:0 TAG** | HMDB0010411* | Triradylcglycerols  (<3 DB) | 0.93 (0.85-1.01) | 0.0789 | 0.95 (0.86-1.03) | 0.2231 | 0.93 (0.84-1.03) | 0.1557 |
| **palmitoylethanolamide** | HMDB0002100 | Carboximidic acids | 1.02 (0.92-1.12) | 0.7497 | 1.04 (0.94-1.16) | 0.4478 | 1.09 (0.97-1.22) | 0.1612 |
| **2-aminoadipate** | HMDB0000510 | Amino acids, peptides, and analogues | 0.9 (0.81-0.99) | 0.0337 | 0.93 (0.84-1.04) | 0.1971 | 0.92 (0.82-1.03) | 0.1643 |
| **C34:4 PC** | HMDB0007883* | Glycerophosphocholines | 0.93 (0.85-1.02) | 0.1382 | 0.96 (0.87-1.05) | 0.3676 | 0.93 (0.83-1.03) | 0.1701 |
| **C55:3 TAG** | HMDB0042466* | Triradylcglycerols (>=3 DB) | 1.05 (0.96-1.14) | 0.3077 | 1.04 (0.95-1.13) | 0.4098 | 1.07 (0.97-1.18) | 0.1736 |
| **2-hydroxyglutarate** | HMDB0000694 | Fatty acids and conjugates | 1.06 (0.96-1.18) | 0.2597 | 1.08 (0.97-1.2) | 0.1562 | 1.09 (0.96-1.22) | 0.1745 |
| **C52:7 TAG** | HMDB0010517* | Triradylcglycerols (>=3 DB) | 0.93 (0.85-1.02) | 0.1272 | 0.96 (0.87-1.05) | 0.3279 | 0.93 (0.84-1.03) | 0.1795 |
| **C50:6 TAG** | HMDB0010497* | Triradylcglycerols (>=3 DB) | 0.92 (0.84-1) | 0.0585 | 0.94 (0.86-1.03) | 0.2125 | 0.93 (0.84-1.03) | 0.1811 |
| **C34:1 PC plasmalogen** | HMDB0011208* | Glycerophosphocholines | 1.11 (1.01-1.21) | 0.0220 | 1.09 (0.99-1.19) | 0.0752 | 1.07 (0.97-1.19) | 0.1826 |
| **C18:0 LPC** | HMDB0010384 | Glycerophosphocholines | 1.06 (0.97-1.17) | 0.1778 | 1.05 (0.96-1.15) | 0.3064 | 1.07 (0.97-1.19) | 0.1828 |
| **C50:0 TAG** | HMDB0005357* | Triradylcglycerols  (<3 DB) | 0.92 (0.85-1.01) | 0.0722 | 0.95 (0.87-1.04) | 0.2815 | 0.93 (0.84-1.04) | 0.1856 |
| **C54:4 TAG** | HMDB0005370* | Triradylcglycerols (>=3 DB) | 1.03 (0.94-1.12) | 0.5132 | 1.02 (0.94-1.12) | 0.6168 | 1.07 (0.97-1.18) | 0.1946 |
| **mesaconate** | HMDB0000749 | . | 1.06 (0.96-1.16) | 0.2608 | 1.06 (0.96-1.17) | 0.2269 | 1.07 (0.96-1.19) | 0.1951 |
| **alpha-ketoisovalerate** | HMDB0000019 | Short-chain keto acids and derivatives | 1.03 (0.94-1.14) | 0.5243 | 1.06 (0.95-1.17) | 0.2844 | 1.08 (0.96-1.21) | 0.2005 |
| **indole-3-propionate** | HMDB0002302 | Indolyl carboxylic acids and derivatives | 1.07 (0.98-1.17) | 0.1180 | 1.05 (0.96-1.14) | 0.3363 | 1.07 (0.96-1.18) | 0.2014 |
| **C48:0 TAG** | HMDB0005356* | Triradylcglycerols  (<3 DB) | 0.93 (0.85-1.02) | 0.1152 | 0.96 (0.87-1.05) | 0.3637 | 0.93 (0.84-1.04) | 0.2044 |
| **C38:7 PE plasmalogen** | HMDB0011420* | Glycerophosphocholines | 1.08 (0.99-1.19) | 0.0849 | 1.08 (0.99-1.19) | 0.0856 | 1.07 (0.96-1.2) | 0.2061 |
| **xanthurenate** | HMDB0000881 | Quinoline carboxylic acids | 0.95 (0.87-1.05) | 0.3102 | 0.97 (0.88-1.06) | 0.4940 | 0.93 (0.84-1.04) | 0.2085 |
| **C50:5 TAG** | HMDB0010471* | Triradylcglycerols (>=3 DB) | 0.92 (0.84-1) | 0.0499 | 0.94 (0.86-1.03) | 0.2094 | 0.94 (0.84-1.04) | 0.2095 |
| **C18:0 LPE** | HMDB0011130 | Glycerophosphocholines | 1.07 (0.98-1.18) | 0.1305 | 1.06 (0.97-1.16) | 0.2037 | 1.07 (0.96-1.19) | 0.2105 |
| **C22:0 LPE** | HMDB0011520 | Glycerophosphocholines | 1.1 (1.01-1.21) | 0.0327 | 1.05 (0.95-1.16) | 0.3001 | 1.07 (0.96-1.2) | 0.2133 |
| **C43:1 TAG** | HMDB0042098* | Triradylcglycerols  (<3 DB) | 0.93 (0.85-1.02) | 0.1112 | 0.95 (0.87-1.04) | 0.2948 | 0.94 (0.85-1.04) | 0.2148 |
| **C46:1 TAG** | HMDB0010412* | Triradylcglycerols  (<3 DB) | 0.91 (0.83-0.99) | 0.0318 | 0.93 (0.85-1.02) | 0.1361 | 0.94 (0.85-1.04) | 0.2215 |
| **2-hydroxy-3-methylbutyrate** | HMDB0000407 | Fatty acids and conjugates | 0.89 (0.82-0.98) | 0.0153 | 0.91 (0.83-1) | 0.0531 | 0.94 (0.84-1.04) | 0.2260 |
| **C36:5 PE plasmalogen** | HMDB0011410* | Glycerophosphocholines | 1.03 (0.94-1.13) | 0.4819 | 1.04 (0.95-1.14) | 0.3797 | 1.07 (0.96-1.18) | 0.2287 |
| **C22:5 CE** | HMDB0010375* | Steroid esters | 1.01 (0.92-1.11) | 0.8267 | 0.96 (0.87-1.07) | 0.4710 | 0.93 (0.83-1.05) | 0.2314 |
| **C56:7 TAG** | HMDB0005462* | Triradylcglycerols (>=3 DB) | 1.05 (0.96-1.15) | 0.2824 | 1.06 (0.97-1.16) | 0.2071 | 1.06 (0.96-1.18) | 0.2383 |
| **C54:9 TAG** | HMDB0010498* | Triradylcglycerols (>=3 DB) | 0.97 (0.89-1.07) | 0.5723 | 0.99 (0.9-1.08) | 0.7661 | 0.94 (0.85-1.04) | 0.2471 |
| **C48:1 TAG** | HMDB0005359* | Triradylcglycerols  (<3 DB) | 0.92 (0.84-1) | 0.0537 | 0.95 (0.87-1.04) | 0.2840 | 0.94 (0.85-1.04) | 0.2483 |
| **C50:2 TAG** | HMDB0005377* | Triradylcglycerols  (<3 DB) | 0.91 (0.83-0.99) | 0.0321 | 0.95 (0.86-1.05) | 0.2965 | 0.94 (0.84-1.05) | 0.2484 |
| **C50:1 TAG** | HMDB0005360* | Triradylcglycerols  (<3 DB) | 0.91 (0.83-1) | 0.0415 | 0.95 (0.86-1.05) | 0.3296 | 0.94 (0.84-1.05) | 0.2487 |
| **C58:6 TAG** | HMDB0005458* | Triradylcglycerols (>=3 DB) | 1.05 (0.96-1.15) | 0.3085 | 1.05 (0.96-1.15) | 0.2642 | 1.06 (0.96-1.18) | 0.2490 |
| **C22:6 CE** | HMDB0006733 | Steroid esters | 1.11 (1.02-1.21) | 0.0159 | 1.08 (0.99-1.18) | 0.1009 | 1.06 (0.96-1.18) | 0.2500 |
| **campesterol** | HMDB0002869 | Ergostane steroids | 0.94 (0.85-1.03) | 0.1891 | 0.96 (0.87-1.06) | 0.4355 | 0.94 (0.84-1.05) | 0.2509 |
| **cholesterol** | HMDB0000067 | Cholestane steroids | 0.94 (0.85-1.03) | 0.1700 | 0.96 (0.87-1.06) | 0.4597 | 0.94 (0.84-1.05) | 0.2626 |
| **C58:9 TAG** | HMDB0005463* | Triradylcglycerols (>=3 DB) | 1.11 (1.02-1.21) | 0.0216 | 1.09 (0.99-1.19) | 0.0744 | 1.06 (0.96-1.18) | 0.2634 |
| **C48:2 TAG** | HMDB0005376* | Triradylcglycerols  (<3 DB) | 0.91 (0.84-0.99) | 0.0374 | 0.94 (0.86-1.03) | 0.2075 | 0.94 (0.85-1.05) | 0.2743 |
| **C30:0 PC** | HMDB0007869* | Glycerophosphocholines | 0.97 (0.89-1.06) | 0.4933 | 0.98 (0.9-1.08) | 0.7316 | 0.95 (0.85-1.05) | 0.2784 |
| **C53:3 TAG** | HMDB0043058* | Triradylcglycerols (>=3 DB) | 0.99 (0.91-1.08) | 0.8630 | 1 (0.91-1.09) | 0.9408 | 1.06 (0.96-1.17) | 0.2796 |
| **C20:5 CE** | HMDB0006731 | Steroid esters | 1.01 (0.92-1.1) | 0.8982 | 0.99 (0.91-1.09) | 0.8864 | 0.95 (0.85-1.05) | 0.2828 |
| **C34:5 PC plasmalogen** | HMDB0011214* | Glycerophosphocholines | 1.04 (0.95-1.14) | 0.3633 | 1.04 (0.95-1.13) | 0.4499 | 1.06 (0.96-1.17) | 0.2829 |
| **C34:2 PC plasmalogen** | HMDB0011210* | Glycerophosphocholines | 1.11 (1.02-1.22) | 0.0151 | 1.08 (0.98-1.19) | 0.1030 | 1.06 (0.95-1.18) | 0.2841 |
| **C38:5 PE plasmalogen** | HMDB0011386* | Glycerophosphocholines | 1.03 (0.95-1.13) | 0.4668 | 1.03 (0.94-1.13) | 0.5144 | 1.06 (0.95-1.17) | 0.2851 |
| **C22:6 LPE** | HMDB0011526 | Glycerophosphocholines | 1.09 (0.99-1.2) | 0.0675 | 1.09 (0.99-1.2) | 0.0649 | 1.06 (0.95-1.18) | 0.2897 |
| **C54:8 TAG** | HMDB0010518* | Triradylcglycerols (>=3 DB) | 0.97 (0.89-1.06) | 0.4843 | 0.98 (0.9-1.07) | 0.6486 | 0.95 (0.86-1.05) | 0.2925 |
| **C20:4 LPE** | HMDB0011517 | Glycerophosphocholines | 1.05 (0.96-1.15) | 0.2769 | 1.05 (0.96-1.15) | 0.2643 | 1.06 (0.95-1.17) | 0.2947 |
| **C22:6 LPC** | HMDB0010404 | Glycerophosphocholines | 1.11 (1.02-1.22) | 0.0181 | 1.09 (0.99-1.2) | 0.0716 | 1.06 (0.95-1.18) | 0.3018 |
| **C54:3 TAG** | HMDB0005405* | Triradylcglycerols (>=3 DB) | 1 (0.92-1.09) | 0.9960 | 1.02 (0.93-1.11) | 0.6801 | 1.05 (0.95-1.17) | 0.3055 |
| **C56:8 TAG** | HMDB0005392* | Triradylcglycerols (>=3 DB) | 1.07 (0.98-1.16) | 0.1368 | 1.06 (0.97-1.16) | 0.1954 | 1.05 (0.95-1.16) | 0.3074 |
| **C36:1 PE plasmalogen** | HMDB0009016* | Glycerophosphocholines | 1.09 (1-1.19) | 0.0581 | 1.05 (0.96-1.15) | 0.3098 | 1.06 (0.95-1.17) | 0.3125 |
| **C38:4 PC plasmalogen** | HMDB0011252* | Glycerophosphocholines | 1.04 (0.95-1.14) | 0.3452 | 1.03 (0.94-1.13) | 0.5596 | 1.06 (0.95-1.17) | 0.3159 |
| **C56:10 TAG** | HMDB0010513* | Triradylcglycerols (>=3 DB) | 0.99 (0.9-1.08) | 0.7811 | 1 (0.91-1.09) | 0.9372 | 0.95 (0.85-1.05) | 0.3190 |
| **quinolinate** | HMDB0000232 | Pyrimidines and pyrimidine derivatives | 0.93 (0.85-1.01) | 0.0966 | 0.96 (0.87-1.05) | 0.3755 | 0.95 (0.85-1.05) | 0.3266 |
| **C48:3 TAG** | HMDB0005432* | Triradylcglycerols (>=3 DB) | 0.91 (0.83-0.99) | 0.0267 | 0.94 (0.85-1.03) | 0.1572 | 0.95 (0.86-1.05) | 0.3271 |
| **hexose monophosphate** | HMDB0001401* | . | 1.06 (0.96-1.16) | 0.2452 | 1.03 (0.94-1.14) | 0.5188 | 1.06 (0.95-1.18) | 0.3316 |
| **C44:0 TAG** | HMDB0042063* | Triradylcglycerols  (<3 DB) | 0.93 (0.85-1.01) | 0.0762 | 0.95 (0.87-1.03) | 0.2240 | 0.95 (0.86-1.05) | 0.3350 |
| **C52:0 TAG** | HMDB0005365* | Triradylcglycerols  (<3 DB) | 0.93 (0.85-1.01) | 0.0907 | 0.95 (0.87-1.05) | 0.3170 | 0.95 (0.85-1.06) | 0.3365 |
| **C36:4 PC plasmalogen** | HMDB0011310* | Glycerophosphocholines | 1.1 (1.01-1.2) | 0.0360 | 1.07 (0.97-1.18) | 0.1705 | 1.05 (0.95-1.17) | 0.3381 |
| **4-pyridoxate** | HMDB0000017 | Pyridinecarboxylic acids and derivatives | 0.98 (0.9-1.07) | 0.6366 | 0.98 (0.9-1.07) | 0.6755 | 0.95 (0.86-1.05) | 0.3394 |
| **C46:2 TAG** | HMDB0010419* | Triradylcglycerols  (<3 DB) | 0.92 (0.84-1) | 0.0526 | 0.94 (0.86-1.03) | 0.2162 | 0.95 (0.86-1.05) | 0.3411 |
| **C51:3 TAG** | HMDB0011701* | Triradylcglycerols (>=3 DB) | 0.97 (0.89-1.06) | 0.5254 | 1 (0.91-1.09) | 0.9376 | 1.05 (0.95-1.17) | 0.3445 |
| **C45:1 TAG** | HMDB0042099* | Triradylcglycerols  (<3 DB) | 0.93 (0.86-1.02) | 0.1151 | 0.95 (0.87-1.04) | 0.3030 | 0.95 (0.86-1.05) | 0.3454 |
| **C51:1 TAG** | HMDB0042104* | Triradylcglycerols  (<3 DB) | 0.93 (0.85-1.01) | 0.0851 | 0.96 (0.87-1.05) | 0.3585 | 0.95 (0.86-1.06) | 0.3572 |
| **C51:2 TAG** | HMDB0005362* | Triradylcglycerols  (<3 DB) | 0.92 (0.84-1) | 0.0602 | 0.95 (0.87-1.04) | 0.2493 | 0.95 (0.86-1.06) | 0.3621 |
| **C52:4 TAG** | HMDB0005363* | Triradylcglycerols (>=3 DB) | 0.99 (0.91-1.07) | 0.7570 | 1.01 (0.92-1.1) | 0.8787 | 1.05 (0.95-1.16) | 0.3630 |
| **C52:6 TAG** | HMDB0005436* | Triradylcglycerols (>=3 DB) | 0.94 (0.86-1.03) | 0.1724 | 0.96 (0.88-1.05) | 0.4008 | 0.95 (0.86-1.06) | 0.3667 |
| **C20:3 CE** | HMDB0006736* | Steroid esters | 0.99 (0.91-1.09) | 0.9028 | 0.98 (0.9-1.07) | 0.6943 | 0.96 (0.87-1.05) | 0.3683 |
| **C38:2 PE** | HMDB0008942* | Glycerophosphocholines | 1.06 (0.97-1.15) | 0.1850 | 1.04 (0.95-1.13) | 0.4427 | 1.05 (0.95-1.16) | 0.3688 |
| **CMPF** | HMDB0061112 | Fatty acids and conjugates | 1.09 (0.99-1.19) | 0.0659 | 1.07 (0.98-1.18) | 0.1288 | 1.05 (0.94-1.17) | 0.3754 |
| **C60:12 TAG** | HMDB0005478* | Triradylcglycerols (>=3 DB) | 1.09 (1-1.19) | 0.0596 | 1.08 (0.99-1.18) | 0.0973 | 1.05 (0.94-1.17) | 0.3760 |
| **C51:0 TAG** | HMDB0031106* | Triradylcglycerols  (<3 DB) | 0.95 (0.87-1.03) | 0.2250 | 0.97 (0.88-1.06) | 0.4580 | 0.96 (0.86-1.06) | 0.3835 |
| **C38:3 PE plasmalogen** | HMDB0011384* | Glycerophosphocholines | 1.06 (0.97-1.15) | 0.2229 | 1.02 (0.93-1.11) | 0.7338 | 1.05 (0.94-1.16) | 0.3943 |
| **C34:3 DAG** | HMDB0007132* | Lineolic acids and derivatives | 0.96 (0.88-1.05) | 0.4105 | 1 (0.91-1.1) | 0.9565 | 1.05 (0.94-1.16) | 0.3958 |
| **C36:4 PC-A** | HMDB0007983* | Glycerophosphocholines | 1 (0.91-1.09) | 0.9504 | 0.98 (0.9-1.07) | 0.6908 | 0.96 (0.87-1.06) | 0.3984 |
| **C43:2 TAG** | HMDB0043169* | Triradylcglycerols  (<3 DB) | 0.95 (0.87-1.04) | 0.2631 | 0.98 (0.89-1.07) | 0.6322 | 0.96 (0.86-1.06) | 0.4031 |
| **C38:3 PC** | HMDB0008047* | Glycerophosphocholines | 0.92 (0.84-1.01) | 0.0819 | 0.96 (0.87-1.06) | 0.4736 | 0.95 (0.85-1.07) | 0.4043 |
| **MDA** | HMDB0006112 | Carbonyl compounds | 1.04 (0.93-1.16) | 0.4764 | 1.05 (0.94-1.17) | 0.4013 | 1.05 (0.93-1.19) | 0.4063 |
| **C56:3 TAG** | HMDB0005410* | Triradylcglycerols (>=3 DB) | 0.99 (0.9-1.09) | 0.8447 | 1.02 (0.93-1.12) | 0.6731 | 1.05 (0.94-1.16) | 0.4086 |
| **phenyllactate** | HMDB0000779 | . | 1.03 (0.93-1.14) | 0.5378 | 1.02 (0.92-1.13) | 0.6606 | 1.05 (0.93-1.18) | 0.4103 |
| **C38:2 PC** | HMDB0008270* | Glycerophosphocholines | 0.95 (0.87-1.05) | 0.3077 | 0.98 (0.89-1.07) | 0.6353 | 0.96 (0.86-1.06) | 0.4110 |
| **aspartate** | HMDB0000191 | Indolyl carboxylic acids and derivatives | 1.01 (0.92-1.11) | 0.7697 | 1.04 (0.95-1.15) | 0.3975 | 1.04 (0.94-1.16) | 0.4274 |
| **C36:4 PC-B** | HMDB0008138* | Glycerophosphocholines | 0.95 (0.87-1.05) | 0.3216 | 0.98 (0.89-1.08) | 0.7264 | 0.96 (0.85-1.07) | 0.4309 |
| **3-hydroxymethylglutarate/anhydroDglucose** | HMDB0000355 | . | 1.04 (0.95-1.15) | 0.4038 | 1.05 (0.95-1.16) | 0.3598 | 1.05 (0.94-1.17) | 0.4309 |
| **glycerate** | HMDB0000139 | Carbohydrates and carbohydrate conjugates | 1.01 (0.92-1.12) | 0.8051 | 1.03 (0.93-1.14) | 0.6047 | 1.05 (0.93-1.17) | 0.4433 |
| **C47:2 TAG** | HMDB0042076* | Triradylcglycerols  (<3 DB) | 0.94 (0.86-1.02) | 0.1456 | 0.96 (0.88-1.05) | 0.3998 | 0.96 (0.87-1.06) | 0.4442 |
| **C45:2 TAG** | HMDB0043170* | Triradylcglycerols  (<3 DB) | 0.95 (0.87-1.03) | 0.2103 | 0.97 (0.89-1.06) | 0.5375 | 0.96 (0.87-1.06) | 0.4502 |
| **threitol** | HMDB0004136 | Carbohydrates and carbohydrate conjugates | 1.06 (0.97-1.16) | 0.2099 | 1.06 (0.97-1.16) | 0.2103 | 1.04 (0.94-1.15) | 0.4504 |
| **2-hydroxy-3-methylpentanoate/hydroxyisocaproate** | HMDB0000317* | Fatty acids and conjugates | 0.96 (0.88-1.05) | 0.3730 | 0.98 (0.89-1.08) | 0.6931 | 1.04 (0.94-1.16) | 0.4571 |
| **C16:0 LPE** | HMDB0011503 | Glycerophosphocholines | 1.07 (0.98-1.17) | 0.1319 | 1.06 (0.96-1.16) | 0.2363 | 1.04 (0.94-1.16) | 0.4614 |
| **C36:2 PE plasmalogen** | HMDB0009082* | Glycerophosphocholines | 1.03 (0.94-1.12) | 0.5513 | 1.02 (0.93-1.12) | 0.6777 | 1.04 (0.94-1.15) | 0.4699 |
| **C34:3 PE plasmalogen** | HMDB0011343* | Glycerophosphocholines | 1.02 (0.94-1.11) | 0.6425 | 1.01 (0.92-1.1) | 0.8638 | 1.04 (0.94-1.15) | 0.4702 |
| **C18:2 LPC** | HMDB0010386* | Glycerophosphocholines | 1.09 (1-1.2) | 0.0517 | 1.05 (0.96-1.16) | 0.2844 | 1.04 (0.94-1.16) | 0.4737 |
| **C16:1 LPC** | HMDB0010383* | Glycerophosphocholines | 0.96 (0.88-1.05) | 0.3836 | 0.97 (0.89-1.06) | 0.5248 | 0.96 (0.87-1.07) | 0.4797 |
| **C18:1 LPE** | HMDB0011506* | Glycerophosphocholines | 1.05 (0.96-1.15) | 0.2612 | 1.04 (0.95-1.14) | 0.3908 | 1.04 (0.94-1.15) | 0.4803 |
| **2-aminoheptanoate** | HMDB0094649 | . | 1.01 (0.92-1.1) | 0.8259 | 1.03 (0.94-1.13) | 0.4602 | 1.04 (0.94-1.15) | 0.4921 |
| **C56:6 TAG** | HMDB0005456* | Triradylcglycerols (>=3 DB) | 1.03 (0.94-1.12) | 0.5664 | 1.03 (0.94-1.13) | 0.4824 | 1.04 (0.94-1.15) | 0.4959 |
| **C54:7 TAG** | HMDB0005447* | Triradylcglycerols (>=3 DB) | 0.98 (0.9-1.07) | 0.6691 | 0.98 (0.9-1.07) | 0.6484 | 0.97 (0.88-1.07) | 0.5091 |
| **C18:1 SM** | HMDB0012101* | Phosphosphingolipids | 0.99 (0.9-1.08) | 0.7951 | 1.03 (0.93-1.13) | 0.5895 | 1.04 (0.93-1.16) | 0.5141 |
| **C36:5 PC plasmalogen-B** | HMDB0011220* | Glycerophosphocholines | 1.01 (0.93-1.11) | 0.7739 | 1.01 (0.92-1.11) | 0.8270 | 1.03 (0.93-1.15) | 0.5142 |
| **C36:3 PE** | HMDB0009060* | Glycerophosphocholines | 1.01 (0.93-1.11) | 0.7487 | 1.03 (0.94-1.12) | 0.5583 | 1.03 (0.93-1.14) | 0.5182 |
| **C18:2 LPE** | HMDB0011507* | Glycerophosphocholines | 1.06 (0.97-1.16) | 0.2006 | 1.04 (0.95-1.14) | 0.3578 | 1.03 (0.93-1.14) | 0.5260 |
| **gentisate** | HMDB0000152 | Carbohydrates and carbohydrate conjugates | 0.95 (0.84-1.09) | 0.4772 | 0.95 (0.84-1.09) | 0.4675 | 0.95 (0.82-1.11) | 0.5283 |
| **C34:2 PE plasmalogen** | HMDB0008952* | Glycerophosphocholines | 1.03 (0.95-1.13) | 0.4529 | 1.03 (0.94-1.12) | 0.5727 | 1.03 (0.93-1.14) | 0.5392 |
| **C38:6 PE plasmalogen** | HMDB0011387* | Glycerophosphocholines | 1.02 (0.93-1.11) | 0.7356 | 1.01 (0.93-1.11) | 0.7763 | 1.03 (0.93-1.14) | 0.5406 |
| **homovanillate** | HMDB0000118 | Methoxyphenols | 1 (0.92-1.09) | 0.9917 | 1.02 (0.94-1.12) | 0.5984 | 1.03 (0.93-1.15) | 0.5409 |
| **C18:0 SM** | HMDB0001348 | Phosphosphingolipids | 1.02 (0.93-1.12) | 0.6338 | 1.05 (0.96-1.16) | 0.2709 | 1.03 (0.93-1.15) | 0.5441 |
| **C18:1 CE** | HMDB0000918* | Steroid esters | 1.04 (0.95-1.14) | 0.3772 | 1 (0.91-1.1) | 0.9642 | 0.97 (0.87-1.08) | 0.5493 |
| **coenzyme Q10** | HMDB0001072 | . | 0.96 (0.88-1.05) | 0.3326 | 0.97 (0.89-1.06) | 0.5475 | 0.97 (0.87-1.08) | 0.5496 |
| **C32:0 PC** | HMDB0007871* | Glycerophosphocholines | 1.01 (0.93-1.11) | 0.7679 | 1.02 (0.93-1.12) | 0.6665 | 0.97 (0.87-1.08) | 0.5508 |
| **C32:0 DAG** | HMDB0007098* | Diacylglycerols | 0.94 (0.86-1.02) | 0.1479 | 0.97 (0.88-1.07) | 0.5923 | 0.97 (0.87-1.08) | 0.5537 |
| **C22:0 Ceramide (d18:1)** | HMDB0004952 | Ceramides | 0.92 (0.84-1.01) | 0.0667 | 0.95 (0.87-1.05) | 0.3143 | 0.97 (0.88-1.07) | 0.5629 |
| **C40:10 PC** | HMDB0008511* | Glycerophosphocholines | 1 (0.91-1.1) | 0.9949 | 1 (0.91-1.09) | 0.9433 | 0.97 (0.87-1.08) | 0.5632 |
| **C43:0 TAG** | HMDB0042062* | Triradylcglycerols  (<3 DB) | 0.96 (0.88-1.05) | 0.3587 | 0.97 (0.89-1.07) | 0.5666 | 0.97 (0.88-1.07) | 0.5634 |
| **C20:5 LPC** | HMDB0010397 | Glycerophosphocholines | 1.09 (1-1.19) | 0.0641 | 1.05 (0.96-1.16) | 0.3031 | 1.03 (0.93-1.15) | 0.5697 |
| **C47:1 TAG** | HMDB0042100* | Triradylcglycerols  (<3 DB) | 0.96 (0.88-1.04) | 0.2997 | 0.97 (0.89-1.06) | 0.5592 | 0.97 (0.88-1.07) | 0.5699 |
| **C32:2 PC** | HMDB0007874* | Glycerophosphocholines | 0.97 (0.88-1.06) | 0.4714 | 0.99 (0.9-1.09) | 0.8522 | 0.97 (0.88-1.08) | 0.5726 |
| **alpha-hydroxybutyrate/beta-hydroxybutyrate/hydroxyisobutyrate** | HMDB0000008* | Alpha hydroxy acids and derivatives | 0.97 (0.88-1.06) | 0.4917 | 1.01 (0.91-1.11) | 0.8740 | 1.03 (0.93-1.15) | 0.5763 |
| **C58:11 TAG** | HMDB0010531* | Triradylcglycerols (>=3 DB) | 1.01 (0.92-1.11) | 0.7989 | 1.02 (0.93-1.12) | 0.7050 | 0.97 (0.87-1.08) | 0.5847 |
| **C18:1 LPC** | HMDB0002815* | Glycerophosphocholines | 1.05 (0.96-1.15) | 0.2711 | 1.02 (0.93-1.12) | 0.6529 | 1.03 (0.93-1.14) | 0.5939 |
| **C16:0 SM** | HMDB0010169 | Phosphosphingolipids | 1.06 (0.97-1.16) | 0.2236 | 1.05 (0.96-1.15) | 0.2783 | 1.03 (0.93-1.14) | 0.5956 |
| **C36:4 PE plasmalogen** | HMDB0011442* | Glycerophosphocholines | 1.02 (0.93-1.11) | 0.7250 | 1 (0.91-1.09) | 0.9711 | 1.03 (0.93-1.14) | 0.6003 |
| **C14:0 SM** | HMDB0012097 | Phosphosphingolipids | 0.97 (0.88-1.06) | 0.4651 | 0.99 (0.9-1.08) | 0.7998 | 0.97 (0.88-1.08) | 0.6108 |
| **C32:0 PE** | HMDB0008923* | Glycerophosphocholines | 0.99 (0.91-1.08) | 0.8405 | 1 (0.91-1.09) | 0.9662 | 0.98 (0.88-1.08) | 0.6180 |
| **C36:3 PC** | HMDB0008105* | Glycerophosphocholines | 0.95 (0.87-1.04) | 0.3021 | 0.98 (0.89-1.07) | 0.6083 | 0.97 (0.88-1.08) | 0.6204 |
| **C36:0 PE** | HMDB0008991* | Glycerophosphocholines | 1 (0.92-1.09) | 0.9899 | 0.99 (0.91-1.08) | 0.8623 | 0.98 (0.88-1.08) | 0.6230 |
| **malonate** | HMDB0000691 | Amino acids, peptides, and analogues | 0.95 (0.86-1.05) | 0.3446 | 0.95 (0.86-1.06) | 0.3666 | 0.97 (0.87-1.09) | 0.6232 |
| **C52:3 TAG** | HMDB0005384* | Triradylcglycerols (>=3 DB) | 0.94 (0.86-1.03) | 0.1936 | 0.99 (0.9-1.08) | 0.7862 | 1.03 (0.92-1.14) | 0.6265 |
| **C34:0 PE** | HMDB0008925* | Glycerophosphocholines | 1 (0.91-1.09) | 0.9362 | 1 (0.92-1.09) | 0.9790 | 0.98 (0.88-1.08) | 0.6277 |
| **C54:1 TAG** | HMDB0005395* | Triradylcglycerols  (<3 DB) | 0.93 (0.85-1.01) | 0.0990 | 0.96 (0.87-1.05) | 0.3741 | 0.97 (0.88-1.08) | 0.6344 |
| **C36:1 DAG** | HMDB0007216* | Diacylglycerols | 0.91 (0.84-1) | 0.0470 | 0.95 (0.86-1.05) | 0.2954 | 0.97 (0.87-1.09) | 0.6348 |
| **C18:3 LPC** | HMDB0010387* | Glycerophosphocholines | 1.03 (0.94-1.13) | 0.4686 | 1.03 (0.94-1.13) | 0.5796 | 1.03 (0.92-1.14) | 0.6398 |
| **C18:0 CE** | HMDB0010368 | Steroid esters | 1 (0.91-1.09) | 0.9910 | 0.99 (0.91-1.09) | 0.8906 | 1.02 (0.92-1.14) | 0.6526 |
| **C49:2 TAG** | HMDB0011706* | Triradylcglycerols  (<3 DB) | 0.95 (0.88-1.04) | 0.2856 | 0.97 (0.89-1.06) | 0.5470 | 0.98 (0.88-1.08) | 0.6540 |
| **C52:2 TAG** | HMDB0005369* | Triradylcglycerols  (<3 DB) | 0.91 (0.83-0.99) | 0.0384 | 0.96 (0.87-1.06) | 0.3996 | 0.97 (0.87-1.09) | 0.6551 |
| **uracil** | HMDB0000300 | Fatty acids and conjugates | 0.96 (0.87-1.06) | 0.4144 | 0.97 (0.88-1.07) | 0.5269 | 0.98 (0.87-1.09) | 0.6605 |
| **C38:6 PC** | HMDB0007991* | Glycerophosphocholines | 1.07 (0.97-1.17) | 0.1719 | 1.06 (0.97-1.17) | 0.2011 | 1.03 (0.92-1.15) | 0.6608 |
| **C24:1 SM** | HMDB0012107* | Phosphosphingolipids | 1.08 (0.98-1.18) | 0.1230 | 1.08 (0.98-1.19) | 0.1123 | 1.02 (0.92-1.14) | 0.6661 |
| **C50:4 TAG** | HMDB0005435* | Triradylcglycerols (>=3 DB) | 0.93 (0.85-1.02) | 0.1093 | 0.96 (0.88-1.06) | 0.4297 | 0.98 (0.88-1.08) | 0.6736 |
| **fucose** | HMDB0000174 | Amino acids, peptides, and analogues | 0.95 (0.87-1.04) | 0.2624 | 0.97 (0.89-1.06) | 0.5443 | 1.02 (0.92-1.13) | 0.6744 |
| **C36:5 PC plasmalogen-A** | HMDB0011221* | Glycerophosphocholines | 1.01 (0.93-1.1) | 0.8196 | 1.01 (0.92-1.1) | 0.8420 | 0.98 (0.89-1.08) | 0.6815 |
| **C36:3 PE plasmalogen** | HMDB0011441* | Glycerophosphocholines | 1.02 (0.93-1.11) | 0.6600 | 1 (0.92-1.09) | 0.9830 | 1.02 (0.92-1.13) | 0.6820 |
| **C36:2 DAG** | HMDB0007218* | Diacylglycerols | 0.94 (0.86-1.03) | 0.1819 | 0.99 (0.9-1.08) | 0.7668 | 1.02 (0.92-1.14) | 0.6855 |
| **adipate/methylglutarate** | HMDB0000448* | Fatty acids and conjugates | 1.04 (0.95-1.14) | 0.4210 | 1.03 (0.94-1.13) | 0.5584 | 1.02 (0.92-1.13) | 0.6966 |
| **C34:2 PC** | HMDB0007973* | Glycerophosphocholines | 0.99 (0.9-1.08) | 0.8126 | 1 (0.92-1.1) | 0.9220 | 0.98 (0.88-1.09) | 0.6967 |
| **C56:9 TAG** | HMDB0005448* | Triradylcglycerols (>=3 DB) | 1.01 (0.93-1.11) | 0.7575 | 1.02 (0.93-1.11) | 0.6891 | 0.98 (0.88-1.09) | 0.7067 |
| **C20:4 LPC** | HMDB0010395 | Glycerophosphocholines | 1.04 (0.95-1.14) | 0.3692 | 1.03 (0.94-1.12) | 0.5687 | 1.02 (0.92-1.13) | 0.7082 |
| **C16:0 Ceramide (d18:1)** | HMDB0004949 | Ceramides | 0.99 (0.9-1.08) | 0.7705 | 1.01 (0.92-1.11) | 0.7823 | 1.02 (0.92-1.13) | 0.7169 |
| **C14:0 LPC** | HMDB0010379 | Glycerophosphocholines | 0.98 (0.9-1.07) | 0.6234 | 0.99 (0.91-1.09) | 0.9014 | 0.98 (0.89-1.08) | 0.7177 |
| **C38:4 PC** | HMDB0008048* | Glycerophosphocholines | 0.96 (0.88-1.06) | 0.4295 | 0.99 (0.9-1.09) | 0.8472 | 0.98 (0.88-1.09) | 0.7245 |
| **suberate** | HMDB0000893 | Fatty acids and conjugates | 1 (0.9-1.11) | 0.9652 | 1.01 (0.9-1.12) | 0.9232 | 1.02 (0.9-1.16) | 0.7248 |
| **pentose monophosphate** | HMDB0001548 | . | 1.08 (0.98-1.18) | 0.1260 | 1.04 (0.94-1.15) | 0.4124 | 1.02 (0.92-1.13) | 0.7330 |
| **C24:0 Ceramide (d18:1)** | HMDB0004956 | Ceramides | 0.97 (0.89-1.06) | 0.5022 | 1 (0.91-1.09) | 0.9439 | 0.98 (0.89-1.09) | 0.7477 |
| **C52:5 TAG** | HMDB0005380* | Triradylcglycerols (>=3 DB) | 0.98 (0.9-1.06) | 0.5961 | 0.99 (0.91-1.08) | 0.7843 | 1.02 (0.92-1.12) | 0.7486 |
| **C56:2 TAG** | HMDB0005404* | Triradylcglycerols  (<3 DB) | 0.97 (0.89-1.07) | 0.5620 | 1 (0.91-1.1) | 0.9330 | 1.02 (0.91-1.13) | 0.7560 |
| **C38:5 PE** | HMDB0009069* | Glycerophosphocholines | 0.99 (0.9-1.08) | 0.7944 | 1 (0.92-1.1) | 0.9348 | 1.02 (0.92-1.13) | 0.7768 |
| **C16:0 CE** | HMDB0000885 | Steroid esters | 1.05 (0.95-1.15) | 0.3438 | 1 (0.91-1.11) | 0.9562 | 0.98 (0.88-1.1) | 0.7770 |
| **C34:2 DAG** | HMDB0007103* | Lineolic acids and derivatives | 0.94 (0.86-1.03) | 0.2060 | 0.99 (0.9-1.09) | 0.7911 | 1.02 (0.91-1.13) | 0.7820 |
| **C40:9 PC** | HMDB0008731* | Glycerophosphocholines | 1.06 (0.96-1.16) | 0.2415 | 1.05 (0.96-1.15) | 0.3029 | 1.02 (0.91-1.13) | 0.7892 |
| **C38:6 PE** | HMDB0009102* | Glycerophosphocholines | 1.02 (0.93-1.12) | 0.6516 | 1.04 (0.95-1.15) | 0.4011 | 1.01 (0.91-1.13) | 0.7916 |
| **glutamate** | HMDB0000148 | Amino acids, peptides, and analogues | 0.98 (0.89-1.08) | 0.6685 | 1.02 (0.92-1.13) | 0.7504 | 1.02 (0.9-1.14) | 0.7996 |
| **C34:0 DAG** | HMDB0007100* | Diacylglycerols | 0.94 (0.86-1.03) | 0.2090 | 0.98 (0.89-1.08) | 0.6624 | 0.99 (0.89-1.1) | 0.8012 |
| **C36:2 PC** | HMDB0008039* | Glycerophosphocholines | 1.01 (0.93-1.11) | 0.7817 | 1.02 (0.93-1.12) | 0.6257 | 1.01 (0.92-1.12) | 0.8044 |
| **C38:4 PE** | HMDB0009003* | Glycerophosphocholines | 0.98 (0.9-1.07) | 0.6863 | 1.02 (0.92-1.12) | 0.7443 | 1.01 (0.91-1.13) | 0.8142 |
| **C24:1 Ceramide (d18:1)** | HMDB0004953* | Ceramides | 0.97 (0.89-1.06) | 0.4795 | 0.99 (0.91-1.08) | 0.8373 | 0.99 (0.89-1.09) | 0.8196 |
| **erythronate/threonate** | HMDB0000613* | Carbohydrates and carbohydrate conjugates | 1.02 (0.93-1.11) | 0.7168 | 1 (0.91-1.1) | 0.9455 | 1.01 (0.91-1.12) | 0.8319 |
| **C16:0 LPC** | HMDB0010382 | Glycerophosphocholines | 1.02 (0.93-1.12) | 0.6418 | 1.01 (0.93-1.11) | 0.7575 | 1.01 (0.91-1.12) | 0.8373 |
| **adonitol/arabitol** | HMDB0000508* | Carbohydrates and carbohydrate conjugates | 1.03 (0.94-1.13) | 0.5137 | 1.03 (0.94-1.13) | 0.4932 | 1.01 (0.91-1.12) | 0.8492 |
| **chenodeoxycholate/deoxycholate** | HMDB0000626 | alcohols and derivatives | 0.97 (0.89-1.06) | 0.5307 | 0.98 (0.9-1.07) | 0.7114 | 0.99 (0.9-1.09) | 0.8495 |
| **C22:1 SM** | HMDB0012104* | Phosphosphingolipids | 0.99 (0.9-1.09) | 0.8593 | 1.01 (0.92-1.11) | 0.8177 | 1.01 (0.91-1.12) | 0.8521 |
| **C54:6 TAG** | HMDB0005391* | Triradylcglycerols (>=3 DB) | 1.01 (0.93-1.1) | 0.8583 | 0.99 (0.91-1.08) | 0.8246 | 1.01 (0.91-1.11) | 0.8541 |
| **C20:4 CE** | HMDB0006726 | Steroid esters | 1.04 (0.96-1.14) | 0.3522 | 1.01 (0.93-1.11) | 0.7574 | 1.01 (0.91-1.12) | 0.8683 |
| **C54:2 TAG** | HMDB0005403* | Triradylcglycerols  (<3 DB) | 0.93 (0.85-1.02) | 0.1389 | 0.97 (0.88-1.07) | 0.5809 | 0.99 (0.89-1.11) | 0.8778 |
| **C50:3 TAG** | HMDB0005433* | Triradylcglycerols (>=3 DB) | 0.93 (0.85-1.01) | 0.0985 | 0.97 (0.88-1.07) | 0.5329 | 0.99 (0.89-1.1) | 0.8816 |
| **C36:4 PE** | HMDB0008937* | Glycerophosphocholines | 0.99 (0.9-1.08) | 0.7717 | 1.01 (0.92-1.11) | 0.7968 | 0.99 (0.89-1.1) | 0.8983 |
| **C55:2 TAG** | HMDB0042226* | Triradylcglycerols  (<3 DB) | 0.95 (0.87-1.04) | 0.3002 | 0.98 (0.9-1.08) | 0.7411 | 0.99 (0.89-1.11) | 0.9154 |
| **C53:2 TAG** | HMDB0042196* | Triradylcglycerols  (<3 DB) | 0.93 (0.85-1.02) | 0.1061 | 0.96 (0.88-1.05) | 0.3691 | 0.99 (0.9-1.1) | 0.9243 |
| **C38:5 DAG** | HMDB0007199* | Diacylglycerols | 0.96 (0.88-1.05) | 0.3909 | 0.99 (0.9-1.09) | 0.8579 | 1 (0.91-1.12) | 0.9271 |
| **C36:2 PE** | HMDB0008994* | Glycerophosphocholines | 0.98 (0.9-1.07) | 0.6888 | 1.01 (0.92-1.11) | 0.8496 | 1 (0.91-1.11) | 0.9307 |
| **C20:0 SM** | HMDB0012102 | Phosphosphingolipids | 1 (0.91-1.1) | 0.9681 | 1.02 (0.93-1.12) | 0.6502 | 1 (0.9-1.12) | 0.9354 |
| **3-methyladipate/pimelate** | HMDB0000555* | Fatty acids and conjugates | 0.99 (0.89-1.1) | 0.8240 | 1.02 (0.92-1.15) | 0.6665 | 1 (0.89-1.14) | 0.9474 |
| **thymine** | HMDB0000262 | Pyrimidines and pyrimidine derivatives | 1.02 (0.93-1.12) | 0.7173 | 1.01 (0.91-1.11) | 0.9071 | 1 (0.9-1.12) | 0.9520 |
| **C34:1 DAG** | HMDB0007102* | Diacylglycerols | 0.93 (0.85-1.02) | 0.1066 | 0.97 (0.88-1.07) | 0.5654 | 1 (0.89-1.11) | 0.9566 |
| **C56:1 TAG** | HMDB0005396* | Triradylcglycerols  (<3 DB) | 0.96 (0.88-1.05) | 0.3965 | 0.99 (0.9-1.08) | 0.7872 | 1 (0.9-1.11) | 0.9623 |
| **C36:1 PE** | HMDB0008993* | Glycerophosphocholines | 0.98 (0.89-1.07) | 0.6565 | 1 (0.91-1.1) | 0.9249 | 1 (0.9-1.11) | 0.9660 |
| **C38:7 PC plasmalogen** | HMDB0011229* | Glycerophosphocholines | 1.02 (0.93-1.12) | 0.6951 | 1 (0.91-1.1) | 0.9964 | 1 (0.9-1.11) | 0.9753 |
| **C40:6 PC** | HMDB0008057* | Glycerophosphocholines | 1.03 (0.94-1.13) | 0.5387 | 1.03 (0.94-1.13) | 0.4823 | 1 (0.9-1.11) | 0.9756 |
| **C34:2 PE** | HMDB0008928* | Glycerophosphocholines | 0.98 (0.9-1.08) | 0.7318 | 1.01 (0.92-1.11) | 0.8070 | 1 (0.9-1.11) | 0.9770 |
| **C32:1 DAG** | HMDB0007099* | Diacylglycerols | 0.94 (0.86-1.03) | 0.1735 | 0.98 (0.89-1.08) | 0.7055 | 1 (0.9-1.11) | 0.9834 |
| **C49:3 TAG** | HMDB0042103* | Triradylcglycerols (>=3 DB) | 0.96 (0.88-1.04) | 0.3196 | 0.98 (0.9-1.07) | 0.6611 | 1 (0.9-1.11) | 0.9859 |
| **C18:2 CE** | HMDB0000610* | Steroid esters | 1.06 (0.96-1.16) | 0.2381 | 1.01 (0.92-1.12) | 0.8134 | 1 (0.9-1.12) | 0.9979 |

Model adjusted for BMI at age 18, weight change (from age 18 to time of first blood draw), age at menarche, parity and age at first birth, breastfeeding history, family history of breast cancer in a first degree relative, personal history of benign breast disease, physical activity, alcohol intake (by quintile), and oral contraceptive use at blood collection.
