# Supplementary material for: Prospective study of circulating metabolomic profiles and breast cancer incidence among predominantly premenopausal women
Source: Br J Cancer. 2025 Sep 4;133(9):1326–34. doi: 10.1038/s41416-025-03159-2 (PMC12572396; doi:10.1038/s41416-025-03159-2)
Supplement: Supplementary file 2 [file 41416_2025_3159_MOESM2_ESM.docx]

**Supplemental Table 2.** Odds ratios and 95% confidence intervals for associations between individual metabolites (per 1 SD increase in metabolite level) and breast cancer incidence, stratified by subgroups, Nurses’ Health Study II (1996-2011).

| METABOLITE | Premenopausal at blood collection |  | Premenopausal at dx |  | Normal BMI at blood draw |  | High BMI at blood draw |  | ER-positive |  | ER-negative |  |
| --- | --- | --- | --- | --- | --- | --- | --- | --- | --- | --- | --- | --- |
|  | OR.CI | p.value | OR.CI | p.value | OR.CI | p.value | OR.CI | p.value | OR.CI | p.value | OR.CI | p.value |
| 2-aminoadipate | 0.93 (0.84-1.04) | 0.23 | 0.93 (0.8-1.09) | 0.38 | 0.89 (0.78-1.01) | 0.08 | 1 (0.85-1.17) | 0.97 | 0.91 (0.81-1.02) | 0.11 | 1.28 (1.04-1.58) | 0.02 |
| 2-aminobutyrate | 1.08 (0.97-1.21) | 0.18 | 1.21 (1.04-1.42) | 0.01 | 1.01 (0.89-1.16) | 0.84 | 1.11 (0.95-1.3) | 0.19 | 1.1 (0.98-1.24) | 0.11 | 1.25 (1.02-1.55) | 0.04 |
| 2-aminoheptanoate | 1.01 (0.91-1.13) | 0.82 | 1.03 (0.88-1.2) | 0.72 | 1.07 (0.94-1.22) | 0.28 | 0.95 (0.82-1.11) | 0.55 | 1.07 (0.95-1.2) | 0.25 | 0.8 (0.65-0.99) | 0.04 |
| 2-hydroxy-3-methylbutyrate | 1 (0.89-1.12) | 0.97 | 1.02 (0.87-1.19) | 0.85 | 0.96 (0.84-1.1) | 0.57 | 0.92 (0.78-1.08) | 0.30 | 1 (0.88-1.12) | 0.94 | 1.23 (1-1.51) | 0.05 |
| 2-hydroxy-3-methylpentanoate/hydroxyisocaproate | 1.11 (0.99-1.24) | 0.08 | 1.09 (0.93-1.28) | 0.28 | 1.01 (0.89-1.16) | 0.87 | 1.05 (0.9-1.23) | 0.51 | 1.13 (1-1.27) | 0.04 | 0.81 (0.66-1) | 0.05 |
| 2-hydroxyglutarate | 1.08 (0.97-1.21) | 0.17 | 1.07 (0.92-1.24) | 0.39 | 1.06 (0.93-1.21) | 0.40 | 1.09 (0.93-1.27) | 0.30 | 1.07 (0.96-1.21) | 0.23 | 1.23 (1-1.52) | 0.06 |
| 3-hydroxymethylglutarate/anhydroDglucose | 1.02 (0.91-1.14) | 0.71 | 1 (0.86-1.17) | 0.97 | 0.93 (0.82-1.06) | 0.30 | 1.18 (1.01-1.39) | 0.04 | 1.08 (0.96-1.22) | 0.18 | 0.82 (0.66-1.02) | 0.07 |
| 3-methyladipate/pimelate | 1 (0.9-1.11) | 0.97 | 0.92 (0.8-1.06) | 0.26 | 0.97 (0.85-1.1) | 0.63 | 1.04 (0.89-1.2) | 0.64 | 1.01 (0.9-1.13) | 0.87 | 0.83 (0.67-1.04) | 0.10 |
| 4-pyridoxate | 0.95 (0.85-1.06) | 0.34 | 0.92 (0.79-1.07) | 0.26 | 0.97 (0.85-1.11) | 0.70 | 0.92 (0.78-1.07) | 0.27 | 0.98 (0.87-1.11) | 0.78 | 0.84 (0.69-1.03) | 0.10 |
| adipate/methylglutarate | 1.04 (0.93-1.16) | 0.49 | 1.03 (0.88-1.19) | 0.73 | 0.95 (0.84-1.07) | 0.39 | 1.13 (0.97-1.32) | 0.12 | 1.06 (0.95-1.18) | 0.32 | 0.84 (0.68-1.03) | 0.10 |
| adonitol/arabitol | 1.04 (0.93-1.17) | 0.46 | 0.99 (0.85-1.16) | 0.95 | 1.05 (0.91-1.2) | 0.50 | 0.95 (0.81-1.12) | 0.57 | 1.01 (0.89-1.14) | 0.88 | 0.85 (0.69-1.04) | 0.11 |
| alpha-hydroxybutyrate/beta-hydroxybutyrate/hydroxyisobutyrate | 1.07 (0.95-1.2) | 0.29 | 1.11 (0.95-1.3) | 0.20 | 1 (0.87-1.14) | 0.96 | 1.1 (0.94-1.29) | 0.24 | 1.01 (0.9-1.14) | 0.84 | 0.84 (0.68-1.04) | 0.11 |
| alpha-keto-beta-methylvalerate/alpha-ketoisocaproate | 1.12 (1-1.26) | 0.05 | 1.11 (0.95-1.29) | 0.20 | 1.05 (0.92-1.2) | 0.43 | 1.1 (0.94-1.29) | 0.22 | 1.1 (0.98-1.23) | 0.12 | 0.85 (0.69-1.04) | 0.12 |
| alpha-ketoisovalerate | 1.09 (0.98-1.22) | 0.12 | 1.1 (0.95-1.28) | 0.22 | 1.04 (0.91-1.19) | 0.57 | 1.12 (0.96-1.3) | 0.14 | 1.06 (0.94-1.19) | 0.33 | 0.85 (0.68-1.05) | 0.13 |
| aspartate | 1.02 (0.92-1.14) | 0.70 | 1.04 (0.9-1.21) | 0.59 | 0.94 (0.82-1.06) | 0.31 | 1.15 (0.98-1.35) | 0.08 | 0.98 (0.87-1.1) | 0.70 | 0.85 (0.69-1.05) | 0.13 |
| C14:0 CE | 0.97 (0.87-1.08) | 0.62 | 1 (0.87-1.16) | 0.96 | 0.87 (0.77-0.99) | 0.04 | 0.99 (0.85-1.16) | 0.92 | 0.93 (0.83-1.05) | 0.23 | 0.86 (0.7-1.05) | 0.15 |
| C14:0 LPC | 0.97 (0.87-1.08) | 0.57 | 0.99 (0.86-1.15) | 0.93 | 0.98 (0.86-1.11) | 0.73 | 0.91 (0.78-1.06) | 0.23 | 0.9 (0.8-1.01) | 0.07 | 0.86 (0.69-1.06) | 0.15 |
| C14:0 SM | 1.01 (0.9-1.13) | 0.88 | 1.09 (0.94-1.28) | 0.26 | 0.96 (0.84-1.1) | 0.56 | 0.99 (0.84-1.16) | 0.88 | 0.95 (0.84-1.07) | 0.38 | 0.86 (0.69-1.06) | 0.16 |
| C16:0 CE | 1.06 (0.95-1.19) | 0.31 | 1.06 (0.91-1.24) | 0.46 | 0.99 (0.87-1.14) | 0.93 | 1.05 (0.9-1.23) | 0.52 | 1.06 (0.94-1.19) | 0.34 | 0.87 (0.71-1.06) | 0.16 |
| C16:0 Ceramide (d18:1) | 1.03 (0.92-1.15) | 0.56 | 1.04 (0.89-1.22) | 0.59 | 1 (0.87-1.14) | 0.96 | 0.98 (0.85-1.14) | 0.84 | 0.93 (0.83-1.04) | 0.22 | 1.16 (0.94-1.42) | 0.17 |
| C16:0 LPC | 0.96 (0.86-1.08) | 0.51 | 0.91 (0.78-1.06) | 0.25 | 0.97 (0.85-1.11) | 0.69 | 0.96 (0.83-1.11) | 0.58 | 0.97 (0.86-1.08) | 0.54 | 1.16 (0.94-1.44) | 0.17 |
| C16:0 LPE | 0.97 (0.87-1.1) | 0.66 | 0.9 (0.76-1.06) | 0.21 | 0.92 (0.8-1.06) | 0.25 | 1.02 (0.89-1.18) | 0.75 | 0.96 (0.86-1.09) | 0.56 | 0.87 (0.71-1.06) | 0.17 |
| C16:0 SM | 1.06 (0.95-1.18) | 0.30 | 1.1 (0.94-1.28) | 0.22 | 0.99 (0.87-1.12) | 0.90 | 1.09 (0.94-1.27) | 0.25 | 1.03 (0.92-1.15) | 0.61 | 0.87 (0.71-1.07) | 0.18 |
| C16:1 CE | 0.91 (0.81-1.02) | 0.11 | 0.85 (0.72-0.99) | 0.04 | 0.83 (0.72-0.95) | 0.01 | 0.95 (0.82-1.11) | 0.52 | 0.89 (0.79-1.01) | 0.06 | 0.86 (0.69-1.07) | 0.19 |
| C16:1 LPC | 0.94 (0.84-1.05) | 0.25 | 0.88 (0.76-1.03) | 0.11 | 0.92 (0.82-1.05) | 0.22 | 0.96 (0.83-1.11) | 0.60 | 0.94 (0.84-1.05) | 0.26 | 0.87 (0.71-1.08) | 0.21 |
| C18:0 CE | 1.03 (0.93-1.16) | 0.56 | 1.02 (0.87-1.2) | 0.77 | 1.01 (0.88-1.16) | 0.90 | 1.03 (0.89-1.2) | 0.70 | 1.08 (0.96-1.21) | 0.22 | 0.87 (0.7-1.09) | 0.22 |
| C18:0 LPC | 1.04 (0.93-1.16) | 0.51 | 1.04 (0.88-1.21) | 0.66 | 1.06 (0.93-1.21) | 0.40 | 1 (0.86-1.15) | 0.96 | 1.02 (0.91-1.15) | 0.74 | 0.88 (0.72-1.08) | 0.22 |
| C18:0 LPE | 1.02 (0.91-1.14) | 0.79 | 0.97 (0.83-1.13) | 0.71 | 0.99 (0.87-1.13) | 0.90 | 1.01 (0.88-1.17) | 0.87 | 0.98 (0.87-1.1) | 0.71 | 0.88 (0.71-1.09) | 0.23 |
| C18:0 SM | 1.05 (0.94-1.17) | 0.42 | 1.08 (0.93-1.26) | 0.32 | 0.94 (0.82-1.08) | 0.40 | 1.12 (0.96-1.3) | 0.15 | 0.99 (0.88-1.12) | 0.91 | 1.13 (0.92-1.4) | 0.23 |
| C18:1 CE | 1.03 (0.92-1.16) | 0.59 | 1.04 (0.88-1.22) | 0.65 | 0.96 (0.83-1.1) | 0.55 | 1.07 (0.92-1.25) | 0.39 | 1.07 (0.95-1.21) | 0.25 | 0.88 (0.71-1.09) | 0.24 |
| C18:1 LPC | 1.01 (0.9-1.14) | 0.81 | 0.97 (0.83-1.15) | 0.74 | 0.97 (0.85-1.11) | 0.66 | 1.05 (0.89-1.22) | 0.57 | 1.01 (0.9-1.14) | 0.84 | 0.88 (0.71-1.09) | 0.24 |
| C18:1 LPE | 1.01 (0.9-1.13) | 0.83 | 0.96 (0.82-1.13) | 0.65 | 0.96 (0.85-1.1) | 0.58 | 1.06 (0.9-1.24) | 0.49 | 0.98 (0.87-1.11) | 0.78 | 0.88 (0.71-1.09) | 0.24 |
| C18:1 SM | 1.04 (0.93-1.16) | 0.50 | 1.06 (0.91-1.24) | 0.47 | 0.94 (0.82-1.07) | 0.34 | 1.14 (0.98-1.33) | 0.09 | 1 (0.89-1.13) | 0.94 | 1.13 (0.92-1.4) | 0.24 |
| C18:2 CE | 1.06 (0.94-1.19) | 0.33 | 1.09 (0.93-1.28) | 0.28 | 0.99 (0.86-1.13) | 0.83 | 1.08 (0.92-1.27) | 0.33 | 1.08 (0.96-1.22) | 0.22 | 0.88 (0.72-1.09) | 0.24 |
| C18:2 LPC | 1.02 (0.91-1.16) | 0.70 | 1.03 (0.87-1.22) | 0.72 | 1.01 (0.88-1.15) | 0.92 | 1.01 (0.85-1.19) | 0.95 | 1 (0.88-1.13) | 0.98 | 0.88 (0.72-1.09) | 0.25 |
| C18:2 LPE | 0.98 (0.88-1.1) | 0.78 | 0.96 (0.83-1.12) | 0.64 | 0.97 (0.85-1.11) | 0.67 | 1 (0.85-1.18) | 0.96 | 0.96 (0.85-1.08) | 0.48 | 0.88 (0.7-1.1) | 0.25 |
| C18:3 CE | 0.96 (0.86-1.08) | 0.51 | 0.95 (0.82-1.11) | 0.53 | 0.91 (0.8-1.04) | 0.16 | 0.94 (0.81-1.1) | 0.45 | 0.97 (0.86-1.09) | 0.61 | 0.89 (0.72-1.09) | 0.26 |
| C18:3 LPC | 0.98 (0.88-1.1) | 0.78 | 0.92 (0.79-1.07) | 0.30 | 0.98 (0.86-1.12) | 0.77 | 0.98 (0.85-1.13) | 0.79 | 0.98 (0.88-1.1) | 0.77 | 0.89 (0.72-1.09) | 0.26 |
| C20:0 SM | 1.03 (0.92-1.16) | 0.58 | 1.19 (1.02-1.4) | 0.03 | 0.99 (0.86-1.13) | 0.84 | 1.03 (0.88-1.2) | 0.75 | 0.99 (0.88-1.12) | 0.89 | 1.13 (0.91-1.41) | 0.27 |
| C20:3 CE | 1.03 (0.93-1.15) | 0.54 | 1.02 (0.88-1.19) | 0.75 | 0.97 (0.85-1.11) | 0.70 | 1.02 (0.89-1.18) | 0.78 | 1.01 (0.9-1.13) | 0.93 | 0.89 (0.72-1.1) | 0.27 |
| C20:4 CE | 1.12 (1-1.25) | 0.05 | 1.12 (0.96-1.31) | 0.15 | 1.03 (0.9-1.17) | 0.70 | 1.09 (0.94-1.26) | 0.27 | 1.1 (0.98-1.24) | 0.09 | 0.9 (0.73-1.09) | 0.28 |
| C20:4 LPC | 1.05 (0.94-1.18) | 0.37 | 1.01 (0.87-1.18) | 0.86 | 1.04 (0.92-1.19) | 0.51 | 0.96 (0.83-1.12) | 0.63 | 1.01 (0.9-1.13) | 0.93 | 0.89 (0.73-1.1) | 0.29 |
| C20:4 LPE | 1.05 (0.94-1.17) | 0.38 | 0.98 (0.84-1.14) | 0.76 | 1.04 (0.92-1.18) | 0.54 | 0.99 (0.85-1.16) | 0.91 | 1 (0.89-1.12) | 0.95 | 0.89 (0.72-1.1) | 0.29 |
| C20:5 CE | 1.03 (0.92-1.15) | 0.61 | 1.09 (0.93-1.27) | 0.30 | 0.95 (0.83-1.08) | 0.41 | 0.96 (0.83-1.12) | 0.61 | 1 (0.88-1.12) | 0.94 | 0.89 (0.73-1.1) | 0.29 |
| C20:5 LPC | 1.01 (0.9-1.15) | 0.82 | 1.02 (0.86-1.2) | 0.84 | 1 (0.87-1.15) | 1.00 | 0.99 (0.84-1.18) | 0.94 | 0.99 (0.87-1.12) | 0.89 | 1.12 (0.91-1.38) | 0.30 |
| C22:0 Ceramide (d18:1) | 0.96 (0.86-1.07) | 0.44 | 1.04 (0.89-1.22) | 0.61 | 0.98 (0.85-1.12) | 0.76 | 0.91 (0.78-1.06) | 0.23 | 0.92 (0.82-1.04) | 0.20 | 0.9 (0.73-1.11) | 0.31 |
| C22:0 LPE | 1.04 (0.92-1.17) | 0.57 | 1.02 (0.86-1.2) | 0.86 | 1.04 (0.9-1.2) | 0.59 | 1.02 (0.88-1.2) | 0.76 | 1.03 (0.91-1.16) | 0.65 | 0.9 (0.74-1.1) | 0.32 |
| C22:1 SM | 1.01 (0.91-1.13) | 0.80 | 1.14 (0.98-1.34) | 0.09 | 0.98 (0.86-1.11) | 0.73 | 1.02 (0.88-1.2) | 0.76 | 0.99 (0.88-1.11) | 0.85 | 0.89 (0.71-1.12) | 0.32 |
| C22:5 CE | 1.02 (0.91-1.15) | 0.68 | 1.07 (0.91-1.26) | 0.41 | 0.95 (0.83-1.09) | 0.46 | 1.03 (0.87-1.21) | 0.75 | 1.01 (0.89-1.14) | 0.88 | 1.11 (0.9-1.37) | 0.33 |
| C22:6 CE | 1.13 (1-1.26) | 0.04 | 1.19 (1.02-1.4) | 0.03 | 1.06 (0.92-1.22) | 0.44 | 1.13 (0.97-1.32) | 0.12 | 1.13 (1-1.28) | 0.05 | 0.9 (0.73-1.11) | 0.33 |
| C22:6 LPC | 1.02 (0.91-1.15) | 0.69 | 1.05 (0.9-1.22) | 0.53 | 1.04 (0.91-1.18) | 0.61 | 0.99 (0.85-1.16) | 0.94 | 0.99 (0.88-1.11) | 0.82 | 0.9 (0.73-1.12) | 0.35 |
| C22:6 LPE | 1.01 (0.9-1.13) | 0.91 | 1.03 (0.88-1.21) | 0.69 | 1.03 (0.9-1.18) | 0.63 | 0.99 (0.84-1.16) | 0.88 | 0.98 (0.87-1.1) | 0.74 | 0.91 (0.73-1.12) | 0.36 |
| C24:0 Ceramide (d18:1) | 0.99 (0.88-1.1) | 0.82 | 1.09 (0.93-1.27) | 0.27 | 1 (0.87-1.14) | 0.95 | 0.94 (0.81-1.09) | 0.39 | 0.95 (0.84-1.06) | 0.35 | 0.9 (0.72-1.13) | 0.36 |
| C24:1 Ceramide (d18:1) | 0.98 (0.88-1.09) | 0.68 | 1.03 (0.88-1.2) | 0.71 | 0.92 (0.8-1.05) | 0.21 | 1.04 (0.9-1.2) | 0.59 | 0.95 (0.85-1.06) | 0.36 | 1.11 (0.89-1.38) | 0.37 |
| C24:1 SM | 1.04 (0.93-1.16) | 0.50 | 1.09 (0.93-1.28) | 0.28 | 0.97 (0.84-1.11) | 0.63 | 1.09 (0.93-1.28) | 0.29 | 1 (0.89-1.13) | 0.99 | 0.91 (0.73-1.13) | 0.37 |
| C30:0 PC | 0.97 (0.87-1.09) | 0.61 | 1.02 (0.87-1.19) | 0.81 | 0.93 (0.81-1.06) | 0.28 | 0.97 (0.84-1.13) | 0.71 | 0.87 (0.78-0.99) | 0.03 | 0.91 (0.72-1.13) | 0.38 |
| C30:1 PC | 0.95 (0.84-1.06) | 0.35 | 0.96 (0.82-1.12) | 0.60 | 0.89 (0.78-1.03) | 0.11 | 0.97 (0.84-1.13) | 0.73 | 0.86 (0.76-0.97) | 0.02 | 1.1 (0.88-1.36) | 0.40 |
| C32:0 DAG | 0.93 (0.82-1.04) | 0.20 | 0.87 (0.73-1.02) | 0.09 | 0.95 (0.82-1.09) | 0.45 | 0.91 (0.78-1.07) | 0.26 | 0.88 (0.77-0.99) | 0.04 | 0.91 (0.74-1.13) | 0.40 |
| C32:0 PC | 0.99 (0.89-1.11) | 0.91 | 1 (0.85-1.18) | 0.98 | 0.96 (0.83-1.1) | 0.54 | 0.98 (0.85-1.14) | 0.83 | 0.94 (0.83-1.07) | 0.35 | 0.91 (0.73-1.14) | 0.41 |
| C32:0 PE | 0.99 (0.89-1.11) | 0.93 | 1.04 (0.9-1.21) | 0.59 | 0.93 (0.82-1.06) | 0.28 | 1.02 (0.88-1.18) | 0.81 | 0.91 (0.81-1.02) | 0.10 | 0.92 (0.74-1.13) | 0.41 |
| C32:1 DAG | 0.96 (0.85-1.07) | 0.44 | 0.91 (0.77-1.07) | 0.26 | 1 (0.87-1.15) | 0.99 | 0.92 (0.78-1.07) | 0.27 | 0.89 (0.79-1.01) | 0.07 | 0.92 (0.74-1.13) | 0.41 |
| C32:1 PC | 0.89 (0.79-1.01) | 0.07 | 0.92 (0.77-1.08) | 0.30 | 0.85 (0.73-0.98) | 0.03 | 0.94 (0.8-1.11) | 0.48 | 0.85 (0.75-0.96) | 0.01 | 0.92 (0.75-1.13) | 0.42 |
| C32:2 PC | 0.96 (0.86-1.08) | 0.48 | 1.03 (0.88-1.21) | 0.68 | 1 (0.87-1.15) | 0.99 | 0.91 (0.78-1.06) | 0.22 | 0.9 (0.8-1.01) | 0.08 | 0.92 (0.75-1.13) | 0.42 |
| C34:0 DAG | 0.96 (0.86-1.08) | 0.50 | 0.85 (0.72-1) | 0.05 | 0.97 (0.84-1.11) | 0.66 | 0.93 (0.8-1.08) | 0.36 | 0.91 (0.8-1.02) | 0.11 | 0.91 (0.73-1.15) | 0.43 |
| C34:0 PE | 1 (0.89-1.11) | 0.95 | 1.06 (0.91-1.23) | 0.47 | 0.92 (0.8-1.05) | 0.20 | 1.04 (0.89-1.2) | 0.64 | 0.92 (0.82-1.03) | 0.16 | 0.91 (0.73-1.14) | 0.43 |
| C34:0 PS | 0.91 (0.81-1.02) | 0.10 | 0.95 (0.82-1.11) | 0.54 | 0.92 (0.8-1.05) | 0.20 | 0.81 (0.69-0.96) | 0.01 | 0.86 (0.76-0.97) | 0.02 | 0.92 (0.75-1.13) | 0.43 |
| C34:1 DAG | 0.94 (0.83-1.05) | 0.28 | 0.9 (0.76-1.06) | 0.20 | 0.99 (0.86-1.14) | 0.84 | 0.9 (0.77-1.05) | 0.20 | 0.89 (0.78-1) | 0.05 | 0.92 (0.74-1.14) | 0.43 |
| C34:1 PC | 0.88 (0.78-0.99) | 0.03 | 0.89 (0.76-1.05) | 0.19 | 0.82 (0.71-0.94) | 0.01 | 0.94 (0.81-1.1) | 0.46 | 0.85 (0.75-0.96) | 0.01 | 0.92 (0.74-1.14) | 0.44 |
| C34:1 PC plasmalogen | 1.1 (0.99-1.23) | 0.08 | 1.14 (0.98-1.32) | 0.10 | 1.03 (0.9-1.16) | 0.70 | 1.17 (1-1.36) | 0.05 | 1.12 (1-1.26) | 0.05 | 0.92 (0.74-1.14) | 0.44 |
| C34:1 PC plasmalogen-B | 1.09 (0.98-1.21) | 0.13 | 1.13 (0.97-1.32) | 0.11 | 1.03 (0.9-1.16) | 0.70 | 1.12 (0.96-1.31) | 0.16 | 1.09 (0.97-1.22) | 0.17 | 1.09 (0.88-1.35) | 0.45 |
| C34:2 DAG | 0.93 (0.83-1.04) | 0.22 | 0.9 (0.76-1.06) | 0.21 | 1.01 (0.88-1.15) | 0.94 | 0.91 (0.78-1.07) | 0.26 | 0.92 (0.81-1.03) | 0.15 | 0.92 (0.74-1.15) | 0.45 |
| C34:2 PC | 0.96 (0.86-1.08) | 0.51 | 1.03 (0.89-1.21) | 0.68 | 0.98 (0.86-1.12) | 0.77 | 0.93 (0.8-1.09) | 0.37 | 0.92 (0.82-1.03) | 0.14 | 0.92 (0.74-1.14) | 0.46 |
| C34:2 PC plasmalogen | 1.07 (0.96-1.21) | 0.23 | 1.09 (0.93-1.28) | 0.27 | 0.97 (0.85-1.12) | 0.71 | 1.14 (0.98-1.34) | 0.10 | 1.07 (0.95-1.21) | 0.27 | 0.93 (0.76-1.14) | 0.47 |
| C34:2 PE | 0.96 (0.86-1.08) | 0.52 | 0.97 (0.82-1.13) | 0.67 | 0.97 (0.84-1.12) | 0.66 | 0.98 (0.85-1.14) | 0.80 | 0.92 (0.81-1.03) | 0.15 | 0.93 (0.75-1.14) | 0.47 |
| C34:2 PE plasmalogen | 1.05 (0.94-1.17) | 0.40 | 1.06 (0.91-1.22) | 0.46 | 1 (0.88-1.14) | 0.99 | 1.06 (0.92-1.22) | 0.44 | 1.04 (0.93-1.17) | 0.46 | 0.93 (0.76-1.14) | 0.49 |
| C34:3 DAG | 0.95 (0.85-1.07) | 0.42 | 0.94 (0.8-1.1) | 0.45 | 1.07 (0.94-1.23) | 0.31 | 0.9 (0.77-1.06) | 0.20 | 0.96 (0.85-1.09) | 0.55 | 0.93 (0.75-1.15) | 0.50 |
| C34:3 PC | 0.87 (0.77-0.97) | 0.01 | 0.91 (0.78-1.07) | 0.25 | 0.9 (0.79-1.04) | 0.15 | 0.87 (0.75-1.01) | 0.07 | 0.87 (0.77-0.98) | 0.02 | 0.93 (0.74-1.16) | 0.51 |
| C34:3 PC plasmalogen | 1.08 (0.97-1.2) | 0.17 | 1.12 (0.97-1.3) | 0.12 | 1.04 (0.91-1.18) | 0.55 | 1.05 (0.9-1.22) | 0.57 | 1.03 (0.92-1.16) | 0.62 | 0.93 (0.76-1.15) | 0.51 |
| C34:3 PE plasmalogen | 1.04 (0.93-1.15) | 0.50 | 1.03 (0.89-1.19) | 0.69 | 1 (0.88-1.14) | 0.96 | 1.03 (0.9-1.19) | 0.65 | 1 (0.89-1.12) | 0.99 | 0.93 (0.75-1.16) | 0.51 |
| C34:4 PC | 0.95 (0.84-1.07) | 0.38 | 1.01 (0.86-1.18) | 0.94 | 1 (0.87-1.15) | 0.99 | 0.85 (0.72-0.99) | 0.04 | 0.88 (0.78-0.99) | 0.03 | 1.07 (0.87-1.32) | 0.52 |
| C34:5 PC plasmalogen | 1.07 (0.96-1.19) | 0.20 | 1.03 (0.89-1.19) | 0.71 | 1.03 (0.91-1.17) | 0.64 | 1.04 (0.9-1.21) | 0.62 | 1.01 (0.9-1.12) | 0.92 | 1.07 (0.86-1.34) | 0.52 |
| C36:0 PE | 0.98 (0.88-1.09) | 0.70 | 1.03 (0.89-1.19) | 0.71 | 0.91 (0.8-1.04) | 0.16 | 1.01 (0.87-1.17) | 0.87 | 0.9 (0.8-1.01) | 0.07 | 0.93 (0.75-1.16) | 0.52 |
| C36:1 DAG | 0.92 (0.82-1.04) | 0.18 | 0.89 (0.76-1.05) | 0.16 | 0.95 (0.82-1.09) | 0.45 | 0.89 (0.76-1.05) | 0.16 | 0.88 (0.78-0.99) | 0.04 | 1.07 (0.87-1.31) | 0.53 |
| C36:1 PC | 0.93 (0.84-1.04) | 0.23 | 0.96 (0.83-1.12) | 0.64 | 0.87 (0.76-1) | 0.04 | 0.97 (0.85-1.12) | 0.72 | 0.9 (0.8-1.01) | 0.07 | 0.93 (0.75-1.16) | 0.53 |
| C36:1 PE | 0.97 (0.87-1.09) | 0.65 | 1.01 (0.86-1.19) | 0.87 | 0.98 (0.85-1.13) | 0.77 | 0.95 (0.82-1.11) | 0.54 | 0.92 (0.81-1.04) | 0.17 | 0.93 (0.75-1.16) | 0.53 |
| C36:1 PE plasmalogen | 1.03 (0.92-1.15) | 0.61 | 1.03 (0.88-1.21) | 0.69 | 0.95 (0.83-1.09) | 0.48 | 1.13 (0.96-1.32) | 0.14 | 1.02 (0.91-1.15) | 0.72 | 0.93 (0.75-1.16) | 0.53 |
| C36:2 DAG | 0.95 (0.85-1.06) | 0.37 | 0.95 (0.8-1.11) | 0.50 | 1.02 (0.89-1.16) | 0.82 | 0.91 (0.78-1.07) | 0.26 | 0.93 (0.83-1.05) | 0.26 | 0.93 (0.75-1.16) | 0.54 |
| C36:2 PC | 1 (0.89-1.11) | 0.95 | 1.08 (0.93-1.26) | 0.31 | 1.02 (0.9-1.16) | 0.73 | 0.95 (0.82-1.1) | 0.48 | 0.95 (0.85-1.06) | 0.34 | 0.93 (0.75-1.16) | 0.54 |
| C36:2 PC plasmalogen | 1.11 (1-1.24) | 0.05 | 1.15 (0.99-1.33) | 0.08 | 1.05 (0.92-1.19) | 0.48 | 1.18 (1-1.38) | 0.05 | 1.12 (1-1.26) | 0.05 | 0.94 (0.76-1.16) | 0.54 |
| C36:2 PE | 0.95 (0.85-1.07) | 0.42 | 0.97 (0.82-1.14) | 0.70 | 1 (0.86-1.15) | 0.96 | 0.92 (0.79-1.07) | 0.30 | 0.91 (0.8-1.03) | 0.12 | 0.94 (0.76-1.16) | 0.54 |
| C36:2 PE plasmalogen | 1.06 (0.95-1.18) | 0.31 | 1.09 (0.95-1.27) | 0.23 | 1 (0.88-1.14) | 0.96 | 1.04 (0.9-1.21) | 0.57 | 1.03 (0.92-1.15) | 0.62 | 0.93 (0.75-1.16) | 0.54 |
| C36:3 DAG | 0.97 (0.87-1.08) | 0.61 | 0.99 (0.85-1.16) | 0.93 | 1.1 (0.97-1.26) | 0.14 | 0.91 (0.78-1.07) | 0.25 | 1 (0.89-1.12) | 1.00 | 0.93 (0.74-1.17) | 0.55 |
| C36:3 PC | 0.95 (0.85-1.06) | 0.34 | 0.98 (0.84-1.15) | 0.84 | 0.99 (0.86-1.13) | 0.83 | 0.94 (0.8-1.09) | 0.39 | 0.94 (0.84-1.06) | 0.32 | 0.94 (0.76-1.16) | 0.56 |
| C36:3 PC plasmalogen | 1.11 (0.99-1.24) | 0.07 | 1.14 (0.98-1.32) | 0.09 | 1.01 (0.89-1.16) | 0.83 | 1.13 (0.97-1.32) | 0.13 | 1.06 (0.94-1.19) | 0.36 | 0.94 (0.77-1.15) | 0.56 |
| C36:3 PE | 0.99 (0.89-1.11) | 0.92 | 1.01 (0.87-1.19) | 0.86 | 1.02 (0.89-1.17) | 0.78 | 0.98 (0.84-1.14) | 0.80 | 0.96 (0.85-1.08) | 0.52 | 0.93 (0.73-1.18) | 0.56 |
| C36:3 PE plasmalogen | 1.04 (0.94-1.16) | 0.44 | 1.05 (0.91-1.21) | 0.52 | 0.98 (0.86-1.11) | 0.71 | 1.05 (0.91-1.22) | 0.52 | 0.99 (0.88-1.11) | 0.88 | 0.94 (0.76-1.16) | 0.56 |
| C36:4 DAG | 0.99 (0.88-1.1) | 0.81 | 1.01 (0.87-1.18) | 0.88 | 1.17 (1.02-1.33) | 0.02 | 0.88 (0.75-1.03) | 0.12 | 1.03 (0.92-1.16) | 0.62 | 0.94 (0.76-1.16) | 0.56 |
| C36:4 PC plasmalogen | 1.07 (0.96-1.2) | 0.20 | 1.11 (0.95-1.29) | 0.19 | 0.98 (0.87-1.12) | 0.81 | 1.16 (0.99-1.36) | 0.07 | 1.1 (0.98-1.24) | 0.10 | 1.07 (0.86-1.33) | 0.57 |
| C36:4 PC-A | 0.94 (0.85-1.05) | 0.27 | 1 (0.86-1.15) | 0.95 | 0.99 (0.87-1.13) | 0.91 | 0.9 (0.77-1.04) | 0.15 | 0.93 (0.83-1.04) | 0.22 | 0.94 (0.75-1.17) | 0.57 |
| C36:4 PC-B | 1.04 (0.92-1.17) | 0.57 | 1.03 (0.87-1.22) | 0.70 | 1.04 (0.9-1.2) | 0.59 | 0.91 (0.77-1.07) | 0.26 | 0.96 (0.85-1.09) | 0.53 | 1.07 (0.86-1.33) | 0.57 |
| C36:4 PE | 1.01 (0.9-1.14) | 0.88 | 1 (0.85-1.18) | 0.97 | 1.01 (0.87-1.17) | 0.90 | 0.96 (0.83-1.12) | 0.63 | 0.92 (0.81-1.04) | 0.16 | 1.06 (0.86-1.31) | 0.57 |
| C36:4 PE plasmalogen | 1.03 (0.93-1.15) | 0.54 | 1.01 (0.87-1.16) | 0.91 | 0.97 (0.85-1.1) | 0.64 | 1.08 (0.93-1.24) | 0.32 | 1.01 (0.9-1.13) | 0.87 | 1.06 (0.86-1.32) | 0.57 |
| C36:5 PC plasmalogen-A | 0.99 (0.89-1.1) | 0.88 | 1.01 (0.87-1.17) | 0.89 | 0.99 (0.87-1.12) | 0.86 | 0.87 (0.74-1.01) | 0.07 | 0.94 (0.84-1.05) | 0.25 | 0.94 (0.75-1.17) | 0.57 |
| C36:5 PC plasmalogen-B | 1.06 (0.95-1.18) | 0.30 | 1.05 (0.92-1.21) | 0.46 | 1.06 (0.94-1.2) | 0.36 | 0.95 (0.81-1.11) | 0.49 | 0.99 (0.88-1.11) | 0.84 | 1.06 (0.87-1.29) | 0.58 |
| C36:5 PE plasmalogen | 1.07 (0.96-1.19) | 0.22 | 1.04 (0.9-1.19) | 0.63 | 1.06 (0.94-1.21) | 0.34 | 1.02 (0.87-1.19) | 0.82 | 1.01 (0.9-1.13) | 0.89 | 1.06 (0.86-1.31) | 0.58 |
| C38:2 PC | 0.93 (0.83-1.04) | 0.21 | 0.99 (0.84-1.16) | 0.87 | 0.96 (0.83-1.11) | 0.61 | 0.92 (0.79-1.06) | 0.24 | 0.89 (0.79-1.01) | 0.06 | 1.06 (0.86-1.3) | 0.58 |
| C38:2 PE | 1.01 (0.91-1.13) | 0.80 | 1.13 (0.97-1.31) | 0.12 | 0.99 (0.87-1.12) | 0.85 | 1.02 (0.88-1.19) | 0.78 | 0.95 (0.84-1.06) | 0.35 | 0.94 (0.75-1.17) | 0.58 |
| C38:3 PC | 0.94 (0.83-1.06) | 0.29 | 0.97 (0.82-1.14) | 0.68 | 1.01 (0.87-1.17) | 0.88 | 0.87 (0.74-1.02) | 0.09 | 0.88 (0.78-1) | 0.05 | 0.94 (0.76-1.17) | 0.58 |
| C38:3 PE plasmalogen | 1.06 (0.95-1.18) | 0.29 | 1.08 (0.93-1.25) | 0.30 | 1 (0.88-1.14) | 0.97 | 1.04 (0.89-1.2) | 0.63 | 0.99 (0.88-1.11) | 0.86 | 0.94 (0.75-1.18) | 0.58 |
| C38:4 PC | 1.05 (0.94-1.18) | 0.38 | 1.08 (0.92-1.28) | 0.33 | 1.08 (0.94-1.23) | 0.29 | 0.92 (0.78-1.07) | 0.27 | 0.99 (0.87-1.11) | 0.82 | 1.06 (0.87-1.29) | 0.58 |
| C38:4 PC plasmalogen | 1.09 (0.98-1.21) | 0.13 | 1.1 (0.95-1.28) | 0.22 | 1.04 (0.92-1.17) | 0.57 | 1.07 (0.91-1.26) | 0.42 | 1.07 (0.95-1.2) | 0.26 | 0.94 (0.77-1.16) | 0.58 |
| C38:4 PE | 1.02 (0.91-1.14) | 0.76 | 1.03 (0.88-1.22) | 0.69 | 1.05 (0.91-1.22) | 0.48 | 0.94 (0.8-1.09) | 0.39 | 0.94 (0.83-1.07) | 0.35 | 0.94 (0.76-1.17) | 0.60 |
| C38:5 DAG | 0.98 (0.88-1.1) | 0.78 | 0.95 (0.82-1.12) | 0.55 | 1.07 (0.93-1.22) | 0.34 | 0.87 (0.75-1.02) | 0.09 | 0.97 (0.86-1.09) | 0.57 | 1.05 (0.86-1.29) | 0.61 |
| C38:5 PE | 1.01 (0.9-1.13) | 0.88 | 1.02 (0.87-1.19) | 0.82 | 1.04 (0.91-1.19) | 0.57 | 0.95 (0.82-1.11) | 0.54 | 0.96 (0.85-1.09) | 0.56 | 0.95 (0.77-1.16) | 0.61 |
| C38:5 PE plasmalogen | 1.09 (0.98-1.22) | 0.11 | 1.06 (0.92-1.22) | 0.45 | 1.06 (0.93-1.2) | 0.40 | 1.03 (0.88-1.2) | 0.73 | 1.01 (0.9-1.13) | 0.93 | 0.95 (0.77-1.17) | 0.61 |
| C38:6 PC | 1.02 (0.91-1.15) | 0.71 | 1.1 (0.94-1.29) | 0.24 | 1.05 (0.91-1.2) | 0.50 | 0.95 (0.81-1.13) | 0.59 | 0.99 (0.88-1.12) | 0.90 | 0.94 (0.76-1.18) | 0.62 |
| C38:6 PE | 1 (0.88-1.12) | 0.95 | 1.07 (0.91-1.26) | 0.41 | 1.04 (0.9-1.2) | 0.58 | 0.95 (0.82-1.12) | 0.56 | 0.95 (0.84-1.08) | 0.41 | 0.95 (0.77-1.17) | 0.62 |
| C38:6 PE plasmalogen | 1.05 (0.95-1.17) | 0.33 | 1.02 (0.89-1.18) | 0.78 | 1.01 (0.89-1.15) | 0.87 | 1.02 (0.88-1.19) | 0.78 | 0.99 (0.89-1.11) | 0.91 | 0.95 (0.76-1.18) | 0.62 |
| C38:7 PC plasmalogen | 1 (0.9-1.11) | 0.97 | 1.03 (0.89-1.19) | 0.68 | 0.99 (0.88-1.12) | 0.92 | 0.94 (0.81-1.1) | 0.48 | 0.92 (0.82-1.03) | 0.17 | 0.95 (0.78-1.16) | 0.62 |
| C38:7 PE plasmalogen | 1.04 (0.93-1.16) | 0.46 | 1.1 (0.95-1.28) | 0.21 | 1.06 (0.93-1.21) | 0.35 | 1 (0.84-1.18) | 0.99 | 1.02 (0.9-1.14) | 0.80 | 0.94 (0.75-1.19) | 0.62 |
| C40:10 PC | 0.95 (0.85-1.06) | 0.35 | 1.02 (0.88-1.19) | 0.80 | 0.99 (0.87-1.13) | 0.94 | 0.87 (0.74-1.03) | 0.10 | 0.95 (0.85-1.07) | 0.40 | 0.95 (0.77-1.17) | 0.63 |
| C40:6 PC | 0.99 (0.89-1.11) | 0.91 | 1.07 (0.91-1.25) | 0.42 | 1.03 (0.9-1.17) | 0.69 | 0.95 (0.81-1.11) | 0.50 | 0.96 (0.85-1.08) | 0.49 | 0.95 (0.78-1.17) | 0.63 |
| C40:7 PE plasmalogen | 1.08 (0.97-1.21) | 0.14 | 1.12 (0.97-1.3) | 0.12 | 1.1 (0.97-1.25) | 0.15 | 1.05 (0.89-1.23) | 0.58 | 1.07 (0.95-1.2) | 0.27 | 0.95 (0.76-1.18) | 0.63 |
| C40:9 PC | 1.01 (0.91-1.14) | 0.80 | 1.1 (0.94-1.28) | 0.25 | 1.04 (0.91-1.19) | 0.57 | 0.94 (0.79-1.11) | 0.44 | 0.99 (0.87-1.11) | 0.82 | 0.95 (0.76-1.18) | 0.64 |
| C43:0 TAG | 0.99 (0.88-1.1) | 0.80 | 0.91 (0.78-1.06) | 0.21 | 0.93 (0.81-1.06) | 0.26 | 0.98 (0.84-1.14) | 0.80 | 0.92 (0.82-1.03) | 0.15 | 1.05 (0.85-1.29) | 0.64 |
| C43:1 TAG | 0.94 (0.84-1.05) | 0.30 | 0.89 (0.76-1.04) | 0.15 | 0.89 (0.77-1.01) | 0.08 | 0.95 (0.82-1.11) | 0.54 | 0.86 (0.77-0.97) | 0.02 | 1.05 (0.85-1.29) | 0.64 |
| C43:2 TAG | 0.94 (0.84-1.06) | 0.33 | 0.89 (0.75-1.05) | 0.16 | 0.91 (0.79-1.05) | 0.20 | 0.95 (0.81-1.11) | 0.51 | 0.88 (0.78-1) | 0.05 | 0.95 (0.77-1.17) | 0.64 |
| C44:0 TAG | 0.95 (0.85-1.07) | 0.41 | 0.88 (0.76-1.03) | 0.10 | 0.9 (0.79-1.03) | 0.13 | 0.95 (0.82-1.11) | 0.55 | 0.87 (0.77-0.99) | 0.03 | 1.05 (0.85-1.3) | 0.65 |
| C45:1 TAG | 0.97 (0.86-1.08) | 0.53 | 0.89 (0.76-1.04) | 0.16 | 0.89 (0.78-1.02) | 0.10 | 0.97 (0.83-1.13) | 0.69 | 0.88 (0.78-0.99) | 0.03 | 1.05 (0.85-1.3) | 0.65 |
| C45:2 TAG | 0.95 (0.85-1.07) | 0.41 | 0.92 (0.79-1.08) | 0.32 | 0.92 (0.8-1.06) | 0.24 | 0.96 (0.82-1.12) | 0.60 | 0.9 (0.79-1.01) | 0.08 | 0.96 (0.78-1.18) | 0.67 |
| C46:0 TAG | 0.93 (0.83-1.04) | 0.21 | 0.87 (0.74-1.01) | 0.07 | 0.89 (0.78-1.02) | 0.09 | 0.94 (0.81-1.1) | 0.48 | 0.87 (0.77-0.98) | 0.02 | 0.96 (0.78-1.17) | 0.67 |
| C46:1 TAG | 0.94 (0.84-1.05) | 0.24 | 0.88 (0.75-1.02) | 0.09 | 0.89 (0.78-1.02) | 0.10 | 0.95 (0.81-1.1) | 0.48 | 0.85 (0.76-0.96) | 0.01 | 0.95 (0.77-1.19) | 0.68 |
| C46:2 TAG | 0.93 (0.83-1.04) | 0.21 | 0.89 (0.76-1.03) | 0.13 | 0.91 (0.79-1.04) | 0.18 | 0.94 (0.81-1.1) | 0.43 | 0.85 (0.76-0.96) | 0.01 | 0.96 (0.78-1.18) | 0.68 |
| C47:1 TAG | 0.99 (0.89-1.11) | 0.87 | 0.94 (0.81-1.09) | 0.41 | 0.91 (0.8-1.04) | 0.17 | 1.01 (0.86-1.18) | 0.93 | 0.9 (0.8-1.01) | 0.08 | 0.96 (0.77-1.18) | 0.68 |
| C47:2 TAG | 0.96 (0.86-1.08) | 0.50 | 0.92 (0.79-1.07) | 0.30 | 0.91 (0.8-1.04) | 0.17 | 0.97 (0.83-1.13) | 0.67 | 0.88 (0.78-0.99) | 0.03 | 0.95 (0.76-1.19) | 0.68 |
| C48:0 TAG | 0.93 (0.83-1.05) | 0.25 | 0.89 (0.76-1.05) | 0.17 | 0.89 (0.77-1.02) | 0.09 | 0.96 (0.82-1.12) | 0.58 | 0.86 (0.76-0.97) | 0.02 | 0.95 (0.77-1.19) | 0.68 |
| C48:1 TAG | 0.93 (0.83-1.04) | 0.18 | 0.9 (0.77-1.05) | 0.19 | 0.91 (0.79-1.04) | 0.17 | 0.93 (0.79-1.08) | 0.33 | 0.85 (0.75-0.96) | 0.01 | 0.96 (0.77-1.19) | 0.68 |
| C48:2 TAG | 0.94 (0.83-1.05) | 0.25 | 0.89 (0.76-1.04) | 0.16 | 0.91 (0.79-1.04) | 0.16 | 0.94 (0.8-1.1) | 0.44 | 0.85 (0.76-0.96) | 0.01 | 1.04 (0.85-1.27) | 0.69 |
| C48:3 TAG | 0.91 (0.82-1.02) | 0.12 | 0.88 (0.75-1.03) | 0.11 | 0.93 (0.81-1.07) | 0.31 | 0.9 (0.77-1.05) | 0.18 | 0.85 (0.75-0.96) | 0.01 | 0.96 (0.77-1.19) | 0.69 |
| C49:2 TAG | 0.97 (0.87-1.08) | 0.55 | 0.95 (0.82-1.1) | 0.47 | 0.92 (0.81-1.04) | 0.19 | 0.99 (0.85-1.15) | 0.89 | 0.89 (0.8-1) | 0.06 | 0.96 (0.77-1.19) | 0.69 |
| C49:3 TAG | 0.96 (0.86-1.08) | 0.52 | 0.97 (0.83-1.13) | 0.69 | 0.95 (0.84-1.08) | 0.45 | 0.98 (0.84-1.15) | 0.81 | 0.91 (0.81-1.02) | 0.10 | 0.96 (0.76-1.2) | 0.69 |
| C50:0 TAG | 0.92 (0.82-1.03) | 0.14 | 0.89 (0.76-1.04) | 0.15 | 0.88 (0.76-1.01) | 0.06 | 0.94 (0.8-1.1) | 0.43 | 0.85 (0.75-0.96) | 0.01 | 0.96 (0.79-1.17) | 0.69 |
| C50:1 TAG | 0.91 (0.81-1.03) | 0.12 | 0.9 (0.76-1.07) | 0.24 | 0.9 (0.78-1.04) | 0.17 | 0.9 (0.77-1.06) | 0.23 | 0.84 (0.74-0.95) | 0.01 | 1.04 (0.85-1.27) | 0.69 |
| C50:2 TAG | 0.9 (0.8-1.02) | 0.10 | 0.88 (0.74-1.05) | 0.15 | 0.9 (0.78-1.04) | 0.15 | 0.92 (0.78-1.08) | 0.29 | 0.84 (0.74-0.95) | 0.01 | 1.04 (0.85-1.29) | 0.70 |
| C50:3 TAG | 0.93 (0.83-1.04) | 0.22 | 0.91 (0.77-1.07) | 0.25 | 0.98 (0.85-1.13) | 0.78 | 0.92 (0.78-1.08) | 0.32 | 0.89 (0.79-1.01) | 0.07 | 0.96 (0.79-1.18) | 0.70 |
| C50:4 TAG | 0.91 (0.81-1.02) | 0.10 | 0.91 (0.78-1.07) | 0.26 | 1.01 (0.88-1.16) | 0.84 | 0.86 (0.73-1) | 0.05 | 0.89 (0.79-1) | 0.06 | 1.04 (0.84-1.3) | 0.70 |
| C50:5 TAG | 0.89 (0.79-0.99) | 0.04 | 0.88 (0.75-1.03) | 0.10 | 0.99 (0.86-1.13) | 0.85 | 0.83 (0.71-0.97) | 0.02 | 0.87 (0.77-0.98) | 0.02 | 1.04 (0.84-1.3) | 0.70 |
| C50:6 TAG | 0.9 (0.8-1) | 0.06 | 0.89 (0.76-1.04) | 0.15 | 0.97 (0.85-1.12) | 0.71 | 0.85 (0.72-0.99) | 0.03 | 0.88 (0.78-0.99) | 0.03 | 0.96 (0.77-1.19) | 0.71 |
| C51:0 TAG | 0.95 (0.85-1.07) | 0.42 | 0.92 (0.79-1.07) | 0.29 | 0.89 (0.78-1.02) | 0.09 | 0.98 (0.84-1.15) | 0.79 | 0.89 (0.79-1) | 0.05 | 0.96 (0.78-1.18) | 0.71 |
| C51:1 TAG | 0.94 (0.84-1.05) | 0.25 | 0.93 (0.79-1.09) | 0.37 | 0.9 (0.78-1.03) | 0.11 | 0.95 (0.81-1.11) | 0.51 | 0.85 (0.75-0.96) | 0.01 | 0.96 (0.77-1.19) | 0.72 |
| C51:2 TAG | 0.95 (0.85-1.06) | 0.35 | 0.94 (0.81-1.09) | 0.42 | 0.89 (0.78-1.01) | 0.08 | 0.97 (0.83-1.13) | 0.67 | 0.86 (0.77-0.97) | 0.01 | 0.96 (0.78-1.18) | 0.72 |
| C51:3 TAG | 0.96 (0.86-1.08) | 0.50 | 1.01 (0.86-1.19) | 0.89 | 1 (0.88-1.14) | 0.99 | 0.97 (0.83-1.13) | 0.67 | 0.93 (0.83-1.05) | 0.25 | 1.04 (0.84-1.29) | 0.72 |
| C52:0 TAG | 0.92 (0.82-1.04) | 0.18 | 0.89 (0.76-1.04) | 0.15 | 0.88 (0.77-1.01) | 0.08 | 0.94 (0.8-1.11) | 0.48 | 0.87 (0.77-0.98) | 0.02 | 0.96 (0.79-1.18) | 0.72 |
| C52:1 TAG | 0.9 (0.8-1.01) | 0.07 | 0.89 (0.75-1.05) | 0.17 | 0.88 (0.76-1.01) | 0.07 | 0.88 (0.75-1.03) | 0.11 | 0.83 (0.73-0.94) | 0.00 | 0.96 (0.79-1.18) | 0.72 |
| C52:2 TAG | 0.93 (0.83-1.05) | 0.24 | 0.95 (0.8-1.12) | 0.52 | 0.96 (0.84-1.11) | 0.58 | 0.88 (0.75-1.04) | 0.14 | 0.88 (0.78-0.99) | 0.04 | 0.96 (0.77-1.19) | 0.73 |
| C52:3 TAG | 0.95 (0.85-1.06) | 0.38 | 0.99 (0.84-1.16) | 0.86 | 1.03 (0.9-1.17) | 0.69 | 0.87 (0.74-1.03) | 0.10 | 0.93 (0.82-1.05) | 0.23 | 0.96 (0.78-1.19) | 0.73 |
| C52:4 TAG | 0.94 (0.84-1.05) | 0.27 | 0.98 (0.84-1.14) | 0.78 | 1.08 (0.95-1.22) | 0.25 | 0.88 (0.75-1.03) | 0.11 | 0.98 (0.87-1.1) | 0.70 | 0.96 (0.78-1.19) | 0.73 |
| C52:5 TAG | 0.94 (0.84-1.04) | 0.24 | 0.98 (0.84-1.14) | 0.75 | 1.08 (0.95-1.22) | 0.26 | 0.88 (0.75-1.03) | 0.10 | 0.99 (0.88-1.11) | 0.84 | 0.96 (0.77-1.19) | 0.73 |
| C52:6 TAG | 0.9 (0.8-1) | 0.06 | 0.91 (0.78-1.07) | 0.25 | 1.05 (0.92-1.19) | 0.49 | 0.8 (0.68-0.93) | 0.01 | 0.93 (0.82-1.04) | 0.21 | 0.96 (0.78-1.19) | 0.73 |
| C52:7 TAG | 0.9 (0.8-1) | 0.05 | 0.91 (0.78-1.06) | 0.22 | 1.01 (0.89-1.16) | 0.84 | 0.81 (0.69-0.94) | 0.01 | 0.91 (0.81-1.02) | 0.11 | 1.04 (0.84-1.28) | 0.74 |
| C53:2 TAG | 0.94 (0.84-1.06) | 0.31 | 0.99 (0.85-1.16) | 0.94 | 0.94 (0.82-1.07) | 0.34 | 0.93 (0.79-1.09) | 0.38 | 0.89 (0.79-1) | 0.06 | 1.04 (0.84-1.28) | 0.74 |
| C53:3 TAG | 0.96 (0.86-1.08) | 0.52 | 1.03 (0.89-1.21) | 0.67 | 1.02 (0.9-1.17) | 0.74 | 0.95 (0.82-1.1) | 0.51 | 0.96 (0.85-1.07) | 0.46 | 0.96 (0.77-1.2) | 0.74 |
| C54:1 TAG | 0.93 (0.83-1.04) | 0.21 | 0.91 (0.78-1.06) | 0.25 | 0.92 (0.8-1.06) | 0.24 | 0.92 (0.78-1.09) | 0.33 | 0.9 (0.79-1.01) | 0.08 | 1.04 (0.84-1.28) | 0.75 |
| C54:2 TAG | 0.93 (0.83-1.04) | 0.23 | 0.92 (0.79-1.08) | 0.32 | 0.96 (0.84-1.09) | 0.50 | 0.9 (0.76-1.06) | 0.22 | 0.91 (0.81-1.03) | 0.14 | 0.96 (0.77-1.2) | 0.75 |
| C54:3 TAG | 0.98 (0.88-1.09) | 0.68 | 0.98 (0.85-1.14) | 0.82 | 1.05 (0.93-1.19) | 0.43 | 0.92 (0.78-1.08) | 0.32 | 1.02 (0.91-1.14) | 0.76 | 0.97 (0.78-1.19) | 0.75 |
| C54:4 TAG | 0.98 (0.88-1.1) | 0.78 | 0.99 (0.85-1.15) | 0.85 | 1.08 (0.95-1.22) | 0.23 | 0.94 (0.8-1.11) | 0.46 | 1.05 (0.94-1.18) | 0.41 | 1.03 (0.84-1.27) | 0.76 |
| C54:5 TAG | 0.95 (0.84-1.06) | 0.34 | 0.91 (0.77-1.07) | 0.24 | 0.96 (0.84-1.1) | 0.56 | 0.87 (0.75-1.02) | 0.10 | 0.89 (0.79-1.01) | 0.06 | 0.97 (0.78-1.2) | 0.77 |
| C54:6 TAG | 0.95 (0.85-1.05) | 0.32 | 0.97 (0.84-1.13) | 0.73 | 1.06 (0.94-1.21) | 0.33 | 0.9 (0.77-1.05) | 0.17 | 1.02 (0.91-1.14) | 0.74 | 0.97 (0.79-1.19) | 0.77 |
| C54:7 TAG | 0.91 (0.82-1.01) | 0.08 | 0.94 (0.81-1.08) | 0.37 | 1.05 (0.93-1.19) | 0.40 | 0.81 (0.69-0.95) | 0.01 | 0.97 (0.87-1.09) | 0.62 | 1.03 (0.84-1.26) | 0.77 |
| C54:8 TAG | 0.9 (0.81-1) | 0.05 | 0.93 (0.8-1.07) | 0.30 | 1.03 (0.91-1.17) | 0.63 | 0.8 (0.68-0.94) | 0.01 | 0.93 (0.83-1.05) | 0.25 | 0.97 (0.78-1.2) | 0.77 |
| C54:9 TAG | 0.93 (0.83-1.04) | 0.20 | 0.96 (0.82-1.11) | 0.57 | 1.03 (0.91-1.17) | 0.63 | 0.83 (0.7-0.97) | 0.02 | 0.95 (0.85-1.07) | 0.41 | 1.03 (0.84-1.27) | 0.77 |
| C55:2 TAG | 0.95 (0.85-1.06) | 0.36 | 0.95 (0.81-1.11) | 0.53 | 0.93 (0.81-1.06) | 0.29 | 0.95 (0.81-1.11) | 0.50 | 0.9 (0.79-1.01) | 0.07 | 1.03 (0.84-1.27) | 0.78 |
| C55:3 TAG | 0.99 (0.89-1.11) | 0.92 | 1.02 (0.88-1.19) | 0.79 | 1 (0.88-1.14) | 0.95 | 1.04 (0.9-1.2) | 0.60 | 1.02 (0.91-1.14) | 0.71 | 1.03 (0.83-1.28) | 0.78 |
| C56:1 TAG | 0.94 (0.84-1.05) | 0.28 | 0.92 (0.79-1.08) | 0.31 | 0.96 (0.84-1.1) | 0.54 | 0.92 (0.78-1.08) | 0.31 | 0.93 (0.82-1.04) | 0.21 | 0.97 (0.78-1.2) | 0.78 |
| C56:10 TAG | 0.95 (0.85-1.06) | 0.34 | 0.98 (0.84-1.14) | 0.82 | 1.05 (0.92-1.19) | 0.50 | 0.83 (0.7-0.97) | 0.02 | 0.98 (0.87-1.1) | 0.70 | 1.03 (0.83-1.29) | 0.79 |
| C56:2 TAG | 0.96 (0.86-1.07) | 0.47 | 0.98 (0.84-1.14) | 0.75 | 0.98 (0.85-1.12) | 0.73 | 0.93 (0.79-1.09) | 0.36 | 0.94 (0.84-1.06) | 0.34 | 0.97 (0.78-1.21) | 0.79 |
| C56:3 TAG | 0.97 (0.87-1.08) | 0.61 | 0.98 (0.84-1.14) | 0.76 | 1.02 (0.9-1.16) | 0.75 | 0.94 (0.8-1.1) | 0.43 | 0.98 (0.87-1.1) | 0.73 | 0.97 (0.77-1.22) | 0.79 |
| C56:4 TAG | 1.01 (0.91-1.12) | 0.88 | 1.03 (0.89-1.2) | 0.68 | 1.09 (0.96-1.23) | 0.17 | 0.97 (0.83-1.13) | 0.71 | 1.04 (0.93-1.16) | 0.53 | 0.97 (0.8-1.19) | 0.80 |
| C56:5 TAG | 1.1 (0.98-1.23) | 0.09 | 1.15 (0.99-1.34) | 0.06 | 1.13 (0.99-1.28) | 0.07 | 1.01 (0.87-1.17) | 0.89 | 1.09 (0.97-1.22) | 0.15 | 0.97 (0.78-1.21) | 0.81 |
| C56:6 TAG | 1.04 (0.93-1.16) | 0.50 | 1.05 (0.91-1.22) | 0.52 | 1.1 (0.96-1.25) | 0.16 | 0.94 (0.8-1.09) | 0.40 | 1.06 (0.94-1.19) | 0.34 | 0.97 (0.79-1.21) | 0.81 |
| C56:7 TAG | 1 (0.89-1.11) | 0.94 | 1.03 (0.88-1.19) | 0.74 | 1.13 (0.99-1.28) | 0.07 | 0.91 (0.78-1.07) | 0.26 | 1.06 (0.95-1.19) | 0.32 | 1.02 (0.83-1.26) | 0.82 |
| C56:8 TAG | 1.01 (0.91-1.13) | 0.80 | 1.06 (0.92-1.23) | 0.40 | 1.14 (1-1.29) | 0.05 | 0.94 (0.8-1.09) | 0.40 | 1.09 (0.98-1.23) | 0.13 | 0.98 (0.79-1.2) | 0.82 |
| C56:9 TAG | 0.96 (0.86-1.07) | 0.49 | 1 (0.86-1.16) | 1.00 | 1.07 (0.94-1.22) | 0.28 | 0.85 (0.72-1) | 0.05 | 1.01 (0.9-1.14) | 0.81 | 1.02 (0.83-1.26) | 0.82 |
| C58:11 TAG | 0.97 (0.87-1.09) | 0.64 | 1.01 (0.87-1.18) | 0.90 | 1.04 (0.91-1.19) | 0.56 | 0.86 (0.73-1.02) | 0.08 | 1.01 (0.89-1.13) | 0.92 | 0.97 (0.78-1.22) | 0.83 |
| C58:6 TAG | 1.04 (0.93-1.17) | 0.47 | 1.05 (0.9-1.23) | 0.52 | 1.09 (0.95-1.24) | 0.21 | 1 (0.85-1.16) | 0.96 | 1.07 (0.95-1.2) | 0.26 | 0.98 (0.79-1.21) | 0.83 |
| C58:7 TAG | 1.07 (0.96-1.2) | 0.21 | 1.11 (0.96-1.29) | 0.16 | 1.13 (0.99-1.29) | 0.07 | 1.04 (0.9-1.21) | 0.56 | 1.14 (1.02-1.28) | 0.03 | 1.02 (0.83-1.26) | 0.84 |
| C58:9 TAG | 1.05 (0.94-1.18) | 0.37 | 1.09 (0.94-1.27) | 0.27 | 1.12 (0.97-1.28) | 0.12 | 0.99 (0.85-1.16) | 0.93 | 1.13 (1.01-1.27) | 0.04 | 1.02 (0.83-1.25) | 0.84 |
| C60:12 TAG | 1.05 (0.94-1.18) | 0.37 | 1.12 (0.96-1.31) | 0.16 | 1.07 (0.94-1.23) | 0.30 | 1.01 (0.86-1.19) | 0.89 | 1.1 (0.98-1.24) | 0.12 | 0.98 (0.78-1.22) | 0.85 |
| campesterol | 0.95 (0.85-1.06) | 0.34 | 1 (0.86-1.17) | 0.97 | 0.92 (0.81-1.05) | 0.23 | 0.95 (0.81-1.1) | 0.48 | 0.91 (0.81-1.03) | 0.14 | 0.98 (0.79-1.21) | 0.85 |
| chenodeoxycholate/deoxycholate | 0.95 (0.86-1.06) | 0.36 | 1 (0.86-1.16) | 0.99 | 1.02 (0.9-1.16) | 0.78 | 0.88 (0.75-1.02) | 0.09 | 0.91 (0.81-1.02) | 0.09 | 0.98 (0.79-1.21) | 0.85 |
| cholesterol | 0.95 (0.85-1.06) | 0.35 | 1 (0.85-1.17) | 0.96 | 0.94 (0.82-1.08) | 0.37 | 0.93 (0.79-1.08) | 0.33 | 0.92 (0.81-1.03) | 0.16 | 0.98 (0.78-1.24) | 0.86 |
| CMPF | 0.99 (0.88-1.11) | 0.84 | 1.05 (0.9-1.23) | 0.52 | 1.06 (0.93-1.21) | 0.38 | 0.95 (0.81-1.11) | 0.52 | 1 (0.88-1.12) | 0.96 | 0.98 (0.8-1.2) | 0.86 |
| coenzyme Q10 | 0.99 (0.89-1.11) | 0.90 | 1.11 (0.95-1.29) | 0.19 | 1.01 (0.88-1.15) | 0.90 | 0.89 (0.77-1.03) | 0.13 | 0.96 (0.85-1.07) | 0.45 | 0.98 (0.79-1.22) | 0.87 |
| erythronate/threonate | 1.04 (0.93-1.15) | 0.52 | 1.08 (0.93-1.25) | 0.31 | 1.05 (0.93-1.2) | 0.42 | 0.98 (0.84-1.14) | 0.78 | 1.04 (0.93-1.17) | 0.49 | 0.98 (0.79-1.22) | 0.87 |
| fucose | 1.01 (0.91-1.13) | 0.80 | 1.02 (0.88-1.17) | 0.83 | 1.06 (0.93-1.21) | 0.40 | 0.96 (0.83-1.11) | 0.56 | 0.96 (0.85-1.07) | 0.46 | 0.98 (0.81-1.2) | 0.88 |
| gentisate | 0.96 (0.85-1.1) | 0.58 | 1.07 (0.9-1.27) | 0.45 | 1.02 (0.88-1.18) | 0.84 | 0.99 (0.83-1.18) | 0.90 | 1.01 (0.88-1.16) | 0.87 | 1.02 (0.82-1.26) | 0.88 |
| glutamate | 1 (0.89-1.12) | 0.99 | 0.96 (0.83-1.12) | 0.63 | 0.94 (0.83-1.07) | 0.34 | 1.14 (0.96-1.36) | 0.13 | 0.95 (0.84-1.08) | 0.44 | 1.02 (0.82-1.25) | 0.88 |
| glycerate | 1.03 (0.93-1.15) | 0.54 | 1.05 (0.9-1.23) | 0.51 | 0.98 (0.87-1.11) | 0.74 | 1.12 (0.96-1.31) | 0.16 | 1.04 (0.93-1.17) | 0.51 | 0.98 (0.79-1.22) | 0.89 |
| hexose monophosphate | 1.02 (0.91-1.13) | 0.75 | 0.99 (0.86-1.15) | 0.92 | 0.95 (0.84-1.08) | 0.45 | 1.11 (0.95-1.3) | 0.20 | 1.02 (0.9-1.14) | 0.78 | 1.02 (0.82-1.26) | 0.89 |
| homovanillate | 1.06 (0.95-1.19) | 0.33 | 1.03 (0.88-1.21) | 0.68 | 1.04 (0.92-1.19) | 0.51 | 1.02 (0.86-1.21) | 0.84 | 1.03 (0.92-1.16) | 0.62 | 0.98 (0.79-1.23) | 0.89 |
| indole-3-propionate | 1.03 (0.92-1.16) | 0.56 | 1.05 (0.9-1.23) | 0.53 | 1.04 (0.91-1.19) | 0.55 | 1.09 (0.93-1.28) | 0.27 | 1.09 (0.96-1.22) | 0.17 | 1.02 (0.82-1.26) | 0.89 |
| indoleacetate | 0.97 (0.87-1.08) | 0.61 | 0.97 (0.83-1.13) | 0.68 | 0.98 (0.86-1.11) | 0.72 | 0.88 (0.76-1.02) | 0.08 | 0.98 (0.88-1.11) | 0.79 | 1.02 (0.81-1.27) | 0.89 |
| indoxylsulfate | 0.88 (0.79-0.99) | 0.03 | 0.88 (0.76-1.02) | 0.09 | 0.93 (0.82-1.06) | 0.28 | 0.85 (0.74-0.99) | 0.03 | 0.9 (0.8-1) | 0.06 | 0.99 (0.81-1.21) | 0.91 |
| kynurenine | 0.93 (0.83-1.03) | 0.17 | 0.94 (0.81-1.09) | 0.41 | 0.95 (0.84-1.09) | 0.48 | 0.88 (0.75-1.03) | 0.12 | 0.95 (0.85-1.07) | 0.43 | 1.01 (0.82-1.25) | 0.91 |
| malonate | 0.96 (0.86-1.06) | 0.41 | 1 (0.86-1.15) | 0.97 | 0.93 (0.81-1.05) | 0.25 | 1.01 (0.87-1.17) | 0.90 | 0.93 (0.84-1.05) | 0.24 | 0.99 (0.8-1.23) | 0.93 |
| MDA | 1.06 (0.95-1.18) | 0.32 | 1.07 (0.93-1.25) | 0.34 | 1.04 (0.91-1.18) | 0.58 | 1.01 (0.87-1.18) | 0.86 | 1.03 (0.92-1.15) | 0.62 | 1.01 (0.83-1.23) | 0.93 |
| mesaconate | 1.06 (0.95-1.19) | 0.27 | 1.02 (0.88-1.19) | 0.77 | 0.99 (0.87-1.12) | 0.83 | 1.16 (1-1.35) | 0.06 | 1.08 (0.96-1.21) | 0.21 | 0.99 (0.8-1.22) | 0.93 |
| N-acetylglutamate | 1.09 (0.97-1.22) | 0.17 | 1.09 (0.93-1.27) | 0.29 | 0.96 (0.84-1.1) | 0.57 | 1.23 (1.04-1.46) | 0.02 | 1.06 (0.94-1.2) | 0.34 | 0.99 (0.8-1.22) | 0.94 |
| palmitoylethanolamide | 1.08 (0.97-1.21) | 0.14 | 1.19 (1.03-1.39) | 0.02 | 1.1 (0.97-1.25) | 0.12 | 0.94 (0.8-1.11) | 0.47 | 1.02 (0.91-1.15) | 0.72 | 1.01 (0.8-1.26) | 0.94 |
| pentose monophosphate | 0.99 (0.89-1.11) | 0.91 | 0.96 (0.82-1.11) | 0.56 | 0.99 (0.87-1.13) | 0.87 | 1.07 (0.91-1.26) | 0.43 | 1.09 (0.96-1.22) | 0.17 | 0.99 (0.81-1.21) | 0.95 |
| phenyllactate | 1.06 (0.95-1.18) | 0.34 | 1.01 (0.88-1.18) | 0.85 | 1 (0.88-1.14) | 0.97 | 1.04 (0.89-1.21) | 0.60 | 1.09 (0.98-1.23) | 0.13 | 0.99 (0.8-1.23) | 0.96 |
| quinolinate | 0.9 (0.81-1.01) | 0.06 | 0.87 (0.74-1.02) | 0.08 | 0.96 (0.84-1.09) | 0.50 | 0.94 (0.81-1.1) | 0.43 | 0.94 (0.83-1.05) | 0.26 | 1 (0.81-1.23) | 0.97 |
| suberate | 0.97 (0.87-1.08) | 0.62 | 0.94 (0.81-1.08) | 0.38 | 0.95 (0.83-1.07) | 0.40 | 1.04 (0.89-1.2) | 0.65 | 1.03 (0.92-1.16) | 0.57 | 1 (0.81-1.25) | 0.97 |
| taurodeoxycholate/taurochenodeoxycholate | 1.13 (1-1.27) | 0.05 | 1.05 (0.89-1.23) | 0.57 | 1.04 (0.91-1.2) | 0.58 | 1.24 (1.05-1.48) | 0.01 | 1.14 (1.01-1.3) | 0.04 | 1 (0.81-1.24) | 0.98 |
| threitol | 1.04 (0.93-1.16) | 0.53 | 0.98 (0.84-1.14) | 0.81 | 1.07 (0.94-1.22) | 0.31 | 1.02 (0.87-1.2) | 0.79 | 1.05 (0.93-1.18) | 0.44 | 1 (0.82-1.23) | 0.99 |
| thymine | 1.02 (0.91-1.14) | 0.75 | 1.01 (0.87-1.18) | 0.89 | 0.95 (0.84-1.08) | 0.44 | 1.13 (0.97-1.31) | 0.11 | 1.08 (0.96-1.21) | 0.19 | 1 (0.81-1.24) | 0.99 |
| uracil | 0.99 (0.88-1.11) | 0.88 | 1 (0.86-1.17) | 0.96 | 1 (0.88-1.15) | 0.95 | 1 (0.85-1.17) | 0.96 | 1.01 (0.89-1.14) | 0.90 | 1 (0.81-1.24) | 0.99 |
| xanthurenate | 0.92 (0.82-1.03) | 0.13 | 0.94 (0.81-1.09) | 0.41 | 0.95 (0.84-1.08) | 0.46 | 0.92 (0.78-1.07) | 0.29 | 0.96 (0.85-1.08) | 0.48 | 1 (0.8-1.25) | 1.00 |

Model adjusted for BMI at age 18, weight change (from age 18 to time of first blood draw), age at menarche, parity and age at first birth, breastfeeding history, family history of breast cancer in a first degree relative, personal history of benign breast disease, physical activity, alcohol intake (by quintile), and oral contraceptive use at blood collection.
