# Supplementary material for: Prospective study of circulating metabolomic profiles and breast cancer incidence among predominantly premenopausal women
Source: Br J Cancer. 2025 Sep 4;133(9):1326–34. doi: 10.1038/s41416-025-03159-2 (PMC12572396; doi:10.1038/s41416-025-03159-2)
Supplement: Supplementary file 3 [file 41416_2025_3159_MOESM3_ESM.docx]

**Supplemental Table 3.** Odds ratios and 95% confidence intervals for associations between individual metabolites (per 1 SD increase in metabolite level) and breast cancer incidence (restricting to premenopausal women at blood collection), with metabolites reaching nominal significance, in the Nurses’ Health Study II (1996-2011)

| **METABOLITE** | **Full population** **OR (95% CI)** | **P-value** | **Premenopausal population** **OR (95% CI)** | **P-value** |
| --- | --- | --- | --- | --- |
| **Taurodeoxycholate** | 1.15 (1.04-1.28) | 0.01 | 1.13 (1-1.27) | 0.05 |
| **C16:1 CE** | 0.88 (0.79-0.97) | 0.01 | 0.91 (0.81-1.02) | 0.11 |
| **C34:1 PC** | 0.87 (0.78-0.98) | 0.02 | 0.88 (0.78-0.99) | 0.03 |
| **C34:3 PC** | 0.88 (0.79-0.98) | 0.02 | 0.87 (0.77-0.97) | 0.01 |
| **C32:1 PC** | 0.88 (0.79-0.98) | 0.02 | 0.89 (0.79-1.01) | 0.07 |
| **Indoxyl sulfate** | 0.90 (0.82-1.00) | 0.04 | 0.88 (0.79-0.99) | 0.03 |

Abbreviation: CE: cholesteryl ester; PC: phosphatidylcholine; PS: phosphatidylserine; TAG: triacylglycerides; OR: odds ratio; CI: confidence interval.

Model adjusted for BMI at age 18, weight change (from age 18 to time of first blood draw), age at menarche, parity and age at first birth, breastfeeding history, family history of breast cancer in a first degree relative, personal history of benign breast disease, physical activity, alcohol intake (by quintile), and oral contraceptive use at blood collection.
