# Supplementary material for: Prospective study of circulating metabolomic profiles and breast cancer incidence among predominantly premenopausal women
Source: Br J Cancer. 2025 Sep 4;133(9):1326–34. doi: 10.1038/s41416-025-03159-2 (PMC12572396; doi:10.1038/s41416-025-03159-2)
Supplement: Supplementary file 4 [file 41416_2025_3159_MOESM4_ESM.docx]

**Supplemental Table 4.** Odds ratios and 95% confidence intervals for associations between individual metabolites (per 1 SD increase in metabolite level) and breast cancer incidence, stratified by median time since blood collection, Nurses’ Health Study (1996-2011).

|  |  | ≤ 6.5 years |  |  | > 6.5 years |  |
| --- | --- | --- | --- | --- | --- | --- |
| HMDB_ID | METABOLITE | OR.CI | p.value |  | OR.CI | p.value |
| HMDB0000510 | 2-aminoadipate | 0.95 (0.85-1.07) | 0.382 |  | 0.92 (0.8-1.05) | 0.209 |
| HMDB0000650 | 2-aminobutyrate | 1.07 (0.95-1.2) | 0.253 |  | 1.08 (0.95-1.23) | 0.26 |
| HMDB0094649 | 2-aminoheptanoate | 1.01 (0.9-1.13) | 0.918 |  | 1.04 (0.91-1.19) | 0.558 |
| HMDB0000407 | 2-hydroxy-3-methylbutyrate | 0.94 (0.84-1.05) | 0.288 |  | 0.97 (0.85-1.11) | 0.699 |
| HMDB0000317* | 2-hydroxy-3-methylpentanoate/hydroxyisocaproate | 1.05 (0.94-1.18) | 0.397 |  | 1.04 (0.91-1.18) | 0.595 |
| HMDB0000694 | 2-hydroxyglutarate | 1.13 (1.01-1.26) | 0.038 |  | 0.97 (0.85-1.11) | 0.622 |
| HMDB0000355 | 3-hydroxymethylglutarate/anhydroDglucose | 1 (0.89-1.12) | 0.963 |  | 1.04 (0.91-1.19) | 0.558 |
| HMDB0000555* | 3-methyladipate/pimelate | 1.05 (0.94-1.17) | 0.379 |  | 0.92 (0.8-1.04) | 0.185 |
| HMDB0000017 | 4-pyridoxate | 0.95 (0.85-1.07) | 0.381 |  | 0.96 (0.84-1.1) | 0.601 |
| HMDB0000448* | adipate/methylglutarate | 1.01 (0.91-1.13) | 0.816 |  | 0.99 (0.87-1.12) | 0.829 |
| HMDB0000508* | adonitol/arabitol | 1.03 (0.92-1.16) | 0.598 |  | 0.95 (0.83-1.1) | 0.505 |
| HMDB0000008* | alpha-hydroxybutyrate/beta-hydroxybutyrate/hydroxyisobutyrate | 1.02 (0.91-1.15) | 0.685 |  | 1.08 (0.94-1.24) | 0.269 |
| HMDB0000491* | alpha-keto-beta-methylvalerate/alpha-ketoisocaproate | 1.12 (1-1.26) | 0.046 |  | 1.05 (0.92-1.2) | 0.469 |
| HMDB0000019 | alpha-ketoisovalerate | 1.09 (0.98-1.22) | 0.128 |  | 1.08 (0.95-1.24) | 0.251 |
| HMDB0000191 | aspartate | 0.99 (0.89-1.11) | 0.904 |  | 1.08 (0.95-1.24) | 0.24 |
| HMDB0006725 | C14:0 CE | 0.92 (0.82-1.03) | 0.139 |  | 0.94 (0.82-1.07) | 0.332 |
| HMDB0010379 | C14:0 LPC | 0.95 (0.85-1.07) | 0.413 |  | 0.95 (0.83-1.09) | 0.456 |
| HMDB0012097 | C14:0 SM | 1 (0.89-1.13) | 0.985 |  | 0.96 (0.83-1.1) | 0.518 |
| HMDB0000885 | C16:0 CE | 0.95 (0.85-1.07) | 0.422 |  | 1.14 (1-1.31) | 0.05 |
| HMDB0004949 | C16:0 Ceramide (d18:1) | 1.03 (0.92-1.15) | 0.661 |  | 0.95 (0.83-1.08) | 0.433 |
| HMDB0010382 | C16:0 LPC | 0.97 (0.87-1.08) | 0.58 |  | 0.95 (0.84-1.08) | 0.474 |
| HMDB0011503 | C16:0 LPE | 1 (0.89-1.12) | 0.959 |  | 0.93 (0.81-1.06) | 0.288 |
| HMDB0010169 | C16:0 SM | 1.05 (0.94-1.17) | 0.4 |  | 1.03 (0.9-1.17) | 0.678 |
| HMDB0000658* | C16:1 CE | 0.9 (0.8-1.01) | 0.07 |  | 0.85 (0.74-0.97) | 0.02 |
| HMDB0010383* | C16:1 LPC | 0.96 (0.86-1.07) | 0.476 |  | 0.9 (0.79-1.02) | 0.099 |
| HMDB0010368 | C18:0 CE | 1.02 (0.91-1.14) | 0.702 |  | 1.06 (0.93-1.21) | 0.405 |
| HMDB0010384 | C18:0 LPC | 1.03 (0.92-1.15) | 0.645 |  | 1.03 (0.9-1.17) | 0.672 |
| HMDB0011130 | C18:0 LPE | 1.02 (0.91-1.14) | 0.745 |  | 0.96 (0.84-1.09) | 0.546 |
| HMDB0001348 | C18:0 SM | 1.01 (0.9-1.13) | 0.844 |  | 1.06 (0.92-1.21) | 0.431 |
| HMDB0000918* | C18:1 CE | 0.97 (0.87-1.09) | 0.653 |  | 1.08 (0.95-1.24) | 0.257 |
| HMDB0002815* | C18:1 LPC | 1 (0.89-1.13) | 0.962 |  | 0.97 (0.85-1.11) | 0.663 |
| HMDB0011506* | C18:1 LPE | 1.03 (0.91-1.15) | 0.677 |  | 0.92 (0.81-1.06) | 0.261 |
| HMDB0012101* | C18:1 SM | 1.02 (0.91-1.14) | 0.744 |  | 1.03 (0.9-1.18) | 0.628 |
| HMDB0000610* | C18:2 CE | 0.97 (0.86-1.09) | 0.579 |  | 1.13 (0.98-1.3) | 0.084 |
| HMDB0010386* | C18:2 LPC | 1.01 (0.89-1.14) | 0.855 |  | 0.98 (0.85-1.13) | 0.828 |
| HMDB0011507* | C18:2 LPE | 1.01 (0.9-1.14) | 0.832 |  | 0.92 (0.81-1.06) | 0.252 |
| HMDB0010370* | C18:3 CE | 0.93 (0.83-1.04) | 0.207 |  | 0.92 (0.81-1.05) | 0.238 |
| HMDB0010387* | C18:3 LPC | 0.99 (0.88-1.1) | 0.813 |  | 0.96 (0.84-1.09) | 0.514 |
| HMDB0012102 | C20:0 SM | 1.02 (0.9-1.14) | 0.797 |  | 1.01 (0.88-1.16) | 0.86 |
| HMDB0006736* | C20:3 CE | 0.96 (0.86-1.08) | 0.525 |  | 1.07 (0.94-1.21) | 0.322 |
| HMDB0006726 | C20:4 CE | 1.04 (0.92-1.16) | 0.545 |  | 1.09 (0.96-1.25) | 0.177 |
| HMDB0010395 | C20:4 LPC | 1.03 (0.92-1.15) | 0.627 |  | 0.98 (0.86-1.12) | 0.792 |
| HMDB0011517 | C20:4 LPE | 1.1 (0.99-1.24) | 0.084 |  | 0.91 (0.79-1.04) | 0.148 |
| HMDB0006731 | C20:5 CE | 0.95 (0.85-1.07) | 0.412 |  | 0.99 (0.87-1.13) | 0.907 |
| HMDB0010397 | C20:5 LPC | 1 (0.89-1.13) | 0.947 |  | 0.97 (0.85-1.12) | 0.717 |
| HMDB0004952 | C22:0 Ceramide (d18:1) | 1 (0.89-1.12) | 0.947 |  | 0.89 (0.78-1.02) | 0.103 |
| HMDB0011520 | C22:0 LPE | 1.01 (0.9-1.14) | 0.819 |  | 1.07 (0.93-1.22) | 0.368 |
| HMDB0012104* | C22:1 SM | 1.03 (0.92-1.15) | 0.626 |  | 0.98 (0.85-1.11) | 0.718 |
| HMDB0010375* | C22:5 CE | 0.96 (0.85-1.09) | 0.535 |  | 1.04 (0.9-1.19) | 0.601 |
| HMDB0006733 | C22:6 CE | 1.03 (0.91-1.16) | 0.618 |  | 1.22 (1.07-1.4) | 0.004 |
| HMDB0010404 | C22:6 LPC | 1 (0.89-1.12) | 0.992 |  | 1.07 (0.94-1.23) | 0.302 |
| HMDB0011526 | C22:6 LPE | 1.05 (0.93-1.18) | 0.429 |  | 1.01 (0.88-1.15) | 0.94 |
| HMDB0004956 | C24:0 Ceramide (d18:1) | 1.02 (0.91-1.14) | 0.706 |  | 0.91 (0.8-1.04) | 0.167 |
| HMDB0004953* | C24:1 Ceramide (d18:1) | 1.02 (0.91-1.14) | 0.7 |  | 0.92 (0.81-1.05) | 0.218 |
| HMDB0012107* | C24:1 SM | 1.03 (0.91-1.16) | 0.652 |  | 1.03 (0.9-1.18) | 0.653 |
| HMDB0007869* | C30:0 PC | 0.96 (0.86-1.08) | 0.488 |  | 0.93 (0.81-1.07) | 0.303 |
| HMDB0007870* | C30:1 PC | 0.96 (0.85-1.08) | 0.478 |  | 0.88 (0.77-1.01) | 0.07 |
| HMDB0007098* | C32:0 DAG | 0.95 (0.84-1.07) | 0.371 |  | 0.9 (0.78-1.03) | 0.138 |
| HMDB0007871* | C32:0 PC | 1.01 (0.89-1.13) | 0.915 |  | 0.93 (0.81-1.07) | 0.297 |
| HMDB0008923* | C32:0 PE | 0.97 (0.87-1.09) | 0.623 |  | 0.97 (0.85-1.11) | 0.645 |
| HMDB0007099* | C32:1 DAG | 0.97 (0.86-1.09) | 0.623 |  | 0.94 (0.82-1.08) | 0.392 |
| HMDB0007873* | C32:1 PC | 0.94 (0.83-1.06) | 0.3 |  | 0.82 (0.71-0.95) | 0.007 |
| HMDB0007874* | C32:2 PC | 0.98 (0.87-1.1) | 0.757 |  | 0.93 (0.81-1.07) | 0.326 |
| HMDB0007100* | C34:0 DAG | 0.97 (0.86-1.09) | 0.621 |  | 0.91 (0.79-1.04) | 0.15 |
| HMDB0008925* | C34:0 PE | 0.98 (0.87-1.1) | 0.703 |  | 0.97 (0.85-1.1) | 0.621 |
| HMDB0012356* | C34:0 PS | 0.9 (0.8-1.01) | 0.072 |  | 0.88 (0.76-1.01) | 0.06 |
| HMDB0007102* | C34:1 DAG | 0.96 (0.85-1.08) | 0.464 |  | 0.93 (0.81-1.07) | 0.3 |
| HMDB0007972* | C34:1 PC | 0.93 (0.82-1.04) | 0.213 |  | 0.8 (0.69-0.92) | 0.002 |
| HMDB0011208* | C34:1 PC plasmalogen | 1.08 (0.97-1.21) | 0.156 |  | 1.06 (0.93-1.2) | 0.409 |
| HMDB0011239* | C34:1 PC plasmalogen-B | 1.05 (0.94-1.18) | 0.362 |  | 1.1 (0.96-1.25) | 0.169 |
| HMDB0007103* | C34:2 DAG | 0.97 (0.86-1.09) | 0.637 |  | 0.96 (0.83-1.1) | 0.52 |
| HMDB0007973* | C34:2 PC | 0.98 (0.87-1.1) | 0.732 |  | 0.93 (0.81-1.06) | 0.279 |
| HMDB0011210* | C34:2 PC plasmalogen | 1.04 (0.92-1.17) | 0.529 |  | 1.06 (0.92-1.21) | 0.433 |
| HMDB0008928* | C34:2 PE | 1.01 (0.9-1.14) | 0.891 |  | 0.91 (0.79-1.04) | 0.174 |
| HMDB0008952* | C34:2 PE plasmalogen | 1.03 (0.92-1.15) | 0.632 |  | 1.04 (0.91-1.19) | 0.532 |
| HMDB0007132* | C34:3 DAG | 1.01 (0.9-1.14) | 0.87 |  | 0.99 (0.86-1.13) | 0.832 |
| HMDB0008006* | C34:3 PC | 0.94 (0.83-1.05) | 0.279 |  | 0.82 (0.72-0.94) | 0.004 |
| HMDB0011211* | C34:3 PC plasmalogen | 1.03 (0.92-1.15) | 0.638 |  | 1.08 (0.95-1.24) | 0.232 |
| HMDB0011343* | C34:3 PE plasmalogen | 1.01 (0.91-1.13) | 0.803 |  | 1.05 (0.92-1.19) | 0.501 |
| HMDB0007883* | C34:4 PC | 0.96 (0.85-1.08) | 0.499 |  | 0.9 (0.78-1.04) | 0.143 |
| HMDB0011214* | C34:5 PC plasmalogen | 1.04 (0.93-1.16) | 0.457 |  | 1.06 (0.93-1.2) | 0.364 |
| HMDB0008991* | C36:0 PE | 0.94 (0.84-1.06) | 0.325 |  | 0.99 (0.87-1.12) | 0.847 |
| HMDB0007216* | C36:1 DAG | 0.95 (0.84-1.07) | 0.375 |  | 0.88 (0.76-1.01) | 0.066 |
| HMDB0008038* | C36:1 PC | 0.95 (0.85-1.06) | 0.382 |  | 0.86 (0.76-0.98) | 0.026 |
| HMDB0008993* | C36:1 PE | 0.99 (0.88-1.12) | 0.875 |  | 0.92 (0.8-1.06) | 0.231 |
| HMDB0009016* | C36:1 PE plasmalogen | 0.99 (0.88-1.11) | 0.897 |  | 1.08 (0.94-1.24) | 0.257 |
| HMDB0007218* | C36:2 DAG | 1.02 (0.91-1.15) | 0.727 |  | 0.9 (0.78-1.03) | 0.114 |
| HMDB0008039* | C36:2 PC | 1 (0.89-1.11) | 0.977 |  | 0.97 (0.85-1.1) | 0.623 |
| HMDB0011243* | C36:2 PC plasmalogen | 1.09 (0.97-1.22) | 0.13 |  | 1.08 (0.95-1.24) | 0.231 |
| HMDB0008994* | C36:2 PE | 0.98 (0.87-1.1) | 0.75 |  | 0.91 (0.8-1.05) | 0.209 |
| HMDB0009082* | C36:2 PE plasmalogen | 1.02 (0.92-1.14) | 0.698 |  | 1.04 (0.91-1.18) | 0.572 |
| HMDB0007219* | C36:3 DAG | 1.05 (0.94-1.18) | 0.398 |  | 0.98 (0.86-1.11) | 0.733 |
| HMDB0008105* | C36:3 PC | 0.99 (0.89-1.11) | 0.899 |  | 0.91 (0.8-1.04) | 0.168 |
| HMDB0011244* | C36:3 PC plasmalogen | 1.05 (0.93-1.17) | 0.445 |  | 1.09 (0.95-1.24) | 0.211 |
| HMDB0009060* | C36:3 PE | 1.03 (0.91-1.15) | 0.675 |  | 0.94 (0.82-1.08) | 0.374 |
| HMDB0011441* | C36:3 PE plasmalogen | 1.01 (0.91-1.13) | 0.835 |  | 1.01 (0.89-1.15) | 0.852 |
| HMDB0007248* | C36:4 DAG | 1.04 (0.93-1.16) | 0.511 |  | 1.05 (0.92-1.19) | 0.507 |
| HMDB0011310* | C36:4 PC plasmalogen | 1.06 (0.95-1.19) | 0.3 |  | 1.03 (0.91-1.18) | 0.614 |
| HMDB0007983* | C36:4 PC-A | 0.96 (0.86-1.07) | 0.455 |  | 0.93 (0.82-1.06) | 0.282 |
| HMDB0008138* | C36:4 PC-B | 1.02 (0.91-1.15) | 0.731 |  | 0.95 (0.83-1.09) | 0.457 |
| HMDB0008937* | C36:4 PE | 1.03 (0.91-1.16) | 0.635 |  | 0.92 (0.8-1.06) | 0.231 |
| HMDB0011442* | C36:4 PE plasmalogen | 1.01 (0.9-1.12) | 0.917 |  | 1.03 (0.91-1.17) | 0.627 |
| HMDB0011221* | C36:5 PC plasmalogen-A | 0.96 (0.86-1.07) | 0.502 |  | 0.95 (0.84-1.08) | 0.47 |
| HMDB0011220* | C36:5 PC plasmalogen-B | 1.02 (0.91-1.13) | 0.783 |  | 1.04 (0.91-1.18) | 0.552 |
| HMDB0011410* | C36:5 PE plasmalogen | 1.06 (0.95-1.19) | 0.294 |  | 1.07 (0.94-1.22) | 0.32 |
| HMDB0008270* | C38:2 PC | 0.95 (0.85-1.07) | 0.427 |  | 0.92 (0.8-1.06) | 0.233 |
| HMDB0008942* | C38:2 PE | 0.98 (0.88-1.1) | 0.755 |  | 1.06 (0.93-1.21) | 0.366 |
| HMDB0008047* | C38:3 PC | 0.96 (0.85-1.09) | 0.535 |  | 0.93 (0.81-1.08) | 0.337 |
| HMDB0011384* | C38:3 PE plasmalogen | 0.99 (0.88-1.1) | 0.796 |  | 1.09 (0.95-1.24) | 0.213 |
| HMDB0008048* | C38:4 PC | 1.03 (0.92-1.16) | 0.603 |  | 0.99 (0.86-1.13) | 0.84 |
| HMDB0011252* | C38:4 PC plasmalogen | 1.06 (0.95-1.18) | 0.317 |  | 1.04 (0.91-1.19) | 0.525 |
| HMDB0009003* | C38:4 PE | 1.05 (0.93-1.19) | 0.421 |  | 0.92 (0.8-1.06) | 0.239 |
| HMDB0007199* | C38:5 DAG | 1.01 (0.9-1.13) | 0.89 |  | 0.96 (0.83-1.09) | 0.514 |
| HMDB0009069* | C38:5 PE | 1.06 (0.95-1.2) | 0.303 |  | 0.9 (0.79-1.03) | 0.14 |
| HMDB0011386* | C38:5 PE plasmalogen | 1.06 (0.95-1.19) | 0.288 |  | 1.05 (0.92-1.2) | 0.425 |
| HMDB0007991* | C38:6 PC | 1.02 (0.91-1.15) | 0.723 |  | 1.05 (0.91-1.2) | 0.526 |
| HMDB0009102* | C38:6 PE | 1.03 (0.91-1.16) | 0.666 |  | 0.98 (0.85-1.13) | 0.753 |
| HMDB0011387* | C38:6 PE plasmalogen | 1.03 (0.92-1.14) | 0.636 |  | 1.03 (0.91-1.17) | 0.646 |
| HMDB0011229* | C38:7 PC plasmalogen | 0.94 (0.84-1.05) | 0.273 |  | 1.08 (0.95-1.23) | 0.228 |
| HMDB0011420* | C38:7 PE plasmalogen | 1.04 (0.92-1.17) | 0.533 |  | 1.1 (0.96-1.26) | 0.158 |
| HMDB0008511* | C40:10 PC | 0.97 (0.87-1.09) | 0.666 |  | 0.96 (0.84-1.09) | 0.497 |
| HMDB0008057* | C40:6 PC | 0.99 (0.88-1.11) | 0.884 |  | 1.03 (0.9-1.18) | 0.631 |
| HMDB0011394* | C40:7 PE plasmalogen | 1.08 (0.96-1.2) | 0.209 |  | 1.13 (0.99-1.29) | 0.067 |
| HMDB0008731* | C40:9 PC | 1.01 (0.89-1.13) | 0.915 |  | 1.04 (0.91-1.2) | 0.529 |
| HMDB0042062* | C43:0 TAG | 0.96 (0.86-1.08) | 0.474 |  | 0.93 (0.82-1.07) | 0.321 |
| HMDB0042098* | C43:1 TAG | 0.92 (0.82-1.03) | 0.15 |  | 0.9 (0.79-1.04) | 0.144 |
| HMDB0043169* | C43:2 TAG | 0.93 (0.82-1.05) | 0.232 |  | 0.92 (0.8-1.06) | 0.247 |
| HMDB0042063* | C44:0 TAG | 0.94 (0.84-1.05) | 0.285 |  | 0.89 (0.78-1.02) | 0.1 |
| HMDB0042099* | C45:1 TAG | 0.93 (0.83-1.05) | 0.242 |  | 0.91 (0.8-1.05) | 0.19 |
| HMDB0043170* | C45:2 TAG | 0.94 (0.84-1.06) | 0.316 |  | 0.92 (0.8-1.06) | 0.258 |
| HMDB0010411* | C46:0 TAG | 0.93 (0.83-1.04) | 0.212 |  | 0.87 (0.76-1) | 0.051 |
| HMDB0010412* | C46:1 TAG | 0.93 (0.83-1.04) | 0.213 |  | 0.89 (0.77-1.01) | 0.08 |
| HMDB0010419* | C46:2 TAG | 0.94 (0.83-1.05) | 0.261 |  | 0.89 (0.78-1.02) | 0.107 |
| HMDB0042100* | C47:1 TAG | 0.95 (0.85-1.06) | 0.354 |  | 0.95 (0.84-1.09) | 0.465 |
| HMDB0042076* | C47:2 TAG | 0.94 (0.83-1.05) | 0.256 |  | 0.93 (0.81-1.06) | 0.282 |
| HMDB0005356* | C48:0 TAG | 0.92 (0.82-1.04) | 0.19 |  | 0.89 (0.78-1.02) | 0.105 |
| HMDB0005359* | C48:1 TAG | 0.93 (0.83-1.05) | 0.238 |  | 0.88 (0.76-1.01) | 0.061 |
| HMDB0005376* | C48:2 TAG | 0.93 (0.83-1.05) | 0.261 |  | 0.89 (0.78-1.02) | 0.099 |
| HMDB0005432* | C48:3 TAG | 0.93 (0.83-1.05) | 0.235 |  | 0.89 (0.77-1.02) | 0.091 |
| HMDB0011706* | C49:2 TAG | 0.94 (0.84-1.05) | 0.304 |  | 0.95 (0.83-1.08) | 0.438 |
| HMDB0042103* | C49:3 TAG | 0.96 (0.86-1.08) | 0.489 |  | 0.96 (0.84-1.09) | 0.502 |
| HMDB0005357* | C50:0 TAG | 0.92 (0.81-1.03) | 0.148 |  | 0.87 (0.76-1) | 0.053 |
| HMDB0005360* | C50:1 TAG | 0.93 (0.82-1.05) | 0.242 |  | 0.86 (0.75-0.99) | 0.042 |
| HMDB0005377* | C50:2 TAG | 0.94 (0.83-1.06) | 0.306 |  | 0.86 (0.74-0.99) | 0.034 |
| HMDB0005433* | C50:3 TAG | 0.98 (0.87-1.1) | 0.744 |  | 0.9 (0.79-1.04) | 0.143 |
| HMDB0005435* | C50:4 TAG | 0.97 (0.86-1.09) | 0.564 |  | 0.9 (0.78-1.03) | 0.128 |
| HMDB0010471* | C50:5 TAG | 0.94 (0.84-1.05) | 0.285 |  | 0.87 (0.76-1) | 0.052 |
| HMDB0010497* | C50:6 TAG | 0.93 (0.83-1.04) | 0.203 |  | 0.9 (0.78-1.04) | 0.143 |
| HMDB0031106* | C51:0 TAG | 0.93 (0.83-1.04) | 0.202 |  | 0.91 (0.8-1.05) | 0.191 |
| HMDB0042104* | C51:1 TAG | 0.91 (0.81-1.02) | 0.112 |  | 0.94 (0.82-1.07) | 0.354 |
| HMDB0005362* | C51:2 TAG | 0.92 (0.82-1.03) | 0.154 |  | 0.93 (0.81-1.06) | 0.257 |
| HMDB0011701* | C51:3 TAG | 0.98 (0.88-1.1) | 0.76 |  | 0.98 (0.86-1.12) | 0.821 |
| HMDB0005365* | C52:0 TAG | 0.94 (0.83-1.06) | 0.289 |  | 0.85 (0.74-0.98) | 0.025 |
| HMDB0005367* | C52:1 TAG | 0.9 (0.8-1.02) | 0.108 |  | 0.83 (0.72-0.96) | 0.013 |
| HMDB0005369* | C52:2 TAG | 0.97 (0.86-1.1) | 0.662 |  | 0.87 (0.75-1) | 0.045 |
| HMDB0005384* | C52:3 TAG | 1 (0.89-1.12) | 0.988 |  | 0.91 (0.8-1.05) | 0.194 |
| HMDB0005363* | C52:4 TAG | 1 (0.9-1.12) | 0.97 |  | 0.98 (0.86-1.11) | 0.731 |
| HMDB0005380* | C52:5 TAG | 1.02 (0.91-1.14) | 0.766 |  | 0.95 (0.83-1.08) | 0.446 |
| HMDB0005436* | C52:6 TAG | 0.97 (0.87-1.09) | 0.649 |  | 0.9 (0.78-1.03) | 0.115 |
| HMDB0010517* | C52:7 TAG | 0.95 (0.85-1.06) | 0.351 |  | 0.9 (0.78-1.03) | 0.134 |
| HMDB0042196* | C53:2 TAG | 0.95 (0.84-1.06) | 0.349 |  | 0.94 (0.82-1.07) | 0.361 |
| HMDB0043058* | C53:3 TAG | 0.99 (0.89-1.11) | 0.869 |  | 1.01 (0.89-1.15) | 0.911 |
| HMDB0005395* | C54:1 TAG | 0.95 (0.85-1.07) | 0.437 |  | 0.87 (0.75-1) | 0.047 |
| HMDB0005403* | C54:2 TAG | 0.98 (0.87-1.1) | 0.727 |  | 0.86 (0.75-0.99) | 0.032 |
| HMDB0005405* | C54:3 TAG | 1.05 (0.94-1.17) | 0.394 |  | 0.92 (0.81-1.05) | 0.234 |
| HMDB0005370* | C54:4 TAG | 1.05 (0.94-1.18) | 0.357 |  | 0.97 (0.85-1.1) | 0.651 |
| HMDB0005385* | C54:5 TAG | 0.94 (0.84-1.06) | 0.335 |  | 0.9 (0.78-1.04) | 0.149 |
| HMDB0005391* | C54:6 TAG | 1 (0.9-1.12) | 0.943 |  | 0.97 (0.85-1.1) | 0.656 |
| HMDB0005447* | C54:7 TAG | 0.97 (0.87-1.08) | 0.529 |  | 0.95 (0.84-1.08) | 0.458 |
| HMDB0010518* | C54:8 TAG | 0.95 (0.85-1.06) | 0.336 |  | 0.94 (0.82-1.07) | 0.351 |
| HMDB0010498* | C54:9 TAG | 0.95 (0.84-1.06) | 0.334 |  | 0.97 (0.85-1.11) | 0.684 |
| HMDB0042226* | C55:2 TAG | 0.97 (0.86-1.09) | 0.579 |  | 0.9 (0.78-1.03) | 0.122 |
| HMDB0042466* | C55:3 TAG | 1.03 (0.92-1.15) | 0.613 |  | 1.01 (0.89-1.14) | 0.889 |
| HMDB0005396* | C56:1 TAG | 0.96 (0.86-1.08) | 0.539 |  | 0.91 (0.79-1.05) | 0.196 |
| HMDB0010513* | C56:10 TAG | 0.96 (0.85-1.07) | 0.435 |  | 0.99 (0.86-1.13) | 0.85 |
| HMDB0005404* | C56:2 TAG | 0.98 (0.87-1.1) | 0.754 |  | 0.92 (0.8-1.05) | 0.216 |
| HMDB0005410* | C56:3 TAG | 1.04 (0.93-1.16) | 0.511 |  | 0.91 (0.8-1.04) | 0.163 |
| HMDB0005398* | C56:4 TAG | 1.07 (0.96-1.2) | 0.211 |  | 1 (0.88-1.13) | 0.973 |
| HMDB0005406* | C56:5 TAG | 1.09 (0.97-1.22) | 0.134 |  | 1.09 (0.95-1.24) | 0.216 |
| HMDB0005456* | C56:6 TAG | 1.04 (0.93-1.16) | 0.524 |  | 1.04 (0.91-1.18) | 0.571 |
| HMDB0005462* | C56:7 TAG | 1.04 (0.93-1.16) | 0.522 |  | 1.05 (0.92-1.2) | 0.444 |
| HMDB0005392* | C56:8 TAG | 1.02 (0.91-1.14) | 0.744 |  | 1.13 (0.99-1.28) | 0.07 |
| HMDB0005448* | C56:9 TAG | 0.97 (0.87-1.09) | 0.606 |  | 1.03 (0.91-1.18) | 0.614 |
| HMDB0010531* | C58:11 TAG | 0.95 (0.85-1.07) | 0.408 |  | 1.04 (0.91-1.19) | 0.566 |
| HMDB0005458* | C58:6 TAG | 1.05 (0.94-1.18) | 0.378 |  | 1.07 (0.94-1.22) | 0.314 |
| HMDB0005471* | C58:7 TAG | 1.11 (0.99-1.24) | 0.082 |  | 1.09 (0.96-1.24) | 0.162 |
| HMDB0005463* | C58:9 TAG | 1.03 (0.92-1.16) | 0.574 |  | 1.14 (1-1.31) | 0.051 |
| HMDB0005478* | C60:12 TAG | 1.01 (0.9-1.13) | 0.917 |  | 1.16 (1.01-1.33) | 0.033 |
| HMDB0002869 | campesterol | 0.98 (0.87-1.1) | 0.701 |  | 0.89 (0.77-1.01) | 0.078 |
| HMDB0000626 | chenodeoxycholate/deoxycholate | 0.96 (0.86-1.07) | 0.467 |  | 0.97 (0.85-1.1) | 0.608 |
| HMDB0000067 | cholesterol | 0.98 (0.87-1.1) | 0.734 |  | 0.89 (0.78-1.02) | 0.098 |
| HMDB0061112 | CMPF | 1.01 (0.9-1.13) | 0.891 |  | 1.07 (0.93-1.22) | 0.339 |
| HMDB0001072 | coenzyme Q10 | 0.95 (0.84-1.06) | 0.328 |  | 1 (0.88-1.14) | 0.954 |
| HMDB0000613* | erythronate/threonate | 0.97 (0.86-1.08) | 0.542 |  | 1.08 (0.94-1.23) | 0.262 |
| HMDB0000174 | fucose | 1.04 (0.93-1.16) | 0.502 |  | 0.96 (0.84-1.1) | 0.59 |
| HMDB0000152 | gentisate | 0.97 (0.85-1.1) | 0.614 |  | 1.04 (0.89-1.2) | 0.644 |
| HMDB0000148 | glutamate | 1.02 (0.9-1.14) | 0.794 |  | 1 (0.87-1.15) | 0.97 |
| HMDB0000139 | glycerate | 1.01 (0.9-1.13) | 0.881 |  | 1.06 (0.93-1.21) | 0.377 |
| HMDB0001401* | hexose monophosphate | 0.95 (0.85-1.06) | 0.388 |  | 1.15 (1-1.32) | 0.043 |
| HMDB0000118 | homovanillate | 1.1 (0.98-1.23) | 0.106 |  | 0.93 (0.82-1.07) | 0.325 |
| HMDB0002302 | indole-3-propionate | 1.06 (0.95-1.19) | 0.295 |  | 1.06 (0.93-1.22) | 0.393 |
| HMDB0000197 | indoleacetate | 0.91 (0.81-1.02) | 0.102 |  | 0.97 (0.85-1.1) | 0.624 |
| HMDB0000682 | indoxylsulfate | 0.86 (0.77-0.96) | 0.006 |  | 0.96 (0.84-1.09) | 0.494 |
| HMDB0000684 | kynurenine | 0.96 (0.86-1.08) | 0.537 |  | 0.86 (0.76-0.99) | 0.031 |
| HMDB0000691 | malonate | 0.99 (0.89-1.11) | 0.902 |  | 0.9 (0.79-1.02) | 0.112 |
| HMDB0006112 | MDA | 1.02 (0.91-1.14) | 0.778 |  | 1.09 (0.95-1.24) | 0.203 |
| HMDB0000749 | mesaconate | 1.01 (0.91-1.13) | 0.819 |  | 1.11 (0.97-1.27) | 0.121 |
| HMDB0001138 | N-acetylglutamate | 1.04 (0.92-1.17) | 0.526 |  | 1.07 (0.92-1.23) | 0.369 |
| HMDB0002100 | palmitoylethanolamide | 1.08 (0.97-1.21) | 0.167 |  | 1.02 (0.9-1.17) | 0.742 |
| HMDB0001548 | pentose monophosphate | 1 (0.89-1.13) | 0.992 |  | 1.06 (0.93-1.21) | 0.391 |
| HMDB0000779 | phenyllactate | 1.09 (0.97-1.22) | 0.159 |  | 0.95 (0.83-1.08) | 0.441 |
| HMDB0000232 | quinolinate | 0.94 (0.84-1.05) | 0.284 |  | 0.93 (0.81-1.06) | 0.273 |
| HMDB0000893 | suberate | 0.99 (0.89-1.1) | 0.841 |  | 0.97 (0.86-1.11) | 0.669 |
| HMDB0000896* | taurodeoxycholate/taurochenodeoxycholate | 1.12 (0.99-1.27) | 0.065 |  | 1.1 (0.96-1.27) | 0.175 |
| HMDB0004136 | threitol | 1.03 (0.92-1.16) | 0.558 |  | 1.04 (0.91-1.2) | 0.533 |
| HMDB0000262 | thymine | 1.04 (0.93-1.17) | 0.458 |  | 1 (0.88-1.14) | 0.986 |
| HMDB0000300 | uracil | 0.99 (0.88-1.11) | 0.834 |  | 1.08 (0.94-1.24) | 0.275 |
| HMDB0000881 | xanthurenate | 0.91 (0.81-1.01) | 0.089 |  | 0.99 (0.87-1.13) | 0.911 |

Model adjusted for BMI at age 18, weight change (from age 18 to time of first blood draw), age at menarche, parity and age at first birth, breastfeeding history, family history of breast cancer in a first degree relative, personal history of benign breast disease, physical activity, alcohol intake (by quintile), and oral contraceptive use at blood collection
