# Supplementary material for: Prospective study of circulating metabolomic profiles and breast cancer incidence among predominantly premenopausal women
Source: Br J Cancer. 2025 Sep 4;133(9):1326–34. doi: 10.1038/s41416-025-03159-2 (PMC12572396; doi:10.1038/s41416-025-03159-2)
Supplement: Supplementary file 5 [file 41416_2025_3159_MOESM5_ESM.docx]

**Supplemental Table 5.** Metabolite set enrichment analysis (MSEA) by class of metabolites (n=218) for breast cancer incidence stratified by subgroups, Nurses’ Health Study II (1996-2011).
