# Supplementary material for: Prospective study of circulating metabolomic profiles and breast cancer incidence among predominantly premenopausal women
Source: Br J Cancer. 2025 Sep 4;133(9):1326–34. doi: 10.1038/s41416-025-03159-2 (PMC12572396; doi:10.1038/s41416-025-03159-2)
Supplement: Supplementary file 8 [file 41416_2025_3159_MOESM8_ESM.docx]

**Supplementary Figure 1: WGCNA module loadings for breast cancer.** Shown are loadings from modules associated with breast cancer at FDR<0.2. Loadings/module colors correspond to colors in Suppl Table 6.
