# Supplementary material for: Prospective study of circulating metabolomic profiles and breast cancer incidence among predominantly premenopausal women
Source: Br J Cancer. 2025 Sep 4;133(9):1326–34. doi: 10.1038/s41416-025-03159-2 (PMC12572396; doi:10.1038/s41416-025-03159-2)
Supplement: Supplementary file 9 [file 41416_2025_3159_MOESM9_ESM.docx]

**Supplemental Methods**

***Selection of breast cancer cases and matched controls***

As described in the main text, we conducted a nested case-control study within the NHSII. Eligible women for this study provided a blood sample as part of the blood sub-cohort and were free of cancer (except for nonmelanoma skin cancer) at the time of blood collection. 

Due to cohort administrative policies, cases and controls included in the present study were also free of any other cancer (other than nonmelanoma skin cancer) at the time of their selection as a case or a control. This additional restriction on the study population could introduce selection bias if (i) the metabolites affect other cancers or (ii) the metabolites share common causes (e.g., genetic factors) with other cancers (see figures below).

a)                                                                            b) 

 

Directed acyclic graph depicting potential selection bias arising from the restriction of the study to cases and controls free of other cancers at the time of their selection. a) Scenario in which the metabolites directly affect the risk of developing other cancers. b) Scenario in which the metabolites share common causes with the risk of developing other cancers. M represents the matching factors and any other measured covariates. A represents the metabolites. Y represents breast cancer. S represents selection into the case-control study, which includes the requirement to be free of other cancers at the time of selection. U represents unmeasured common causes (e.g., unmeasured genetic or lifestyle factors).

We expect that some degree of selection bias exists because, at a minimum, some unmeasured genetic or lifestyle factors are likely to affect both the metabolome and the risk of developing other cancers. However, the magnitude of this bias may be relatively small if the incidence of other cancers is low, the magnitude of the associations between the variables are small conditional on the matching factors and any other measured covariates, and/or there is little meaningful variation in the magnitude of the conditional association between other cancers and breast cancer across levels of the metabolites on the odds ratio scale.
